# Comparative Taphonomy, Taphofacies, and Bonebeds of the Mio-Pliocene Purisima Formation, Central California: Strong Physical Control on Marine Vertebrate Preservation in Shallow Marine Settings

**DOI:** 10.1371/journal.pone.0091419

**Published:** 2014-03-13

**Authors:** Robert W. Boessenecker, Frank A. Perry, James G. Schmitt

**Affiliations:** 1 Department of Earth Sciences, Montana State University, Bozeman, Montana, United States of America; 2 University of California Museum of Paleontology, University of California, Berkeley, California, United States of America; 3 Santa Cruz Museum of Natural History, Santa Cruz, California, United States of America; Raymond M. Alf Museum of Paleontology, United States of America

## Abstract

**Background:**

Taphonomic study of marine vertebrate remains has traditionally focused on single skeletons, lagerstätten, or bonebed genesis with few attempts to document environmental gradients in preservation. As such, establishment of a concrete taphonomic model for shallow marine vertebrate assemblages is lacking. The Neogene Purisima Formation of Northern California, a richly fossiliferous unit recording nearshore to offshore depositional settings, offers a unique opportunity to examine preservational trends across these settings.

**Methodology/Principal Findings:**

Lithofacies analysis was conducted to place vertebrate fossils within a hydrodynamic and depositional environmental context. Taphonomic data including abrasion, fragmentation, phosphatization, articulation, polish, and biogenic bone modification were recorded for over 1000 vertebrate fossils of sharks, bony fish, birds, pinnipeds, odontocetes, mysticetes, sirenians, and land mammals. These data were used to compare both preservation of multiple taxa within a single lithofacies and preservation of individual taxa across lithofacies to document environmental gradients in preservation. Differential preservation between taxa indicates strong preservational bias within the Purisima Formation. Varying levels of abrasion, fragmentation, phosphatization, and articulation are strongly correlative with physical processes of sediment transport and sedimentation rate. Preservational characteristics were used to delineate four taphofacies corresponding to inner, middle, and outer shelf settings, and bonebeds. Application of sequence stratigraphic methods shows that bonebeds mark major stratigraphic discontinuities, while packages of rock between discontinuities consistently exhibit onshore-offshore changes in taphofacies.

**Conclusions/Significance:**

Changes in vertebrate preservation and bonebed character between lithofacies closely correspond to onshore-offshore changes in depositional setting, indicating that the dominant control of preservation is exerted by physical processes. The strong physical control on marine vertebrate preservation and preservational bias within the Purisima Formation has implications for paleoecologic and paleobiologic studies of marine vertebrates. Evidence of preservational bias among marine vertebrates suggests that careful consideration of taphonomic overprint must be undertaken before meaningful paleoecologic interpretations of shallow marine vertebrates is attempted.

## Introduction

The robust shallow marine invertebrate fossil record has been the subject of numerous studies that have broadened the field of taphonomy from a focus on the negative aspects of preservation (taphonomic loss), to one on taphonomic gain and the power of taphonomic data for understanding depositional and biogenic processes. These studies have focused on formation of skeletal concentrations [1], effect of subsidence rate on skeletal accumulations [2]–[4], relations between stratigraphic sequence boundaries and preservation [5], development of new taphonomic field data collection methods [6], [7], comparative taphonomy [8], and recognition of taphonomic facies [9], [10].

Conversely, the taphonomic record of marine vertebrate fossils has received comparatively little study due to the relative rarity of marine vertebrate fossils (compared with invertebrates) and difficulty in conducting actualistic experiments in the marine environment [11]. Taphonomic investigations of processes affecting terrestrial vertebrates are more common because their presence at the land surface make them easier to observe and interpret. Indeed, much paleontologic research has focused on interpreting the genesis of terrestrial vertebrate bonebeds [12].

For marine vertebrates, only a few paleontologic studies have compared the trends in preservation along environmental gradients [13], [14]. A number of actualistic studies have assessed problems of decomposition, disarticulation, sorting, abrasion, scavenging, and bloating in marine vertebrates [15]–[23]. Additionally, several forensic studies focused on decomposition, disarticulation, bloating, hydraulic sorting, bone modification, and marine scavenging, using experiments with pigs or forensic case data, are applicable to marine vertebrate taphonomy [11], [24]–[28]. All of these studies were conducted in shallow water conditions along shorelines, at the ocean surface, or in the laboratory. Deeper water studies of whale-falls have generated useful taphonomic data (scavenging rates, encrustation, bioerosion) for large cetaceans in outer shelf, bathyal, and abyssal environments [29]–[32]. However, few of these studies have been conducted in the range of water depths characteristic of the continental shelf [33], and it is unclear how applicable the majority of whale-fall data are to shelf environments. Virtually no actualistic data exist for depositional environments between the shoreline and deep sea. A well-formulated taphofacies model for shallow marine vertebrate assemblages is currently lacking, and no existing actualistic or historical framework is available to place marine vertebrate fossil assemblages within a broader taphonomic context.

**Figure 1:**
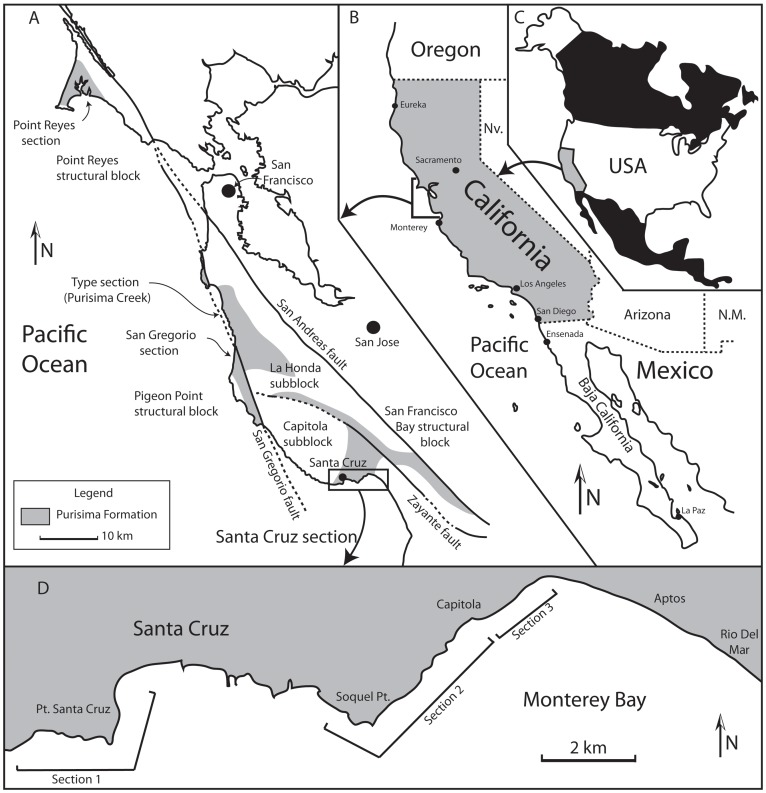
Geologic map of the Purisima Formation. (A) Generalized geologic map of Purisima Formation exposures in Northern California, modified from Boessenecker (2011). (B) Geographic location of (A) in California, and (C) map of North America showing location of (B). (D) Map of Santa Cruz county coastline showing location of cliff exposures and sections 1, 2, and 3 examined during this study. Abbreviations: ss, sandstone; sls, siltstone; ms, mudstone.

**Figure 2:**
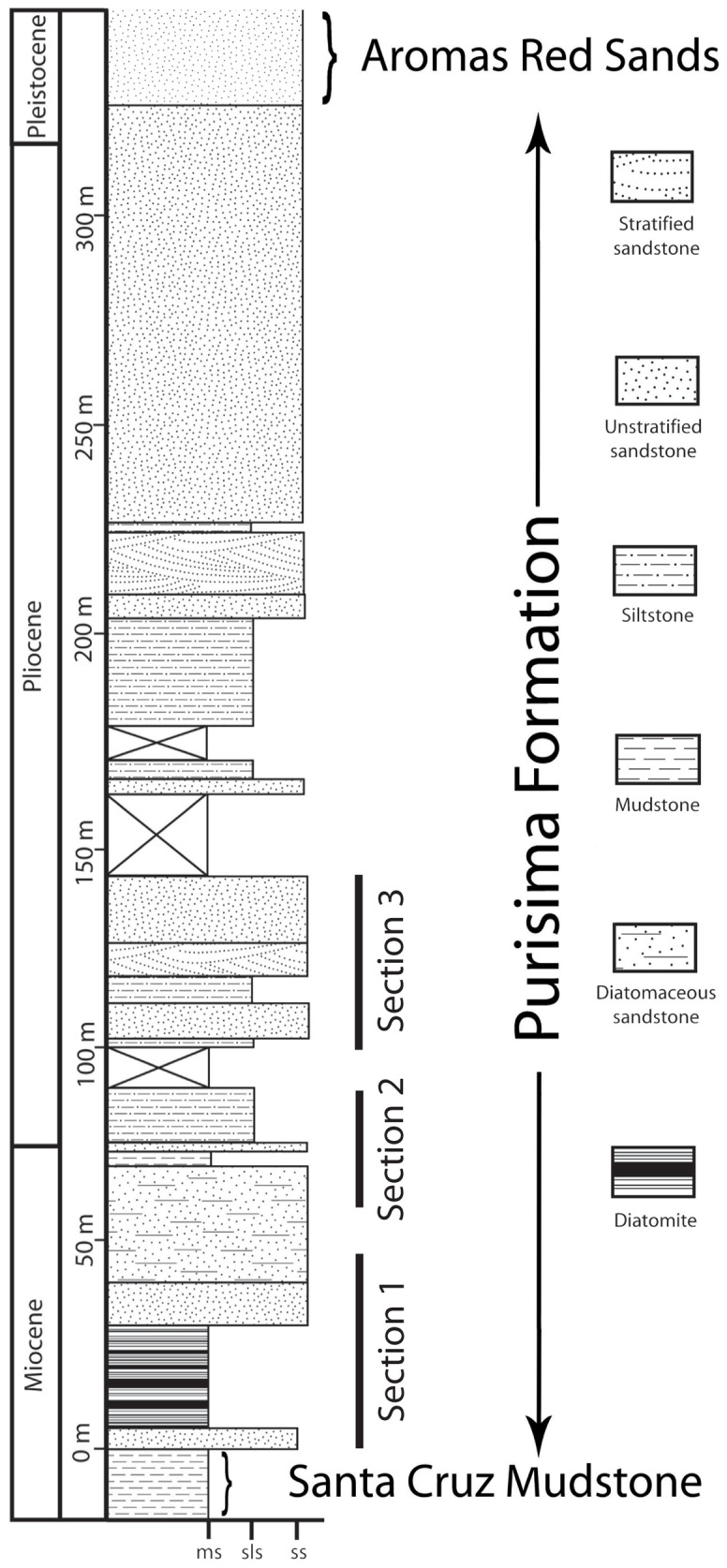
Generalized stratigraphic column showing stratigraphic position of the three sections studied. Modified from Boessenecker and Perry (2011) and Powell et al. (2007).

**Figure 3:**
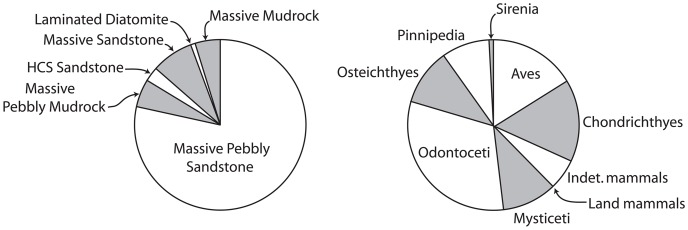
Pie charts showing proportion of specimens from each lithofacies (left) and taxon (right). Abbreviations: HCS, hummocky cross-stratified.

The richly fossiliferous late Neogene Purisima Formation of Northern California (Figs. 1–2) was deposited in depositional environments ranging from nearshore and estuarine to outer shelf and upper slope settings [34], [35]. Preservation of fossil vertebrates from different depositional settings in the Purisima Formation allows examination of onshore-offshore gradients in fossil preservation. This study takes advantage of the abundance of vertebrate skeletal material, including baleen whales, toothed whales, pinnipeds, sea cows, marine birds, bony fish, and sharks (Table 1), from numerous depositional environments (Fig. 3) represented by excellent sea cliff exposures of the Purisima Formation at the Santa Cruz section in order to: 1) document onshore-offshore trends in preservation styles and degree; 2) determine the sedimentologic (hydrodynamic) or biogenic processes controlling patterns of marine vertebrate preservation; and 3) construct a preliminary taphonomic framework for understanding marine vertebrate fossil preservation across siliciclastic shelves.

**Table 1:** Aggregate vertebrate assemblage from the Santa Cruz section of the Purisima Formation.

Chondrichthyes	Odontoceti
*Alopias* sp.^P^	Albireonidae indet.^M^
*Cetorhinus maximus*	*Denebola* sp.
*Carcharinus* sp.^P^	Delphinapterinae indet.
*Carcharocles megalodon* ^M^	Delphinidae indet.
*Carcharodon hastalis* ^M^	Globicephalinae indet. 1
*Carcharodon hubbelli*	Globicephalinae indet. 2^P^
*Carcharodon carcharias* ^P^	*Parapontoporia* sp.
*Dasyatis* sp.	*Parapontoporia wilsoni*
*Galeorhinus* sp.^P^	Phocoenidae n. g. 1
*Hexanchus* sp.	Phocoenidae n. g. 2
*Isurus oxyrhincus*	aff. Phocoenidae
*Lamna* sp.	cf. *Piscolithax* sp.^M^
*Myliobatis* sp.	Physeteroidea indet.
*Raja* sp.	**Mysticeti**
*Raja* sp. cf. *R. binoculata* ^P^	Balaenidae indet.
**Osteichthyes**	*Balaenoptera* sp., cf. *B. bertae*
*Acipenser* sp.	Balaenopteridae n. g.^P^
*Anarrhichthys* sp.	Balaenopteridae indet.
*Citharichthys stigmaeus*	“*Balaenoptera*” *cortesi* var. *portisi*
*Epinephelus* sp.	*Eubalaena* sp.
*Oncorhynchus rastrosus*	*Herpetocetus bramblei*
*Paralichthys californicus*	*Herpetocetus* n. sp.^P^
*Parophrys vetulus*	“*Megaptera*” *miocaena*
Sciaenidae indet.	*Nannocetus* sp.^M^
*Seriola* sp.	*Parabalaenoptera* sp.
**Aves**	**Sirenia**
*Alca* sp.	*Dusisiren dewana* ^M^
*Brachyramphus* sp.	Hydrodamalinae indet.^M^
*Cerorhinca* sp.	**Artiodactyla**
*Mancalla vegrandis*	Camelidae indet.
*Mancalla lucasi*	**Rodentia**
*Miomancalla wetmorei* ^M^	*Castor californicus*
*Morus humeralis*	**Perissodactyla**
*Phalacrocorax* sp.	Equidae indet.^P^
*Puffinus* sp.	
*Synthliboramphus* sp.	
**Pinnipedia**	
*Callorhinus* sp., cf. *C. gilmorei* ^P^	
*Dusignathus santacruzensis* ^M^	
*Gomphotaria* sp.^M^	
cf. *Imagotaria* ^M^	
*Thalassoleon macnallyae*	
*Valenictus* sp.^P^	

Compiled from Barnes (1976), Perry (1977B), Repenning and Tedford (1977), Domning (1978), Barnes (1985), Boessenecker and Geisler (2008), Whitmore and Barnes (2008), Boessenecker et al. (2009), Boessenecker and Perry (2011), Boessenecker et al. (2013), N. A. Smith (personal communication, 2011) and Boessenecker and Perry (unpublished data). ^M^ and ^P^ denote occurrences restricted to the late Miocene and Pliocene parts of the Santa Cruz section of the Purisima Formation (respectively).

To achieve these goals, numerous types of taphonomic, stratigraphic, and sedimentologic data were recorded for a large sample set (Table 2; Table S1) of fossil vertebrates from the Purisima Formation. Comparative taphonomy [8] was utilized to compare preservation of different marine vertebrate taxa within an assemblage, assess problems of bias and differential preservation, and to compare preservation of fossil taxa across inferred depositional settings. Taphofacies analysis [9] was employed to map preservational facies and their lateral and vertical relationships.

**Table 2:** Sample size of specimens from each taxon studied from different lithofacies of the Purisima Formation.

Taxon	Shc	Mld	Mm	Mpm	Mps	Sm	Total
Aves	3	2	5	1	142	14	166
Chondrichthyes	1	1	2	9	138	9	160
Indet. Mammal	0	0	0	2	55	4	61
Land Mammal	0	0	0	0	1	0	1
Mysticeti	4	0	13	7	73	12	109
Odontoceti	11	2	23	9	249	32	326
Osteichthyes	7	0	2	3	93	5	110
Pinnipedia	2	1	4	24	52	8	92
Sirenia	0	0	0	1	7	0	8
Total	28	6	45	60	810	84	1033

Abbreviations: Shc, hummocky cross-stratified sandstone lithofacies; Mld, laminated diatomite lithofacies; Mm, massive mudrock lithofacies; Mpm, massive pebbly mudrock lithofacies; Spm, massive pebbly sandstone lithofacies; Ms, massive sandstone lithofacies.

## Geologic Background

### General Geology

The Neogene (Miocene-Pliocene) Purisima Formation was named by Haehl and Arnold [36] for fossiliferous marine sedimentary rocks in sea cliffs at the mouth of Purisima Creek in San Mateo County, California (Fig. 1). Ranging in age from 6.9–2.47 Ma [35], [37], the Purisima Formation crops out near San Francisco and Santa Cruz, California [38], [39], where it is composed of fossiliferous marine conglomerate, sandstone, siltstone, and mudstone, and diatomite. Wrench tectonics associated with strike-slip faulting in Northern California likely controlled basin subsidence [35], [40]. The four major areas of exposure of the Purisima Formation (Fig. 1A), mostly west of the San Andreas fault include: 1) Point Reyes, Marin County, CA, formerly the “Drakes Bay Formation” of Galloway [41]; 2) Pillar Point, San Mateo County, CA [42]; 3) Half Moon Bay, San Mateo County, CA [39]; and 4) Santa Cruz, Santa Cruz County, CA [35]. Although the Purisima Formation crops out in some stream gullies and man-made exposures in the Santa Cruz Mountains [39], the majority of outcrops are in linear coastal cliffs. Because of local faulting and folding, some Purisima Formation exposures have been mapped but have yet to have detailed stratigraphic sections measured and described. Larger scale faulting (offset along the San Gregorio fault in particular) has caused problems with correlations between different exposures and across faults [43]–[45]. Vertebrate fossils (baleen whales, porpoises, beluga, walruses, fur seals, sea cows, marine birds, fish, sharks, and rays) occur in most Purisima Formation strata [43], [46]–[59]. Fossil invertebrates and microinvertebrates are abundant in most exposures of the Purisima Formation and include gastropods, bivalves, brachiopods, barnacles, decapods, echinoids, and asteroids [34], [35], [42], [45], [56], [60]–[64].

### Stratigraphy

The Purisima Formation records an overall change from the earlier biogenic sedimentation of the Middle and Late Miocene (as recorded by the Monterey Formation and Santa Cruz Mudstone), to siliciclastic deposition in Northern California during the latest Miocene and Pliocene [35]. The Tortonian-equivalent (10–12 Ma) Santa Margarita Sandstone (deposited between the Monterey Formation and Santa Cruz Mudstone) can be viewed as the first pulse of coarse siliciclastic marine sedimentation in this region during the Late Neogene. The underlying Santa Cruz Mudstone represents offshore biosiliceous sedimentation [65], and was already lithified and deformed at the time that Purisima Formation deposition began [66]. The Purisima Formation represents an overall regression that is punctuated by several transgressive-regressive successions [35].

**Figure 4:**
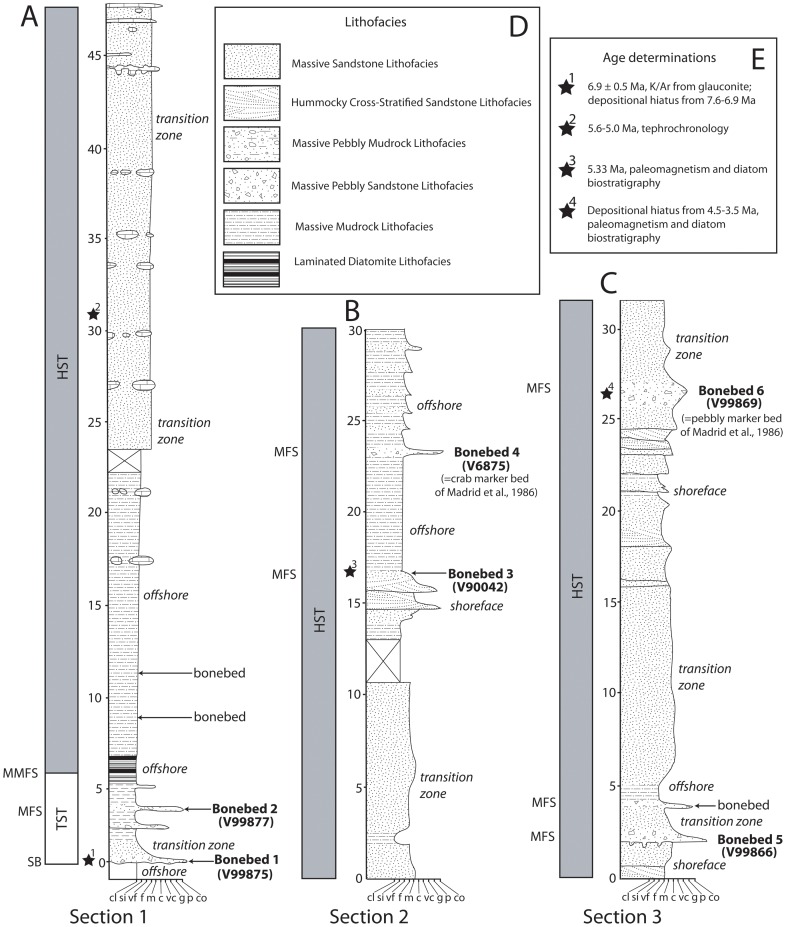
Stratigraphic columns, depositional setting, and sequence interpretation of the Purisima Formation. Measured sections depicted include section 1 (A), section 2 (B), and section 3 (C). Key to lithofacies in (D). Age determinations (stars) listed in (E), and ages from Madrid et al. (1986), Aiello et al. (2001) and Powell et al. (2007). Vertical bar denotes sequence stratigraphic units; TST, transgressive systems tract; HST, highstand systems tract; MMFS, maximum marine flooding surface; MFS, marine flooding surface. Interpretations of depositional setting in italics. UCMP locality numbers labeled in parentheses for individual bonebeds. Vertical thickness in meters.

The Santa Cruz section of the Purisima Formation was designated as a supplementary reference section by Powell et al. [35] because it “represents the most continuously exposed and best dated Purisima section.” The type section, exposed in cliffs near the mouth of Purisima Creek near Half Moon Bay, is no longer accessible by foot. Other sections (Point Reyes, San Gregorio, Seal Cove, and Año Nuevo sections) have only received cursory study, and the age of some of these sections remains uncertain [35], [45]. The Santa Cruz section is 325 meters thick, exposed for 19 km of shoreline along the northern margin of Monterey Bay (Fig. 1A, D) [35]. Overall, this section comprises a shoaling-upwards stratigraphic trend with diatomite and mudrock in the lower portion, bioturbated sandstone dominating the middle, and cross-stratified sandstone and coquina in the upper portion [34], [35] (Figs. 2, 4). These deposits represent offshore to shoreface, foreshore, and estuarine deposition [34], [35]. Nine distinct bonebeds occur within the lower and middle parts of the Santa Cruz section, but only six were accessible or satisfactorily exposed for study, here numbered Bonebeds 1–6 (Fig. 4).

### Age

The age of the Santa Cruz section is well constrained, based on several methods. This section ranges from latest Miocene at the base, to middle-late Pliocene at the top [35] (Fig. 2); the other sections of the Purisima Formation are approximately this age as well. The basal glauconitic sandstone yielded a K/Ar date of 6.9±0.5 Ma [37]. The diatom-bearing lower 90 meters of the Purisima Formation yielded diatom assemblages indicating a similar age, 7–5 Ma [35]. A paleomagnetic study of the Purisima Formation indicates the Santa Cruz section is 6.07 to 2.47 Ma in age, with a depositional hiatus from 4.5 to 3.5 Ma [37]; this depositional hiatus is marked by one of the bonebeds investigated by this study.

### Previous Taphonomic Work

Norris [34] investigated preservation of invertebrate remains in the Purisima Formation, and found a shift from physical processes (i.e. reworking, transport) dominating shallower marine settings, to ecological processes (i.e. bioturbation, encrustation, in-situ preservation) predominant in deeper marine settings. Norris [34] also observed a decrease in thickness and frequency of invertebrate accumulations with increasing inferred water depth. A study by Boessenecker and Perry [52] identified juvenile fur seal bones with tooth marks attributable to marine mammal teeth from the middle part of the Santa Cruz section.

## Methods

This study focused exclusively on the sea cliff exposures of the Purisima Formation at the Santa Cruz supplementary reference section. To establish paleoenvironmental gradients of preservation within the Purisima Formation, a stratigraphic framework was first established and ‘populated’ with taphonomic data. A combination of sedimentologic, stratigraphic, and taphonomic methods were utilized and are summarized below. The upper portion of the Santa Cruz section was not studied in detail due to its limited exposure and the rarity of vertebrate remains.

### Sedimentologic and Stratigraphic Methods

To place the vertebrate fossil assemblages of the Purisima Formation into proper stratigraphic and sedimentologic context, several methods were employed. Three sections representing the lower and middle portions of the Santa Cruz section (*sensu* [35]) of the Purisima Formation were measured and described (Figs. 1–2, 4). These sections do not overlap, and represent only part of the lower Santa Cruz section of the Purisima Formation (Fig. 2); measurement and description of a continuous section was not possible due to dangerous outcrop conditions. Data regarding bed thickness and geometry, lithology, sedimentary structures, bedding contacts, ichnofabric index [67] and ichnofossil content were collected for each bed. These data were then utilized to delineate lithofacies (Table 3), with each interpreted relative to hydrodynamic (energy) and substrate conditions. The interpretations are based on inferences of the bedforms and substrate conditions that characterized development of each lithofacies. Commonly co-occurring lithofacies were grouped into lithofacies associations representative of environments characterized by specific related suites of sediment transport processes and substrate characteristics. To the degree possible, bounding surfaces were also noted during section measurement and description; these surfaces were utilized in conjunction with lithofacies associations to develop a sequence stratigraphic framework for interpreting controls on Purisima Formation deposition.

**Table 3:** Comparison of lithofacies characteristics.

Lithofacies Characteristics	Massive Pebbly Sandstone Lithofacies (Spm)	Massive Sandstone Lithofacies (Sm)	Hummocky Cross Stratified Sandstone Lithofacies (Shc)	Massive Pebbly Mudrock Lithofacies (Mpm)	Massive Mudrock Lithofacies (Mm)	Laminated Diatomite Lithofacies (Mld)
Lithology	V. Fine-Medium Sand, brown-blue gray, weathering to light brown, tan, light gray	V. Fine-Medium Sand, blue-gray weathering to light brown-tan, moderately-poorly sorted	V. Fine-Medium Sand, brown-blue gray, weathering to light brown-tan, well-moderately sorted	Silt-Clay, brown to blue gray, weathering to light brown-tan to light gray, poorly sorted	Silt-Clay, blue-gray weathering to light gray, poorly sorted	Clay, tan-yellow and gray laminations, weathering to light tan
Geometry	Tabular, pinches and swells	tabular	tabular	Tabular, pinches and swells	tabular	tabular
Sedimentary structures	Massive bedding	Massive bedding	Hummocky cross stratifications	Massive bedding	Massive bedding	Planar laminations
Trace fossil content & Ichnofacies	Cross-cutting *Ophiomorpha* abundant, *Gastrochaenolites* and *Trypanites* uncommon; *Skolithos* and *Trypanites* ichnofacies	Cross-cutting *Ophiomorpha* and *Planolites* abundant; *Skolithos* & *Cruziana* ichnofacies	Rare-uncommon *Ophiomorpha* in upper parts of HCS beds; *Skolithos* ichnofacies	Cross-cutting *Ophiomorpha*, *Thalassinoides*, *Teichichnus*, *Planolites* abundant; *Ophiomorpha* below bonebeds infilled with bonebed debris; *Skolithos* and *Cruziana* ichnofacies	Cross-cutting *Teichichnus*, *Planolites* abundant, rare *Thalassinoides* and *Ophiomorpha*; *Cruziana* ichnofacies	Rare *Planolites*; uncertain ichnofacies
Ichnofabric Index	II 5	II 4–5	II 1–3	II 5	II 4–5	II 1
Erosional surfaces	Moderate	Rare	Abundant	Rare	Rare	Rare
Phosphatic Clasts/Bioclasts	Abundant, nodular; many occur as phosphatic overgrowths on vertebrate bones/teeth, crustaceans, or as external/internal molds of mollusks	Rare, nodular; many occur as phosphatic overgrowths on vertebrate bones/teeth, crustaceans, or as external/internal molds of mollusks	Moderate, nodular; some occur as phosphatic overgrowths on vertebrate bones/teeth, crustaceans, mostly as external/internal molds of mollusks	Abundant, entirely nodular	Rare, nodular; often occur as in-situ nodules; often as overgrowths on vertebrate and mollusk elements and crustacean exoskeletons	Absent
Relative thickness in study sections	3.8%	58.2%	5.1%	1.7%	28.7%	2.5%
Invertebrate abundance	Moderate-Abundant	Moderate	Abundant	Rare	Rare	Absent
Vertebrate abundance	Abundant	Moderate	Moderate	Abundant	Rare	Rare
Bioclastic content	Abundant vertebrate skeletal elements in thin bonebeds; abundant invertebrate shells in some bonebeds; densely to loosely packed	Isolated vertebrate bones and teeth, occasional associated/articulated skeletons; dispersed; occasional pavements and stringers of mollusk shells	Isolated vertebrate bones and teeth, mollusk shell concentrations form pavements, stringers, and thin beds at base of HCS beds; vertebrates dispersed, invertebrates densely packed to dispersed	Abundant vertebrate skeletal elements in thin bonebeds; invertebrates absent	Isolated vertebrate bones and teeth, occasional associated/articulated skeletons; dispersed; occasional clumps, pavements and stringers of mollusk shells	Isolated vertebrate skeletal elements, dispersed; rare, isolated mollusks
Interfingering relationships	Shc, Sm, Mm	Shc, Spm, Mm	Sm, Spm, Mm	Mm	Shc, Spm, Sm, Mpm, Mld	Mm
Facies Association	Shoreface Facies Association	Shoreface Facies Association	Shoreface Facies Association	Offshore Facies Association	Offshore Facies Association	Offshore Facies Association
Interpretation	Bonebed formation in shoreface and transition zone by hiatus, truncation and sediment bypass during transgression (or forced regression); generally high energy conditions; glauconite, phosphate nodules, and hardgrounds/firmgrounds forming during depositional hiatus	Deposition below storm weather wave base in transition zone; pervasive bioturbation of sediment owing to infrequent current disturbance and slow sedimentation	Frequent high-energy hyperpycnal deposition above storm weather and fair weather wave base in shoreface; frequent reworking	Bonebed formation in offshore settings by hiatus, truncation and sediment bypass during transgression; higher energy conditions than other mudrock lithofacies; phosphate nodules form during depositional hiatus	Suspension fallout of silt and clay under low energy conditions well below storm weather wave base, distal to limits of sand delivery to transition zone; deposition in offshore on outer shelf	Low energy biosiliceous sedimentation at or near the shelf-slope break well below storm weather wave base, dysoxic to anoxic pore waters

Ichnofabric index after Droser and Bottjer (1989).

### Traditional Taphonomic Methods

To study the taphonomy of each fossil assemblage, the methods outlined by Kidwell et al. [6] and Kidwell and Holland [7] for characterization of bioclast concentration geometry and architecture were applied to all bioclastic (invertebrate or vertebrate rich) units. Detailed field descriptions of the lithology (including clast counts) and sedimentary architecture were recorded for bonebeds, and a large sample of specimens were collected from these bonebeds (Table 2). Bonebeds were recognized as relative concentrations of vertebrate skeletal material [12] and examined along strike to determine their lateral extent and changes in character. Recognition of bonebeds based upon a percentage composition of vertebrate skeletal material (e.g. [68]–[70]) was avoided because 1) private collection of vertebrate fossils would artificially deflate the size of the vertebrate fraction, perhaps making bonebeds reported herein fall under the minimum threshold value for bonebed recognition; and 2) bulk sampling of all bonebeds was not possible because of safety issues. Taphonomic data (see below) were collected for a large sample (n = 1033; Fig. 3; Table 2; Table S1) of vertebrate fossils. These include specimens collected by R. W. Boessenecker (n = 478) from 2004–2010. Data including taxon (e.g., Chondrichthyes, Osteichthyes, Aves, Pinnipedia, Odontoceti, Mysticeti, Sirenia), abrasion, fragmentation, articulation, phosphatization, and the associated lithofacies were collected for each specimen. This study utilized fossils from museum collections (collected primarily by F.A. Perry) at the Santa Cruz Museum of Natural History (n = 188; SCMNH) and the University of California Museum of Paleontology (n = 295; UCMP) of known stratigraphic provenance (to a distinct bonebed, or distinct stratigraphic position assigned to one of the included lithofacies, based on collector’s field notes). An additional 72 uncurated fossils (also with known provenance) from UCMP and SCMNH collections were recorded. Specimens lacking clear stratigraphic provenance were excluded. Vertebrate fossils were identified to each taxonomic group based on comparisons with previously published Neogene marine vertebrate fossil descriptions and photographs of modern osteological specimens. Vertebrate taxa studied include Chondrichthyes (sharks and rays), Osteichthyes (bony fishes), Aves (birds), Pinnipedia (seals, sea lions, and walruses), Mysticeti (baleen whales), Odontoceti (dolphins and other toothed whales), Sirenia (sea cows), and land mammals (Table 1, 2). Many bone fragments with typical mammalian histology (i.e. cancellous bone) not confidently assignable (due to taphonomic damage or recent erosion) to any of the aforementioned mammalian groups, and too large to represent birds or bony fish, were identified as indeterminate mammals.

**Figure 5:**
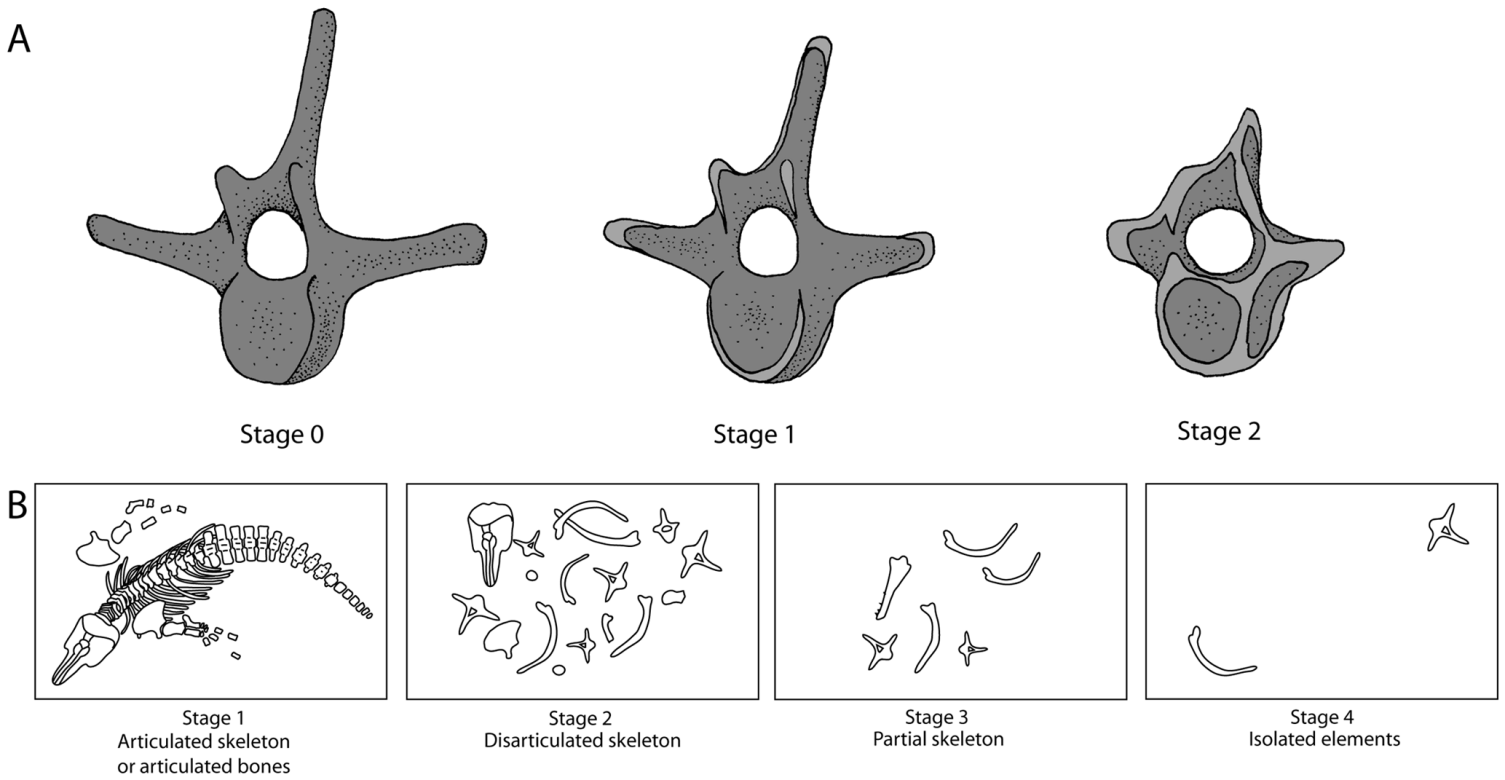
Scales for abrasion (A) and articulation (B) used in this study. Light gray areas in (A) indicate abraded surfaces.

**Figure 6:**
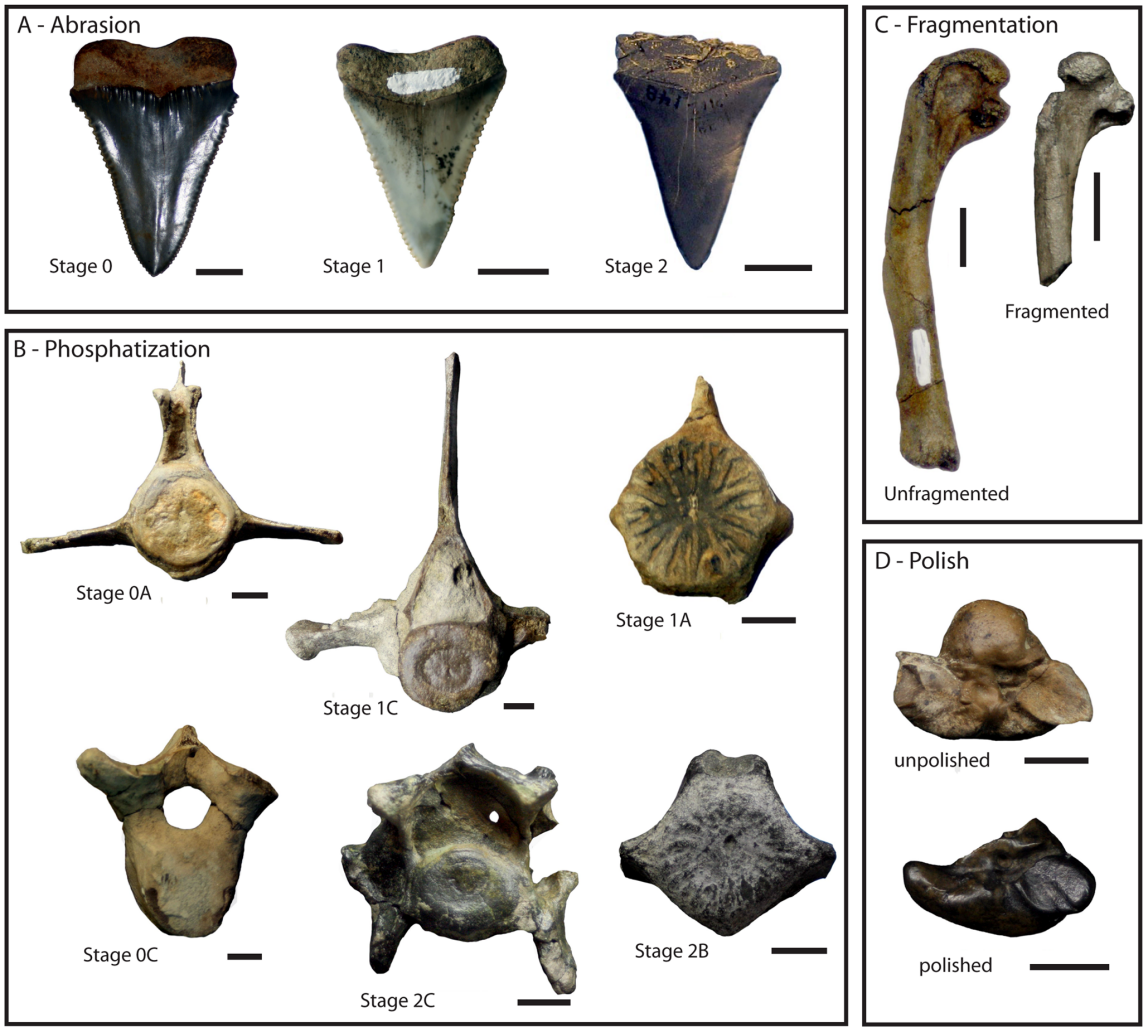
Examples of bone modifications on representative vertebrate fossils from the Purisima Formation. (A) teeth of *Carcharodon carcharias* (Chondrichthyes, Lamnidae) showing abrasion stages 0–2. (B) Odontocete (Cetacea, Odontoceti) vertebrae showing various phosphatization stages. (C) Auk humeri (Aves, Alcidae; *Mancalla vegrandis* on left) showing presence and absence of fragmentation. (D) Odontocete petrosals (ear bones; Cetacea, Odontoceti; *Parapontoporia wilsoni* on top, Phocoenidae indet. below) displaying presence and absence of polish. Scale bars equal 1 cm.

**Figure 7:**
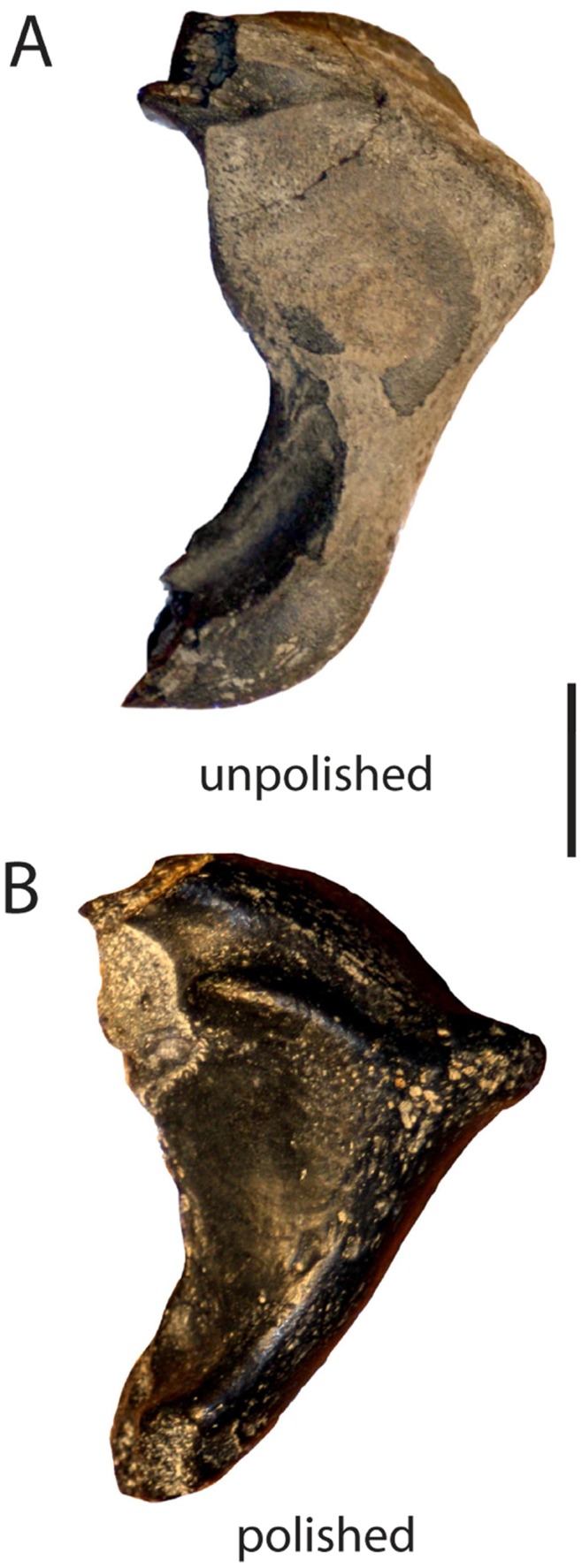
Examples of polished elements. Partial odontocete atlas vertebra (A) lacking polish and (B) with polish. Scale bar = 1 cm.

A modified version of Fiorillo’s [71] abrasion scale was used (Fig. 5A). The modified scale includes three stages: unabraded (Stage 0), lightly abraded (Stage 1), and heavily abraded (Stage 2). Although elaborate fragmentation scales have previously been published, only presence/absence of fragmentation was documented (Fig. 6C). Articulation and element association was coded on a simple scale (Fig. 5B): 1 = articulated skeleton (or articulated elements); 2 = disarticulated skeleton; 3 = cluster of a few associated or articulated elements; 4 = isolated element. Additionally, rare cases of biogenic bone modification such as bite marks and invertebrate bioerosion were noted for individual specimens. Although commonly used in terrestrial taphonomic studies, Behrensmeyer’s [72] bone weathering scale was not employed because analogous weathering attributes have not been recognized for marine vertebrate fossils [29], [73]. When present, mosaic surface cracking was noted (n = 24; 2.3%) although its significance in marine weathering of bone requires further study. Lastly, polish (light abrasion of element surface resulting in shiny, reflective and often smooth surface) was simply recorded as present or absent (Fig. 6D, 7). These data were imported into a spreadsheet, and calculated percentages were used to generate histograms and pie charts of taphonomic characteristics in relation to lithofacies, taxon, and skeletal element group (bone, calcified cartilage, earbones, and teeth).

### New Taphonomic Methods

#### Phosphatization scale

**Figure 8:**
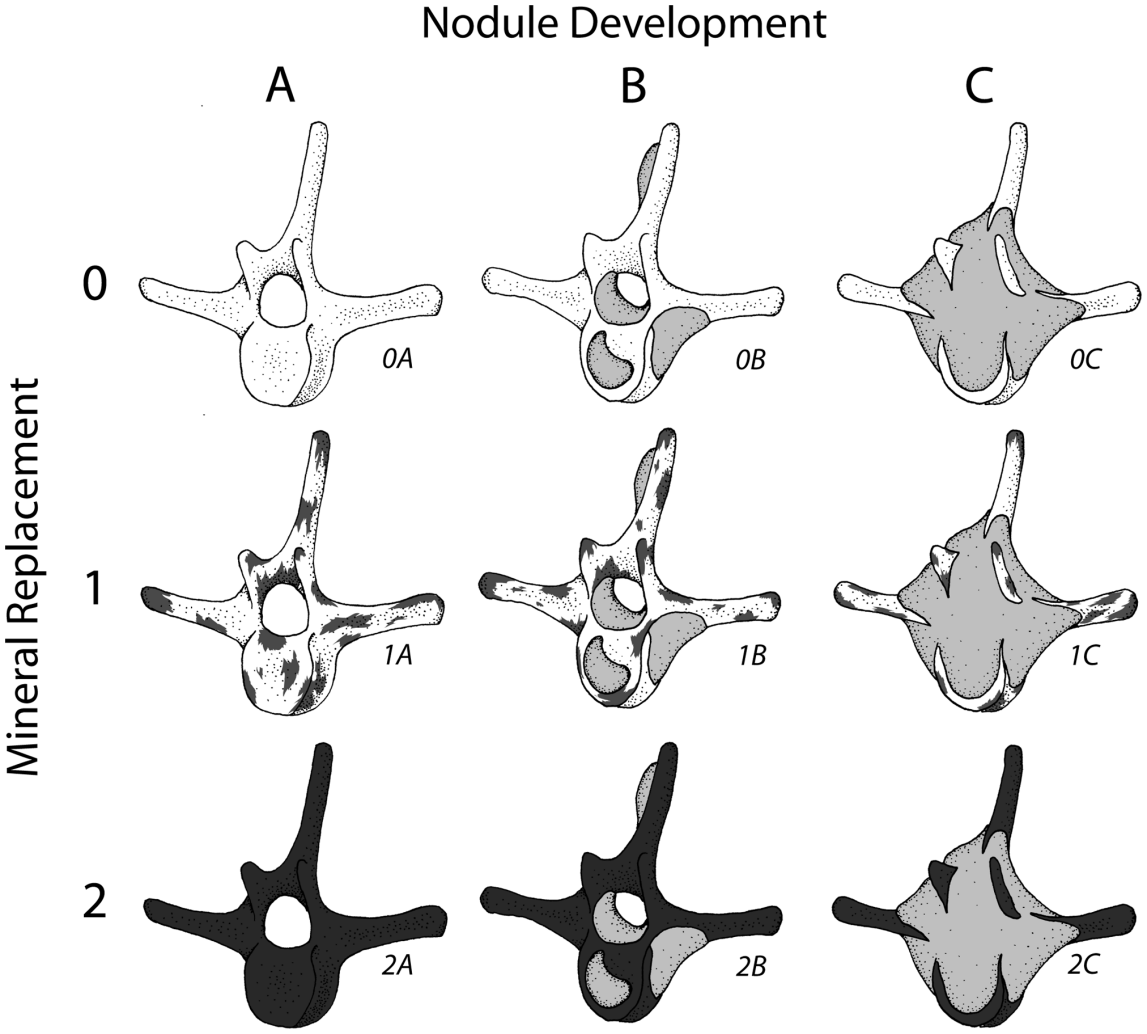
Diagrammatic representation of phosphatization scale developed for this study. Phosphate replacement shown on vertical axis (stage 0X–2X), and nodule development shown on horizontal axis (stage XA–XC), with each stage shown below hypothetical odontocete vertebra. Increasing phosphatization is generally toward lower right.

A qualitative scale to assess phosphatization of skeletal elements was devised for this study (Fig. 8). Phosphatization is an early diagenetic process that affects sediment and bioclasts at or below the sediment-water interface during times of phosphogenesis [74]–[76]. Phosphatic rinds may form at the sediment-water interface, but formation of phosphatic nodules occurs below the sediment-water interface [74]–[76]. Vertebrate skeletal elements may be phosphatically permineralized, and may also exhibit adhering phosphatic matrix (usually equivalent to mudrock in terms of grain size) or nodules (Fig. 6B), which in most cases exhibit a differing grain size from the surrounding sediment [74]. Early diagenetic permineralization of skeletal tissues and development of adhering phosphatized nodules indicate phosphatization represents a mode of prefossilization [77]. Prefossilization is here defined as early diagenetic permineralization of a bioclast prior to final burial; thus, the presence of prefossilized material within bioclastic concentrations implies that the prefossilized material was exhumed from a temporary deposit (where it underwent early diagenesis).

The phosphatization scale incorporated two qualitative measures: 1) extent of bone permineralization; and 2) occurrence and relative size of adhering phosphatic matrix or nodules (Fig. 8). Many bones and teeth in the Purisima Formation exhibit varying degrees of phosphate mineral replacement, ranging from heavy, blackened elements, contrasting with lighter shades of gray and brown in non-phosphatized elements. A simple scale was devised to reflect this (Fig. 8): no phosphate replacement (Stage 0), small patches or incomplete phosphate replacement (Stage 1), and complete phosphate replacement (Stage 2). Many of these elements also exhibit varying degrees of adhering phosphatic matrix. To capture this variation, another scale was superimposed on the mineralization scale to indicate the following: no adhering phosphatic matrix (Stage XA), limited adhering phosphatic nodule (Stage XB), and adhering phosphatic nodule covering more than one-third of the element surface area (Stage XC). This resulted in the following possible combinations: Stage 0A, 0B, 0C, 1A, 1B, 1C, 2A, 2B, and 2C. For example, a completely unphosphatized element represents Stage 0A, while 2C represents a blackened element embedded within a phosphatic nodule. All of the other possible stages represent intermediate conditions (Fig. 8). An obvious limitation of this scale is that for bone mineralization (e.g., Stage 0–2X), color change associated with phosphatization must be present and known in an assemblage. Although the color of phosphatized elements in the Purisima Formation is typically black and dark brown, this may vary from formation to formation (or even locality). Effective use of this scale should only be attempted when the color of phosphatized material is established and different from that of non-phosphatized material. Because of the large sample size (n = 1033), petrographic confirmation of phosphate replacement was beyond the scope of this study.

#### Descriptive scheme for bonebed architecture

**Figure 9:**
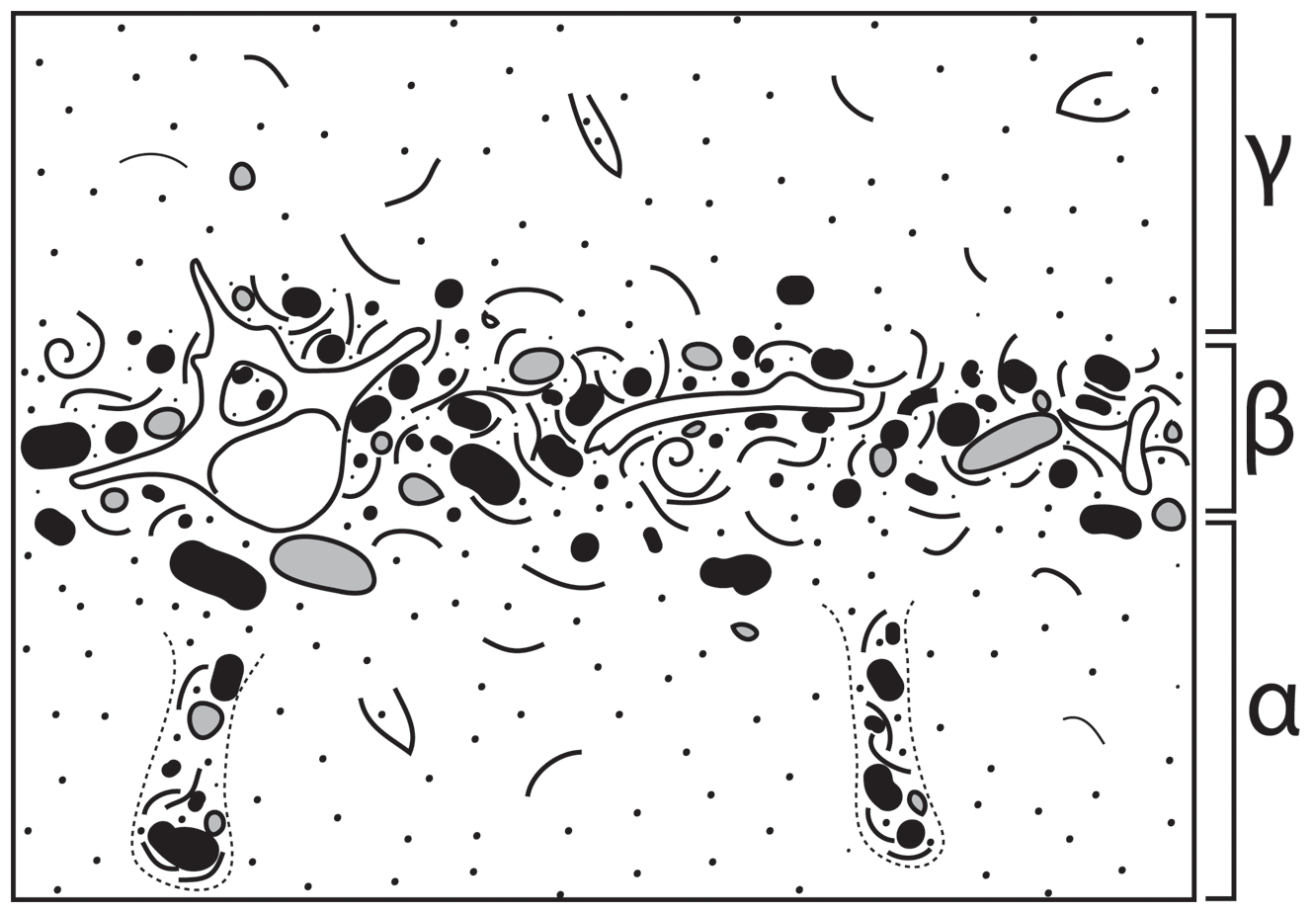
Diagrammatic cross-section of bonebed architectural divisions used in this study, showing three recurring intervals (α, β, and γ). Solid pebbles are phosphatic clasts; open pebbles are terrigenous clasts; concave and spiral lines are mollusks. *Ophiomorpha* infilled with bonebed debris shown in α-interval.

During this project several consistently recurring patterns of bonebed geometry required development of a new bonebed descriptive scheme to facilitate their interpretation. All Purisima Formation bonebeds contain three intervals (Fig. 9): 1) a lower interval occasionally characterized by an upward increase in bioclast packing (*sensu* [6]); 2) a middle interval where bioclast packing is persistently highest; and 3) an upper interval marked by an upward decrease in bioclast packing. For convenience, these intervals were assigned upper case Greek letters for alpha, beta, and gamma (Fig. 9) and termed the α-interval (lowest), β-interval (middle), and γ-interval (uppermost). This scheme specifically uses Greek rather than Latin alphabet characters so as not to be confused with soil horizon descriptive schema; this descriptive scheme may be modified and applied to terrestrial bonebeds, which occasionally coincide with paleosols. Recognition of consistent patterns of bonebed attributes facilitates their description and interpretation. For example, often there may be a sharp erosional surface at the base of the β-interval; the β-interval may also be characterized by multiple erosional surfaces. The α-interval may be barren, or may only have bonebed debris (bonebed bioclastic and clastic material) concentrated within vertical trace fossils and burrows (e.g., *Ophiomorpha*). The different intervals are often characterized by subtle changes in grain and bioclast size, packing, bioclast mineralogy, as well as changes in vertical thickness and geometry along strike.

## Lithofacies Analysis

**Figure 10:**
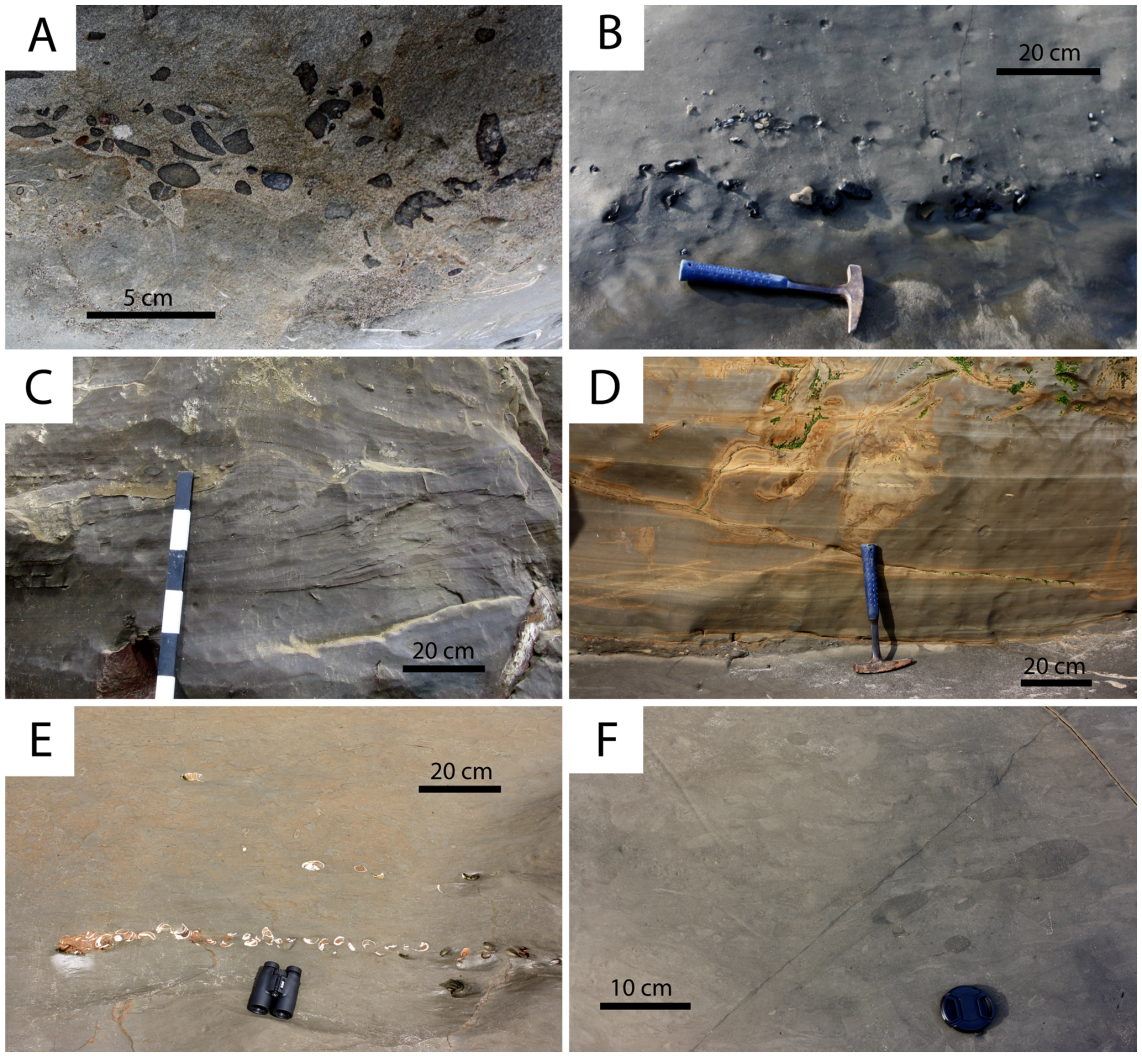
Lithofacies of the Purisima Formation delineated in this study. (A) massive pebbly sandstone (Spm). (B) massive pebbly mudrock (Mpm). (C) hummocky cross-stratified sandstone (Shc). (D) laminated diatomite (Mld). (E) massive sandstone (Sm). (F) massive mudrock (Mld).

Six lithofacies were identified in the Purisima Formation using differences in grain size, sorting, sedimentary structures, and ichnofossil content (Table 3; Fig. 10). Three sandstone lithofacies (massive pebbly, massive, and hummocky cross-stratified sandstone), and three mudrock lithofacies (massive pebbly mudrock, massive mudrock, and laminated diatomite) are present. Some of these are similar to those lithofacies identified by Norris [34].

### Sandstone Lithofacies

#### Description

The ***massive pebbly sandstone (Spm)*** lithofacies consists of thin beds of structureless fine-very coarse grained, poorly sorted sandstone with abundant glauconite sand grains and phosphatic components (Fig. 10A). Pebble- and rare cobble-size clasts and bioclasts comprising phosphatic nodules, vertebrate elements, and terrigenous lithic clasts (granules to pebbles) are present. Although typically loosely packed, clasts and bioclasts are occasionally densely packed within the β-interval of bonebeds (e.g., Bonebed 5) and more dispersed within the γ-interval.

Phosphatic mollusk steinkerns, phosphatized crustacean remains, and crustacean-bearing nodules comprise a large fraction of the phosphatic nodules. Vertebrate material is abundant and includes fossils of sharks (teeth, calcified cartilage), fish (bones), birds (bones), and marine mammals (bones and teeth). Calcareous mollusk shells are rare, but mollusk steinkerns comprising articulated bivalves or gastropods lacking original shell material are abundant. Less commonly, this lithofacies contains larger phosphatic nodules with a bioclastic framework of disarticulated mollusks, similar in fabric to shelly bioclastic units in underlying strata. Pebble- and cobble-sized clasts of phosphate and reworked porcelanitic pebbles and cobbles of the Santa Cruz Mudstone (Bonebed 1 only) occasionally exhibit bivalve borings up to 3 cm long, and 0.5–1.0 cm in external diameter; boring intensity is highest in extraformational Santa Cruz Mudstone clasts.

Trace fossils are abundant in this lithofacies. Burrows of *Ophiomorpha* (vertical, 3–5 cm wide, tube-shaped, probable crustacean burrows; [78]) extending downward as much as 3 meters below this facies are often filled with phosphatic pebbles and bioclastic debris identical to that preserved in overlying bonebeds. Erosional surfaces within this facies may contain similar small flask-shaped clam borings (*Gastrochaenolites*; 1–4 cm deep, flask-shaped borings from endolithic bivalves; [79]), and *Gastrochaenolites* and *Trypanites* (>1 cm wide subcylindrical borings; [79]) borings may be present on terrigenous and phosphatic clasts (but not bones).

**Figure 11:**
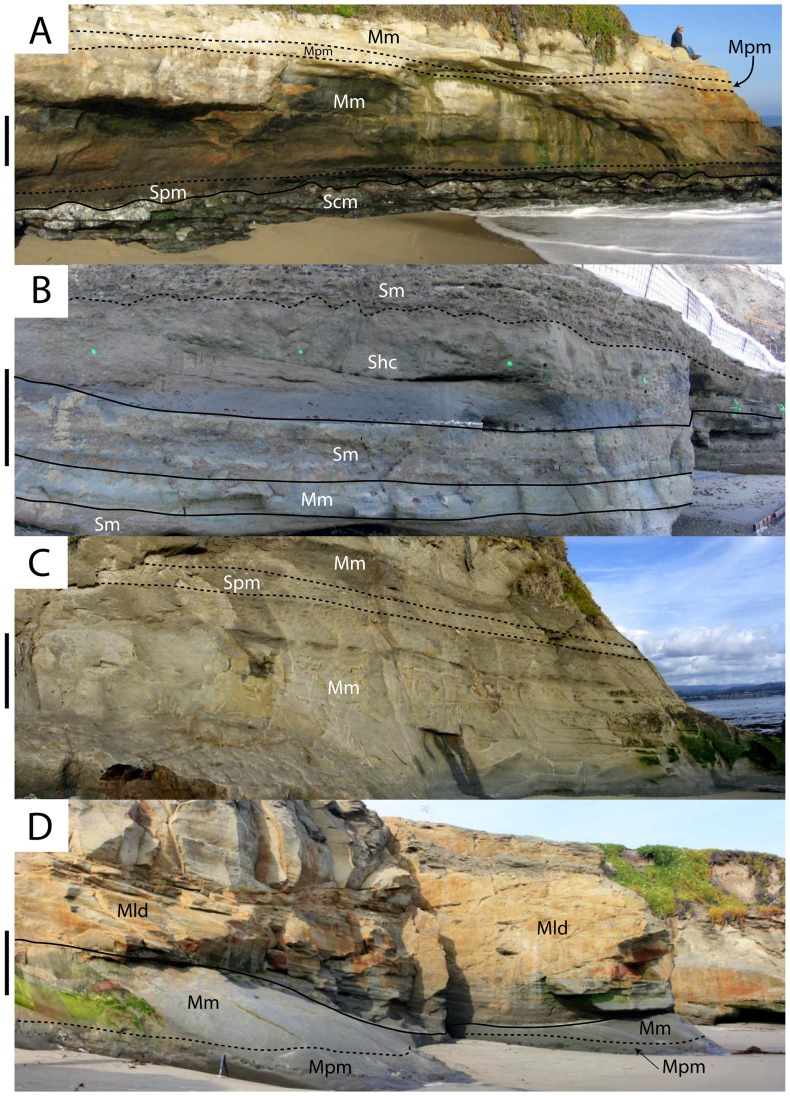
Outcrop photos showing interfingering relationships of lithofacies within the Purisima Formation. Vertical scale = 1 m. (A) exposure of the base of section 1, including Bonebed 1 (at base of cliff) and Bonebed 2 (near top of cliff). (B) exposure of section 2 at Bonebed 3 (near top of photo). (C) Exposure of section 2 at Bonebed 4 (in upper third of cliff). (D) exposure of Bonebed 2 (at base of cliff) in section 1. Solid lines denote sharp contacts, and dashed lines denote gradational contacts. Abbreviations: Mld, laminated diatomite; Mm, massive mudrock; Mpm, massive pebbly mudrock; Shc, hummocky cross-stratified sandstone; Sm, massive sandstone; Spm, massive pebbly sandstone; Scm, Santa Cruz Mudstone.

Spm units are generally tabular with basal erosional surfaces that may be sharp, gradational, or a combination of both. In one case (Bonebed 6), this lithofacies is developed below a complex phosphatic hardground with multiple erosional surfaces preserved within a few tens of centimeters (vertically). The Spm lithofacies is typified by gradational upper contacts (pebbles and bioclasts become less common and smaller up section). This lithofacies interfingers with the hummocky-cross stratified sandstone (Shc) lithofacies, massive sandstone (Sm), and occasionally the massive mudrock (Mm) lithofacies described below (Fig. 11A–C). It occurs within laterally extensive (up to several km) bonebeds (Bonebeds 1, 4, 5, and 6), and constitutes the major lithofacies of bonebeds with sand-size matrix (some bonebeds occur within mudrock facies).

The ***hummocky-cross stratified sandstone (Shc)*** lithofacies comprises beds of hummocky cross-stratified, very fine-medium grained, well-moderately sorted sandstone (Fig. 10C). These beds are typically 20–60 cm thick but range up to 120 cm in thickness [34]. Each bed fines upward, with a discontinuous shell lag present often developed at lower bounding surfaces that are sharp and often wavy. Mollusk shell concentrations typically comprise beds and pavements, with mudrock rip-up clasts (typically 1–3 cm in size, and up to 25 cm), phosphate nodules (typical of thicker shell lags), and rare vertebrate elements also present. Many shells retain adhering mudrock and phosphatic matrix. Terrigenous siliciclastic pebbles occur occasionally along the lower erosional contact, but are much rarer than in the massive pebbly sandstone (Spm) lithofacies. Bioturbation and trace fossils are absent from the lower part of each bed, but burrowing intensity increases towards the top, which is often completely bioturbated and massive. Trace fossils include rare *Ophiomorpha*. Thinner beds (<40 cm) often lack trace fossils. Shc beds are tabular and can be traced laterally for hundreds of meters [34]. This lithofacies interfingers with the massive sandstone (Sm), massive pebbly sandstone (Spm), and occasionally massive mudrock (Mm) lithofacies (Fig. 11B).

The ***massive sandstone (Sm)*** lithofacies consists of structureless tabular sandstone beds that characterize many Purisima Formation exposures. These massive sandstones are typically fine-medium grained (occasionally very fine grained), moderately-poorly sorted, and contain silty matrix (Fig. 10E). In some cases the Sm lithofacies occurs in thick (up to 25 meters thick), monotonous, unfossiliferous sections. Few erosional surfaces are preserved within this lithofacies, and most observed internal changes in lithology (i.e. color, sorting, grain size, ichnofabric) are subtle and gradational. A few thin hummocky cross-stratified sandstone (Shc) beds occur where this lithofacies grades into the hummocky cross-stratified sandstone lithofacies. The massive nature of this lithofacies derives from pervasive bioturbation that has completely homogenized the primary sedimentary fabric. Typically the trace fossil *Ophiomorpha* is abundant, with the ichnofabric often composed entirely of cross-cutting, overlapping trace fossils. *Teichichnus* (concave up vertically migrating spreiten), *Skolithos* (vertical tube-shaped burrows <1 cm wide), and *Planolites* (small horizontal tube-shaped burrows <2 cm wide) traces are also common [63]. Bioclast-rich portions are rare within this lithofacies, and primarily include thin shell beds, pavements, and stringers. Clumps of articulated bivalves (often *Anadara trilineata*) in apparent life position also occur [34]. “Articulated” clumps of the colonial gastropod *Crepidula* are rarely present. This lithofacies often directly overlies laterally extensive bonebeds [34]. Bonebed 3 lacks abundant phosphatic and terrigenous pebbles and is instead composed of this lithofacies (rather than the massive pebbly sandstone (Spm) lithofacies). Vertebrate fossils are rare within this lithofacies; when present, preservation varies from abraded to pristine isolated elements and disarticulated to partially articulated skeletons. This lithofacies interfingers with the hummocky cross-stratified sandstone (Shc), massive mudrock (Mm), and pebbly massive sandstone (Spm) lithofacies (Fig. 11B).

#### Interpretation

The ***massive pebbly sandstone (Spm)*** lithofacies (present only in some bonebeds) forms only during bonebed genesis. Abundance of glauconite indicates low to zero-net sedimentation under conditions of sediment starvation [80]. Because phosphatic nodules develop only below the sediment-water interface during periods of low to zero net sedimentation [75], their presence indicates erosion and exhumation from below the sediment substrate. Some bonebeds exhibit sharp erosional bases (Bonebeds 1, 5, and 6), whereas others (Bonebeds 2, 3, and 4) exhibit gradational contacts. The abundance of phosphatic debris within Bonebeds 2 and 4 suggests that an erosional lower contact was once present but subsequently erased by bioturbators. Clasts of lithified underlying Santa Cruz Mudstone [66] resulted from erosion of a marine rockground during the hiatus prior to Purisima Formation deposition, preserving the sharp lower contact of Bonebed 1; in contrast, formation of a phosphatic hardground preserved the sharp internal contacts of Bonebed 6.

**Figure 12:**
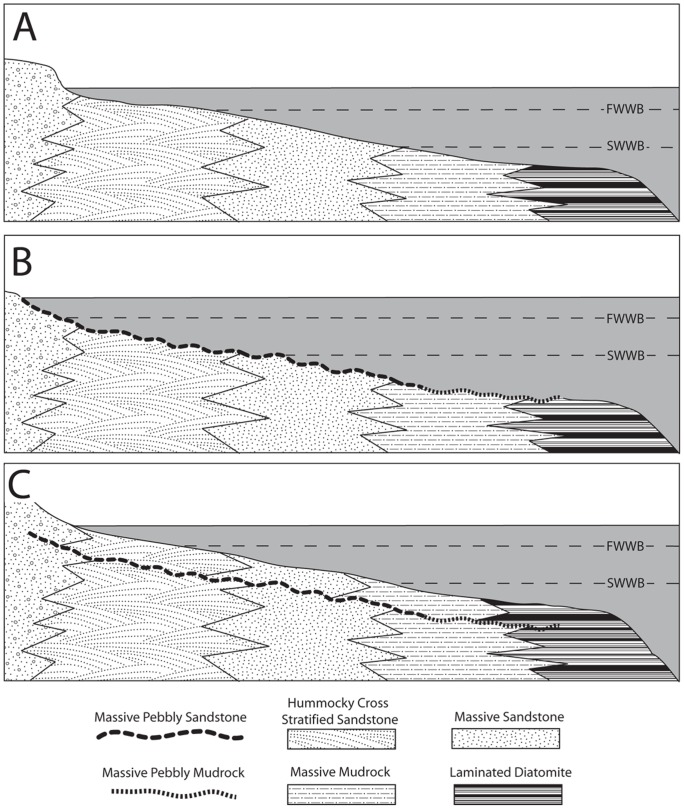
Depositional interpretation of lithofacies within the Purisima Formation. (A) lateral relationships of non-bonebed lithofacies, prior to bonebed formation. (B) Seafloor erosion during initial transgression; the massive pebbly sandstone (Spm) lithofacies is interpreted as representing proximal bonebed formation and the massive pebbly mudrock (Mpm) is interpreted as distal bonebed formation. (C) Deposition continues, resulting in a basinward shift in facies above the bonebed. Abbreviations: FWWB, fair-weather wave base; SWWB, storm-weather wave base.

This lithofacies does not interfinger with other lithofacies in the strict sense, but instead truncates underlying strata (Fig. 12). Abundance of phosphatic material and glauconite indicates association with the most extreme periods of non-deposition; truncation of underlying units and wide lateral extent also suggests association with large-scale erosion of the seafloor. The abundance of phosphatized bioclasts and phosphatic nodules also requires significant erosion during genesis of this lithofacies. The presence of *Ophiomorpha* burrows and *Gastrochaenolites* and *Trypanites* borings suggests this lithofacies corresponds with the *Skolithos* and *Trypanites* ichnofacies. The *Skolithos* ichnofacies characterizes non-hardground sandstone within this lithofacies, and is indicative of high-energy, shoreface and transition zone environments with a mobile substrate [79]. Conversely, the *Trypanites* ichnofacies is limited to hardgrounds (Bonebeds 5 and 6) and rockgrounds (Bonebed 1) and is indicative of high-energy settings with a fully lithified substrate [79].

The ***hummocky-cross stratified sandstone (Shc)*** lithofacies represents upper and lower shoreface deposition above storm and fair weather wave base (Fig. 12). Hummocky cross-stratification forms under conditions of combined oscillatory and unidirectional flow, with rapid suspension settling of sand [81], [82]. Combined flow may develop during hyperpycnal flow after heavy runoff produced by sediment-laden river plumes often associated with the effects of intense precipitation during on-shore storms [81]–[83]. Most commonly, as evidenced from modern shallow marine settings, sediment transport of sand and mud involves sediment disturbance by storm-wave resuspension and modification of resulting sediment gravity flows by geostrophic currents [84]–[86]. Storm deposition represents some of the highest energy depositional settings for the Purisima Formation [34]. In addition, rare *Ophiomorpha* is indicative of the high-energy, sandy substrate conditions of the *Skolithos* ichnofacies [79]. Frequent reworking of sediment is indicated by truncation and amalgamation of many beds in this lithofacies, and sparse evidence of bioturbation. Laterally extensive hummocky cross-stratified beds with bioturbated tops represent hyperpycnal deposition below fair weather wave base and closer to storm weather wave base, where fewer storms disturb the seafloor and longer periods of inter-storm bioturbation are able to occur [34]. In contrast, non-bioturbated hummocky-cross stratified sandstone beds are interpreted to have been deposited closer to and above fair weather wave base. Abundant well-preserved basal erosional surfaces (mantled with invertebrate bioclasts, mud rip-up clasts, and phosphate nodules) indicate frequent storm-related erosional events. However, the fewer terrigenous clasts and less taphonomically mature invertebrate fossils [34] suggest that although frequency of reworking is much higher than in the massive pebbly sandstone (Spm) lithofacies (i.e. timing between the formation of different bonebeds), the duration of nondeposition is temporally much shorter.

The ***massive sandstone (Sm)*** lithofacies represents deposition below storm weather wave base in the shoreface-offshore transition zone (Fig. 12). The massive and monotonous nature of this lithofacies is due to pervasive bioturbation. Because of the greater water depth in this depositional setting, less frequent storm-induced modification of the substrate failed to erase the bioturbatory overprint [34]. Although primary sedimentary structures are lacking, abundance of laterally extensive shell beds and pavements suggest this lithofacies represents storm-deposited beds extensively overprinted and rendered structureless by bioturbation. Sharp scours at the base of rare hummocky cross-stratified beds indicate erosion and reworking of the sediment substrate prior to deposition. The presence of sand below storm weather wave base also suggests sediment introduction by infrequent storm-related event deposition followed by extensive bioturbation during fair-weather periods [87]. Ichnotaxa including *Ophiomorpha* and *Planolites* suggest that this lithofacies corresponds to both the *Skolithos* and *Cruziana* ichnofacies [79]. Because the *Cruziana* ichnofacies is typical of slightly deeper water than the *Skolithos* ichnofacies [79], presence of both suggests deposition in the shoreface to offshore transition zone. Interfingering of the Sm lithofacies with the hummocky cross-stratified sandstone (Shc) of more proximal high-energy settings and massive mudrock (Mm) of offshore quiet-bottom settings supports this interpretation [34].

### Mudrock Lithofacies

#### Description

The ***massive pebbly mudrock (Mpm)*** lithofacies is similar to the massive pebbly sandstone (Spm) lithofacies, and most commonly found in Bonebed 2 and other poorly exposed (unnumbered) bonebeds in Section 1. Interstitial matrix is massive, pervasively bioturbated mud; the coarse fraction consists of very poorly-sorted, matrix-supported pebbles and cobbles with rare terrigenous clasts and phosphatic nodules (Fig. 10B). Small zones may be conglomeratic and clast-supported. Phosphatic nodules are internally homogenous and lack mollusk or crustacean skeletal elements. Vertebrate skeletal elements are relatively abundant, with no invertebrate body fossils present. Burrows including *Ophiomorpha* and *Thalassinoides* (horizontal branching tube-shaped burrows) are typically infilled with bonebed debris. Other trace fossils include *Teichichnus* and *Planolites* (horizontal tube-shaped burrows <2 cm in diameter). Vertebrate skeletal elements are often fragmented and heavily phosphatized (Stage 2A). This lithofacies interfingers with the massive mudrock (Mm) and laminated diatomite lithofacies (Mld) (Fig. 11D).

The ***massive mudrock (Mm)*** lithofacies primarily includes siltstone, with lesser amounts of mudstone and diatomaceous lithologies. This facies appears to lack any obvious internal erosional surfaces, and exhibits a tabular geometry (Fig. 10F). Planar laminated siltstone occasionally forms couplets with massive siltstone. Some parts of this facies include thin horizons of ripple cross-laminated siltstone. Other parts exhibit stacked beds (∼1 meter thick) of very fine sandstone with occasional shell lags at their base that fine upward into siltstone and mudstone. This lithofacies harbors a variety of trace fossils [63], including *Teichichnus, Planolites*, and rare *Thalassinoides* and *Ophiomorpha*. The ichnofabric typically consists of cross-cutting traces; small trace fossils and burrows (<1 cm wide) are preserved within this lithofacies. *Ophiomorpha* is occasionally infilled with sand if close to overlying sandstone. Articulated bivalves (*Tresus*, *Anadara*) occur as monotaxic clumps or in isolation; partial colonies of *Crepidula* are also present. This lithofacies interfingers with the laminated diatomite (Mdl) and massive sandstone (Sm) lithofacies (Fig. 11B–D).

The ***laminated diatomite (Mld)*** lithofacies occurs only in the lowermost part of the Santa Cruz section. It consists of finely laminated gray-yellow diatomite with a tabular geometry (Fig. 10D). Few trace fossils (<1 cm wide) occur in this lithofacies. This facies is sparsely fossiliferous and usually lacks calcareous skeletal material. One horizon in particular (4 meters above the base of the Purisima Formation) exhibits a sharp contact with underlying massive diatomite below, and is mantled by sand, woody debris, and rare vertebrate elements and fragmentary mollusks. This is the same stratigraphic position and locality of a fragmentary ‘whale fall’ assemblage discovered by one of us (F.A. Perry) in 1993. This facies interfingers with the massive mudrock (Mm) lithofacies (Fig. 11D).

#### Interpretation

The ***massive pebbly mudrock (Mpm)*** lithofacies likely formed in a manner similar to the massive pebbly sandstone (Spm) lithofacies. The abundance of phosphatic nodules indicates a substantial decrease in sedimentation rate. Additionally, because phosphate nodules only form below the sediment-water interface [75], their abundance indicates erosion of the substrate during a long depositional hiatus. Lack of calcareous material may be due to the low pH settings associated with phosphogenesis [75], [88], although calcareous macrofossils are generally absent from the diatomaceous portions of the massive mudrock (Mm) lithofacies that brackets (above and below) the only known exposures of the massive pebbly mudrock (Mpm). Finer-grained sediment (massive siltstone and diatomite) in this lithofacies suggests it may record development of distal bonebeds (or distal portions of a bonebed) in offshore environments. Although *Ophiomorpha* and *Thalassinoides* of the *Skolithos* ichnofacies are typical of sandy, high energy environments [79], their occurrence here is likely due to the high energy associated with bonebed formation. Other observed traces such as *Teichichnus* and *Planolites* (*Cruziana* ichnofacies) are more typical of lower energy, muddy environments [79]. As the massive pebbly sandstone (Spm) likely records bonebed formation within both shoreface and transition zone settings; given the large lateral extent of bonebeds in the Purisima Formation (see **6.** **Bonebeds**), a single bonebed may extend across the shelf from areas of nearshore massive pebbly sandstone (Spm) deposition to deeper offshore settings where massive pebbly mudrock (Mpm) accumulated (Fig. 12).

The ***massive mudrock (Mm)*** lithofacies represents offshore deposition well below storm weather wave base and beyond the limits of sand delivery to the shoreface-offshore transition zone (Fig. 12). Deposition here largely takes place by suspension fallout of silt and clay under low energy conditions. Stacked upward-fining beds with occasional shell lags [63] most likely represent rare distal storm-generated traction transport events. The massive nature of the sediment is again due to pervasive bioturbation. Biogenic activity was relatively unaffected by tractive current disturbance of the substrate, which is also reflected by an abundance of mollusk concentrations preserved in life position [34]. At other Purisima Formation localities, this lithofacies interfingers with turbidites, indicating deposition on the outer shelf near the shelf-slope break [34] that is corroborated by bathyal foraminifera [89]. Abundant trace fossils of the *Cruziana* ichnofacies suggest deposition in muddy, low energy offshore shelf environments [79].

The ***laminated diatomite (Mld)*** lithofacies is present in only a single section, representing the last pulse of “Monterey Formation-type” deposition in Northern California [90], and marking a brief return to the biosiliceous sedimentation that characterized the underlying Santa Cruz Mudstone and Monterey Formation. A combination of high productivity and formation of isolated, sediment starved basins has been implicated in the richly diatomaceous deposits of the Monterey Formation [65], conditions that likely persisted during deposition of the lowermost Purisima Formation. Absence of trace fossils and invertebrate body fossils from this lithofacies suggests anoxic or dysoxic pore and bottom water. This lithofacies was deposited by a biogenic rain of diatom tests in offshore settings at or near the shelf-slope break well below storm weather wave base [35] (Fig. 12).

### Lithofacies Associations

#### Shoreface lithofacies association

The hummocky cross-stratified (Shc), massive (Sm), and massive pebbly sandstone (Spm) lithofacies commonly occur together, and interfinger more frequently with each other than with finer-grained lithofacies (Fig. 11). This suite of sandstone lithofacies represents deposition ranging from slightly below storm weather wave base to above fair weather wave base on the proximal portion of the continental shelf near the shoreline (Fig. 12). Relative to bottom energy conditions, deposition spanned the middle-lower shoreface to offshore transition zones [91], [92]. Abundant internal truncations and shell beds indicate frequent, high energy disturbance at the sediment-water interface by storm activity and fair weather wave activity [93], [94]. The majority of bonebeds within this lithofacies association have internal erosional surfaces, also indicating relatively higher energy than the offshore lithofacies association. Preservation of primary sedimentary structures in some strata indicate higher sedimentation rates and more frequent sediment transport in the shallower nearshore (shoreface) settings than in lithofacies of the more distal offshore shelf environment.

#### Offshore lithofacies association

The massive mudrock (Mm), laminated diatomite (Mld), and massive pebbly mudrock (Mpm) lithofacies occur together, and more frequently interfinger with each other than with any of the coarser sandstone lithofacies (Fig. 10). This suite of mudrock lithofacies represents deposition entirely below storm weather wave base, and in distal, offshore parts of the continental shelf (Fig. 12). Pervasive bioturbation, *in situ* phosphate nodules, and (potentially) hiatal bonebeds are all indicative of relatively low sedimentation rates. The fine-grained nature of this lithofacies association is due to suspension fallout of mud and diatom tests offshore at distances far from the reach of fair-weather nearshore (shoreface) sediment transport or storm-generated combined flow delivery of sediment [95]. Additionally, thick sections of diatomite indicate certain areas of the outer shelf were starved of siliciclastic sediment, permitting biogenic sediment to accrue. The laminated nature of some diatomaceous strata indicates anoxic conditions restricted the bioturbating infauna, further suggesting deposition in sediment-starved environments of the outer shelf.

## Depositional and Stratigraphic Framework

The vertical distribution of lithofacies within the Santa Cruz section of the Purisima Formation allows interpretation of its depositional history in the context of successive depositional environments. Four contiguous exposures of the Santa Cruz section of the Purisima Formation exist (Fig. 1C). The uppermost section predominantly represents upper shoreface, nearshore, foreshore, and estuarine depositional settings [34], and due to its lack of vertebrate fossils, was not included in this study. The other three sections are referred to herein as section 1, section 2, and section 3 (Figs. 2, 4).

Section 1 (4A) is nearly 50 meters thick, and is located southwest of the city of Santa Cruz, CA. Section 1 includes the basal erosional unconformity of the Purisima Formation, which is mantled by massive glauconitic sandstone and bonebed debris (Bonebed 1). This grades upwards into massive diatomite that includes another bonebed (Bonebed 2), which is in turn overlain by laminated diatomite. Bioturbated diatomite overlies the laminated diatomite, and the rest of section 1 records a gradual increase in grain size from bioturbated diatomite to massive siltstone, and a 25 meters thick, monotonous section of massive sandstone. Two as-yet unstudied bonebeds occur within massively bedded diatomite and sandstone above Bonebed 2 in this section.

Section 2 (4B) is approximately 30 meters thick, and occurs between Santa Cruz and Capitola, CA. It includes a 10 m-thick monotonous section of massive sandstone at its base, although several outcrops are separated (by incised stream valleys) and it is unclear how many meters of section are missing. This is overlain by massive siltstone grading upward into massive sandstone overlain by hummocky cross-stratified sandstone, and topped with a thin bonebed (Bonebed 3) overlain by massive siltstone. This siltstone is overlain by another bonebed (Bonebed 4). The overlying massive siltstone above the bonebed includes several 0.5–1.0 m-thick fining-upward beds with very fine sand and occasional shell lags at the base, with sand and mollusk concentration increasing upward.

Section 3 (Fig. 4C) is approximately 30 meters thick, and occurs in the vicinity of Capitola, CA. This section exhibits a basal hummocky cross-stratified sandstone that is truncated by Bonebed 5, which in turn is overlain by massive sandstone grading upward into massive siltstone. This in turn grades back into massive sandstone that becomes increasingly fossiliferous upsection. This massive sandstone grades into hummocky cross-bedded sandstone showing progressive decrease in bioturbation and increase in the thickness of mollusk fossil concentrations at the base. This in turn is capped by Bonebed 6, which is overlain by massive sandstone.

Although only a single vertical section exists, the depositional history of the Santa Cruz section of the Purisima Formation can be explained within a sequence stratigraphic context, as discontinuity bounded units are evident within the Purisima Formation (Fig. 4). The base of section 1 represents a significant shallowing relative to the offshore depositional setting of the Santa Cruz Mudstone [66]. Because of the large basinward offset in depositional setting, this can be interpreted as a ‘forced regression’ [96]. Additionally, tectonic deformation of the Santa Cruz Mudstone prior to Purisima Formation deposition [66], in concert with the relative change in depositional setting, suggests that a ‘forced regression’ may have been caused by uplift of the basin floor prior to (or during) the depositional hiatus that formed Bonebed 1. Bonebed 1 is identified as a sequence boundary. The next 10 meters of section represents a gradual transition to deeper water sedimentation in the change from the massive sandstone to massive mudrock and eventually laminated diatomite lithofacies (Fig. 4A). Because this section represents a gradual transgression overlying a sequence boundary, it is identified as a thin Transgressive Systems Tract (TST). Bonebed 2, present three meters above the base, may represent a distal portion of a transgressive surface of erosion; due to the uncertainty of this feature, the section between Bonebed 1 and 2 is not identified as a Lowstand Systems Tract (LST), and instead assigned to the TST. For example, although the LST in the sequence stratigraphic model of Van Wagoner et al. [96] is bounded below by the sequence boundary and transgressive surface of erosion above, the transgressive surface of erosion may in fact be telescoped with the sequence boundary [2]. Thus, Bonebed 1 may include both the sequence boundary and transgressive surface of erosion, and perhaps the LST is not preserved within the Purisima Formation. The rest of section 1 is difficult to subdivide, but represents gradual shallowing. The unnumbered bonebeds in the upper part of section 1 may represent marine flooding surfaces at the base of parasequences (which typifies sections 2 and 3). Altogether, above the TST, the rest of the Purisima Formation represents a stacked series of shallowing-upward parasequences with bonebeds at their basal marine flooding surfaces, and can all be identified as the Highstand Systems Tract (HST; Fig. 4).

Section 2 includes at least three parasequences, two of which include marine vertebrate concentrations at their basal parasequence boundaries. Parasequence boundaries are also termed Marine Flooding Surfaces (MFS), and represent shoreward offsets in facies [96]. Section 3 preserves the best example of a parasequence, and is capped by Bonebed 6. Overall, the parasequences within the HST represent successively shallower environments. As previously mentioned, the uppermost section of the Purisima Formation represents nearshore, shoreface, and estuarine environments, and may still represent part of the HST (Fig. 4), as terrestrial Pleistocene Aromas Sands appear to conformably overlie the Purisima Formation [35]. Admittedly, this poorly exposed section is not well-studied.

In summary, the Purisima Formation represents an initial shallowing, after uplift, deformation, and lithification of the underlying Santa Cruz Mudstone, followed by a short transgression (TST). This was followed by deposition of packages of rock showing basinward shifts in facies (parasequences) bounded by discontinuities representing slight shoreward facies offsets (parasequence boundaries/marine flooding surfaces/bonebeds). These parasequences (highstand systems tract) record increasingly shallower facies, eventually grading into terrestrial deposits.

## Bonebeds

Six bonebeds from the Santa Cruz section of the Purisima Formation were studied in detail (Table 4). Several other bonebeds were observed – two in the middle of section 1, and a third within section 3, several meters above Bonebed 5. Bonebeds 1, 4, 5, and 6 are exposures of the massive pebbly sandstone (Spm) lithofacies, whereas Bonebed 2 is an exposure of the massive pebbly mudrock (Mpm) lithofacies, and Bonebed 3 an exposure of the massive sandstone (Sm) lithofacies. These bonebeds occur in sections 1, 2, and 3 (Fig. 4).

**Table 4:** Comparison of bonebed characteristics based on field observations.

Bonebed Characteristics	Bonebed 1	Bonebed 2	Bonebed 3	Bonebed 4	Bonebed 5	Bonebed 6
Lateral Extent	700 m	500 m	2400 m	2000 m	50 m	2500 m
Upper Contact of α-interval	Sharp	Gradational	Gradational	Gradational	Sharp	Sharp
Overlying deposits	Transition Zone	Offshore	Offshore	Offshore	Shoreface	Shoreface
Underlying deposits	Offshore	Offshore	Shoreface	Offshore	Transition Zone	Transition Zone
Lithofacies	Massive Pebbly Sandstone	Massive Pebbly Mudrock	Massive Pebbly Sandstone	Massive Pebbly Sandstone	Massive Pebbly Sandstone	Massive Pebbly Sandstone
Vertebrate Sample Size	107	56	51	441	55	206
Mollusk Bioclasts	Absent	Absent	Abundant	Abundant	Rare	Abundant
Phosphatic Clasts	Absent	Abundant	Rare	Abundant	Abundant	Abundant

### Bonebed 1

#### Description

**Figure 13:**
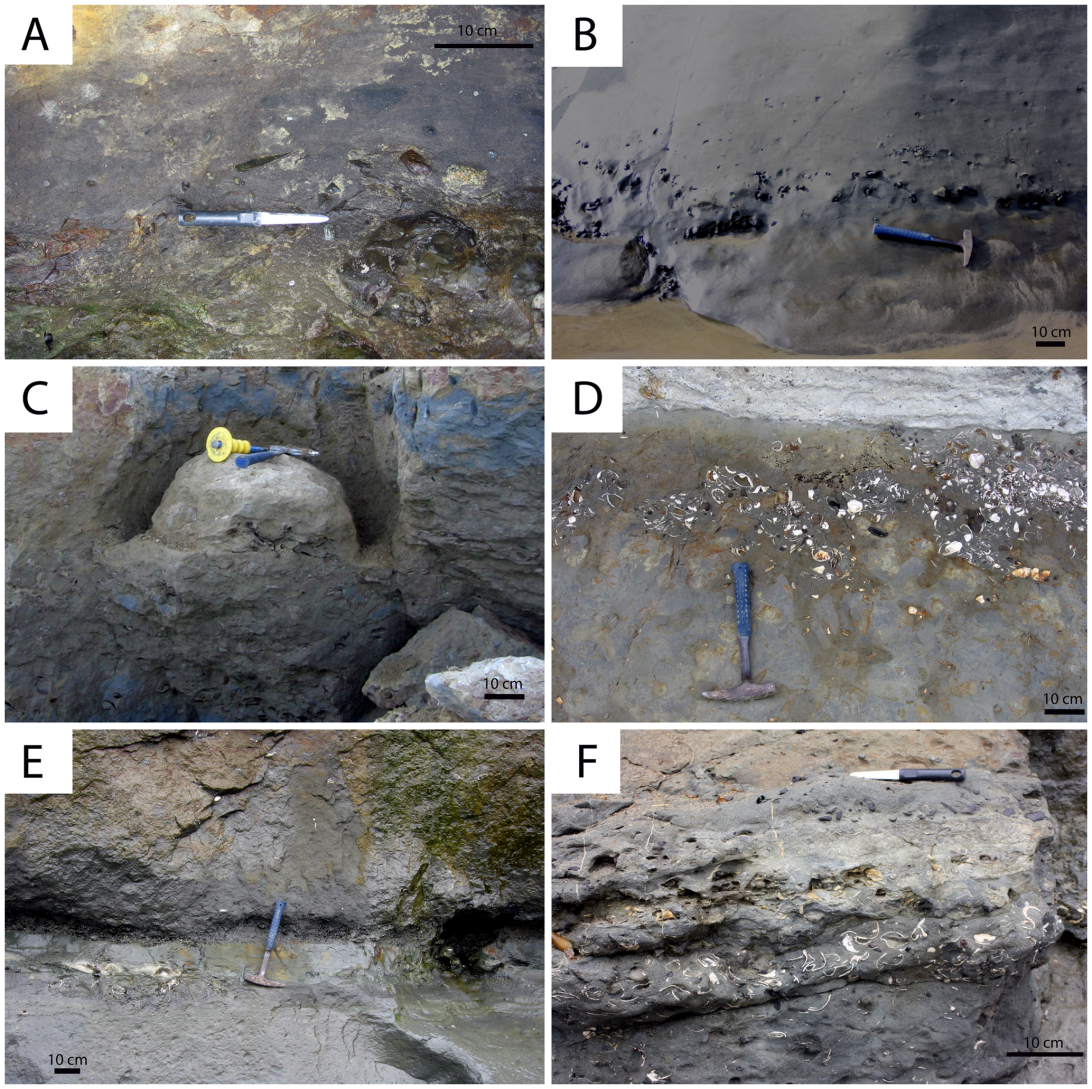
Bonebeds of the Purisima Formation examined in this study. Studied bonebeds include (A) Bonebed 1, (B), Bonebed 2, (C), Bonebed 3, (D), Bonebed 4, (E), Bonebed 5, and (F) Bonebed 6.

Bonebed 1 (UCMP locality V99875) is located above the unconformable contact between the Santa Cruz Mudstone and overlying Purisima Formation (Fig. 4, 13A, 14A). The Santa Cruz Mudstone below the contact consists of interbedded couplets of unconsolidated siltstone and silicified porcelanite. The lower contact of this bonebed is highly irregular, with 20 cm of relief; many burrows (*Ophiomorpha*) extend up to 2.5 meters below the contact and are infilled with glauconitic sandstone and coarse bonebed debris. The matrix lithology of the bonebed is primarily massive (and burrow-mottled) medium grained, glauconite-rich sandstone (Spm) with occasional granules. Coarse clasts are extraformational porcelanite pebbles and cobbles from the Santa Cruz Mudstone and extrabasinal terrigenous pebbles (and rare cobbles). Cobbles of the Santa Cruz Mudstone often exhibit flask-shaped clam borings identified as *Gastrochaenolites* (circular aperture with flask-shaped cross section, 1–3 cm long) on all sides, in addition to conchoidal fracturing of many surfaces. Bonebed 1 is an approximately 50 cm thick matrix-supported conglomerate. Coarse clasts and bioclasts are most densely concentrated (matrix supported or loosely packed) in the basal 20 cm thick β-interval, and are increasingly more dispersed within the overlying γ-interval. Cobbles and large bioclasts are almost always in the lower 20 cm and occasionally in contact with the truncated Santa Cruz Mudstone. Lithic gravel and bioclast size decreases upwards. The thickness of this generally tabular bonebed is maintained laterally, and it can be traced laterally along the shoreline for 0.7 km.

**Figure 14:**
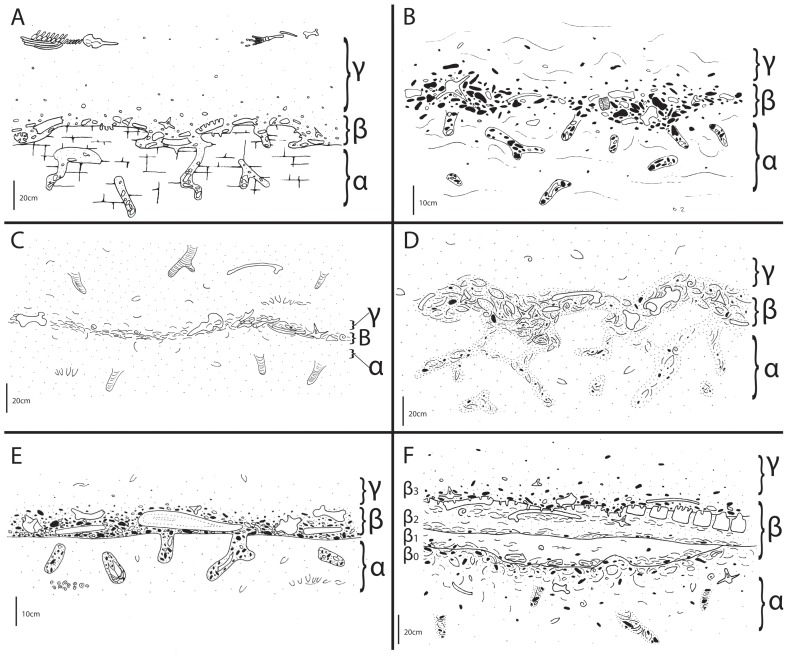
Diagrammatic illustration of Purisima Formation bonebeds examined in this study. Studied bonebeds include (A) Bonebed 1, (B), Bonebed 2, (C), Bonebed 3, (D), Bonebed 4, (E), Bonebed 5, and (F) Bonebed 6. See 3. Methods and Fig. 7 for explanation of architectural divisions. Subintervals β_0–3_ also indicated for Bonebed 6 (F).

#### Vertebrate preservation

Postcranial bones (complete and fragmented) of cetaceans, sirenians, and pinnipeds are common, with cetacean and sirenian ribs the most frequently encountered elements. Shark teeth and fish bones are less common, and mammal teeth and bird bones are rare. Most bones exhibit Stage 1 abrasion, and some bones exhibit Stage 2–3; few bones are unabraded (Stage 0). Most bones exhibit some fragmentation or fracturing. No bones or teeth exhibit any phosphatization. Most vertebrate skeletal elements are within 10–15 cm of the lower contact (within the β-interval), and most large bones are in contact with or in close proximity to the basal surface. Within the β-interval, no articulated remains occur. Associated remains are extremely rare in this interval (one pair of associated walrus tusks were found from this lower zone). Articulated and associated skeletons occasionally occur 30–50 cm above the base within the γ-interval, along with well-preserved (unabraded, unfragmented) isolated vertebrate skeletal elements ranging in size from small teeth and bone fragments (<1 cm wide) to complete mysticete ribs up to 1 meter long.

### Bonebed 2

#### Description

Bonebed 2 (UCMP locality V99877) is located 3 meters above the base of the Purisima Formation (Fig. 4, 13B, 14B) within a massively bedded, pervasively bioturbated and burrow-mottled diatomite. The matrix of this bonebed corresponds to the massive pebbly mudrock (Mpm) lithofacies. No visible change in lithology occurs within the bonebed or within a meter above or below. The bonebed is tabular with gradational upper and lower contacts. The majority of clasts and bioclasts are concentrated in the β-interval, and bioclast packing decreases above (γ-interval) and below (α-interval). Most large pebble- and cobble-sized clasts and large bioclasts occur in the β-interval; clast/bioclast size decreases upwards and downwards from the β-interval. The β-interval pinches and swells, and is generally patchy; clasts and bioclasts are typically floating. (loosely packed, but occasionally densely packed). The α- and γ-intervals are similar in their architecture and contain dispersed clasts and bioclasts that often occur as localized clumps or pods (including pebble-size clasts/bioclasts) oriented vertically to oblique (*sensu* [6]). Occasionally, these pods (*sensu* [6]) of bonebed debris fill *Ophiomorpha* burrows. These clast-bioclast pods are often densely packed and clast-supported; some pods occur up to 2.5 meters below the bonebed. One meter below Bonebed 2, there is a sharp, irregular contact between massive glauconitic sandstone below and massive diatomite above. This contact in some exposures is mantled by debris similar to that of Bonebed 2, in some places appearing as a thinner, discontinuous bonebed. Where exposed in plan view, bonebed debris at this horizon appears to be confined to horizontal connected burrows forming a polygonal pattern. Clasts are primarily phosphatic pebbles and cobbles in the 1–5 cm size range with rare terrigenous pebbles. Most phosphatic clasts are black, well-rounded nodules. Bonebed 2 can be traced laterally for 0.5 km.

#### Vertebrate preservation

Cetacean bones and bone fragments constitute the most abundant vertebrate element. Pinniped bones are common, while shark teeth, fish bones, and bird bones less common. Abrasion of these elements ranges from Stage 0–2, but most are unabraded (Stage 0). The majority of bones are preserved as fragments. Most bones appear phosphatized; many of these exhibit phosphatized interstitial matrix and adhering phosphatic nodules. The majority of vertebrate skeletal elements are concentrated within the β-interval, as are the larger elements. Bioclasts and clasts are loosely to densely packed within the β-interval, and increasingly more dispersed in the α- and γ-intervals. No articulated or associated specimens are recorded from this bonebed. Sizes of vertebrate bioclasts range from bone fragments and teeth less than 1 cm to partial cetacean bones up to 40 cm long.

### Bonebed 3

#### Description

This bonebed (UCMP locality V90042; Fig. 13C, 14C), located in the uppermost portion of Section 2 (Fig. 4), is a 5–15 cm thick, laterally extensive tabular shell-rich interval with occasional vertebrate skeletal elements. This concentration occurs within massively bedded, burrow mottled sandstone (Sm) lithofacies. The base of a 1 meter thick bed of large-scale hummocky-cross stratified sandstone (Shc) with an erosional scour at its base is present 1–1.5 meters below this stratum. This underlying bed becomes increasingly more bioturbated toward its top, transitioning into massively bedded sandstone. Convoluted bedding and occasional ball-and-pillow structures occur near the top of the non-bioturbated interval (50–70 cm below Bonebed 3). Upper and lower contacts of Bonebed 3 are gradational and demarcated by a gradual decrease in mollusk shells above and below the β-interval. The α- and γ-intervals are less than 10 cm thick. Mollusk shells are the most abundant coarse material within Bonebed 3; terrigenous clasts and phosphatic nodules are rare (always pebble sized), and vertebrate material is slightly less abundant than terrigenous clasts. Mollusk shells are loosely packed, consisting mostly of disarticulated bivalve shells generally oriented concordant and oblique to bedding and rarely nested. Bones, teeth, and pebbles always occur within the β-interval. Large elements (i.e. skeletons, skulls) extend above the β-interval into the γ-interval, but not below into the α-interval. Bonebed 3 extends laterally for 2.4 km.

#### Vertebrate preservation

Cetacean bones are the most common vertebrate elements. Shark teeth, bird bones, and pinniped bones are slightly less common. Vertebrate skeletal elements are sparse, usually isolated, and typically unabraded (Stage 0), or less commonly slightly abraded (Stage 1). No bones exhibit evidence of phosphatization. Vertebrate skeletal elements range in size from small teeth and gill rakers (<5 mm) to complete mysticete bones and skeletons over 1 meter long. A few articulated and associated mysticete skeletons are known from Bonebed 3.

### Bonebed 4

#### Description

Bonebed 4 (UCMP locality V6875; Fig. 13D, 14D), located near the top of Section 2 (Fig. 4), is a tabular unit (10–40 cm thick) containing abundant mollusk shells, large phosphate nodules, and well-preserved vertebrate skeletal material. It is underlain by massive siltstone (Mm) and its matrix comprises very fine to fine grained massive pebbly sandstone (Spm). Gravel-size clasts include abundant mollusk shells (bivalves, gastropods), crustacean skeletal elements, phosphatic nodules, terrigenous pebbles, marine mammal bones, bone fragments, rare bird bones, shark teeth, fish bones, and calcified cartilage. Phosphatic nodules often include steinkerns, external molds, and abundant cylindrical nodules with fecal pellets and partial *Callianassa* skeletons inside. Many phosphate nodules include original calcareous mollusk skeletal elements; some nodules are up to 15–25 cm wide and contain abundant densely packed mollusk shells.

Bonebed 4 maintains a relatively constant thickness over its lateral extent, but locally shows some thickness variation, and the β-interval pinches and swells from 10–40 cm in thickness. The lower contact is gradational, marking a transition from siltstone to sandstone (and a gradual increase in bioclast packing within the α-interval), and includes many *Ophiomorpha* burrows infilled with sandstone and bonebed debris, extending 1 meter below the β-interval. The upper contact is also gradational, marking a decrease in grain size and bioclast packing upwards within the γ-interval. Clast and bioclast packing is highest within the β-interval; coarse material is rare within the γ-interval. Clasts and bioclasts are less abundant in the α-interval, and increase in abundance towards the β-interval. The coarse material within the β-interval is mostly matrix supported (loosely packed), and there are localized areas of clast-support (dense packing). Bonebed 4 can be traced laterally for 2 km. To the northeast, Bonebed 4 transitions to a 20–25 cm thick bioclastic bed with horizontally oriented mollusks, and lacking much bioclastic material in the α- and γ-intervals. The β-interval is densely packed and exhibits a sharp planar base, although no sharp sedimentary contact exists.

#### Vertebrate preservation

Cetacean bones and bone fragments are the most common vertebrate skeletal elements. Pinniped bones and teeth, shark teeth, calcified elasmobranch cartilage, fish bones, and bird bones are less common. Bones typically exhibit Stage 0 abrasion with Stage 1–2 less common. Heavily phosphatized bones (Stage 2) are rare, but slightly phosphatized bones abundant (Stage 1) and many bones exhibit adhering phosphatic matrix (Stage 0B–C and Stage 1B–C). The largest vertebrate bones (pinniped and cetacean bones) occur within the β-interval. Vertebrate bones are never articulated, but some partial, disarticulated skeletons (comprising only a few bones; Disarticulation Stage 3) have been found in Bonebed 4. Vertebrate skeletal elements range from small shark and pinniped teeth and *Cetorhinus* gill rakers under 5 mm, to complete baleen whale bones up to 2 meters in length.

### Bonebed 5

#### Description

Bonebed 5 (UCMP locality V99866; Fig. 13E, 14E) is a clast-supported conglomerate located 2 meters above the base of Section 3 (Fig. 4) at a contact between interfingering massive siltstone (Mm) and hummocky cross-stratified sandstone (Shc) below and massive fine-grained sandstone (Sm) above. The underlying siltstone contains flat, tabular concretions 10–20 cm below the contact. The bonebed material mantles a sharp, irregular lower contact with 10–20 cm of local relief; laterally the bonebed instead mantles hummocky cross-stratified sandstone where it has eroded completely through the thin massive siltstone. Abundant wide borings identifiable as *Gastrochaenolites* (∼3–10 cm wide) extend 20–100 cm below the β-interval and are filled with bonebed debris. Rarely, the bottom of these borings house an *in situ* pholad clam nearly as wide as the structure; the boring and interior of the pholad clam are filled with bonebed debris. Bonebed matrix includes very poorly sorted fine to very coarse sandstone. Granule, pebble, and (rarely) cobble-size clasts comprise predominantly phosphatic nodules, steinkerns, and external molds of mollusks. Terrigenous clasts (mostly pebbles) are also abundant. Rare large tabular disc-shaped calcareous siltstone cobble-sized nodules (2–4 cm thick, up to 20 cm wide) occur within the β-interval. Some large phosphatic nodules include monospecific clusters of gastropods (*Nassarius*) and bivalves (*Anadara*) retaining original calcareous shell material; similar clusters occur in the underlying siltstone. Rare large mollusk-shell bearing calcareous sandstone cobble-size nodules occur as well. Fragments and disarticulated portions of crustacean skeletons (*Callianassa* and *Cancer* leg segments and chelae) are abundant, and phosphatic nodules frequently contain partial and articulated crustacean skeletons. Many of these nodules are cylindrical, 2–4 cm wide, up to 10 cm long, and include clusters of lozenge-shaped fecal pellets and occasionally pincers and partial skeletons of *Callianassa*. Clasts and bioclasts are largest and densely packed (clast supported) within the β-interval directly above the irregular lower surface. The α-interval lacks dispersed vertebrate elements or clasts, and bonebed debris only occurs within the burrows described above. The γ-interval is characterized by gradational upward decreases in clast/bioclast packing (matrix supported, or dispersed) and size (i.e. fining upwards) from that of the β-interval. Pebbles and cobbles occur most frequently within the β-interval, within 10–15 cm of the basal surface or in contact with it. Large pebbles and cobbles are concordantly (and occasionally obliquely) oriented. Some parts of the β-interval are cemented with calcium carbonate. The basal horizon in some places cross-cuts the calcareous siltstone nodule-bearing stratum. Bonebed 5 can only be traced laterally for 50 m.

#### Vertebrate preservation

Vertebrate skeletal elements are most commonly abraded bone pebbles. More complete bones typically include partial cetacean ribs and vertebrae. Teeth in Bonebed 5 typically exhibit Stage 1 abrasion with roots typically more abraded than crowns. Bones typically exhibit abrasion Stage 2–3, although unabraded or lightly abraded (Stages 0–1) bones are less common. Elongate vertebrate elements are frequently fragmented with fragments of larger bones common. The largest vertebrate bioclasts occur within the β-interval. No vertebrate elements are articulated or associated. Bones and teeth are typically heavily phosphatized (Stage 2A, 2C). Vertebrate bioclasts range in size from small elasmobranch teeth and fish bones (<5 mm) to medium sized bones and bone fragments (10–15 cm).

### Bonebed 6

#### Description

This bonebed (UCMP locality V99869; Fig. 13F, 14F) is a phosphate pebble and bone rich cemented hardground located near the top of section 3 (Fig. 4). Bonebed 6 is the most sedimentologically, diagenetically, and taphonomically complex bonebed in the Purisima Formation. It marks a change from hummocky cross-stratified sandstone (Shc) lithofacies below to massive sandstone (Sm) lithofacies above. Bonebed 6 includes three sharp erosional surfaces; the cemented portion of this bonebed was subdivided into four units, Layers A–D, by Friede [88]. Field examination of this bonebed recognized the same units, although while Friede [88] used diagenetic boundaries (i.e. the margins of the cemented zone), this study recognizes four units based on surfaces preserved within. All three surfaces are preserved within the β-interval, and the β-interval includes Friede’s [88] Layer B and Layer C, the top of Layer A, and the bottom of Layer D. Instead, the three surfaces divide the β-interval into four subintervals, termed subintervals β_0_, β_1_, β_2_, and β_3_ (Fig. 14F) The cemented portion of Bonebed 6 is generally 20–30 cm thick, and localized concretionary tongues may protrude up to 30 cm above or below. These tongues typically form above or below baleen whale skulls or skeletons preserved in subinterval B_2_. Bonebed debris extends for 30–70 cm above the γ-interval, and 50 cm below. Within the α-interval and 1–2 meters below Bonebed 6, abundant *Ophiomorpha* burrows within hummocky cross-stratified sandstone and other burrows infilled with bonebed debris are present. In total, the bonebed (intervals α-γ) is roughly 2 meters thick.

The matrix of Bonebed 6 is a fine to medium grained sandstone with abundant glauconite grains. Phosphatic pebbles are the most abundant coarse clasts, while cobble-size phosphate nodules are less common; the non-bioclastic component of Bonebed 6 corresponds to the massive pebbly sandstone (Spm) lithofacies. Terrigenous clasts are rare. Bones, bone fragments and pebbles, vertebrate teeth, and calcified cartilage are less common than phosphate clasts. While calcareous mollusk shells are abundant in subintervals β_0_–β_1_, calcareous skeletal material occurs only rarely within subinterval β_2_ and is not present in subinterval β_3_. In subinterval β_0_, mollusk shells often exhibit phosphatized internal and external molds, whereas in subintervals β_2_ and β_3_, many phosphate clasts are steinkerns and external molds. Some nodules include molds of disarticulated, imbricated, and nested bivalve shells. Rare phosphatic nodules exhibit flask-shaped endolithic bivalve borings (*Gastrochaenolites*) and narrower, subcylindrical borings (*Trypanites*). Within 50–70 cm of the top of the α-interval, hummocky cross-stratified sandstone gives way to massively bedded sandstone exhibiting extensive burrow mottling. The sandstone within and above Bonebed 6 is massively bedded and pervasively bioturbated.

The α-interval includes hummocky-cross stratified sandstone with bonebed debris-infilled *Ophiomorpha* burrows at its base that transitions to massively bedded, bioturbated, matrix-supported bonebed conglomerate at the top. Bonebed debris (mollusk shells, phosphate pebbles, and crustacean and vertebrate skeletal elements) increases in abundance in the upper 10–20 cm (which marks the base of the β-interval). Subinterval β_0_ is truncated by a generally planar (but wavy on the centimeter scale) erosional surface.

Subinterval β_1_ is a 3–5 cm thick, medium-grained bioclastic sandstone that includes abundant densely packed, imbricated (and often nested) mollusk shells. Phosphatic material (nodules, crustacean and vertebrate skeletal elements) is rare. Subinterval β_1_ occurs in lenses, and is in turn truncated by an erosional surface that in many places completely truncates subinterval β_1_ down into subinterval β_0_.

Subinterval β_2_ is 10–20 cm thick bioclastic sandstone containing abundant densely-loosely packed mollusk shells. Vertebrate remains are well preserved, and include articulated (and associated) cetacean skeletons. Molluscan shells decrease in abundance towards the top, and mantle the lower erosional surface. Calcareous material can locally be rare or non-existent. Phosphatic nodules are slightly more common than in subinterval β_1_. This unit is also truncated by a sharp erosional surface (the lower contact of subinterval β_3_), with 20–30 cm of relief. Subinterval β_2_ is typically about 10 cm thick, but in some cases large bones protrude more than 10 cm above the base of subinterval β_3_. Abundant endolithic bivalve borings (*Gastrochaenolites*; 1–3 cm deep) extend down into this surface. This surface is phosphatized as a phosphatic rind or crust.

Subinterval β_3_ is a 50–70 cm thick massively bedded sandstone with abundant phosphatic material. Calcareous material is absent. The majority of clasts and bioclasts are in the pebble size range. Bonebed debris (phosphate nodules, bone fragments, teeth) mantles the lower surface, and decreases in abundance/packing upwards through the γ-interval. Bonebed 6 can be traced laterally for 1.1 km; blocks of this bonebed occur as boulders in Pleistocene terrace deposits 2.5 km further to the southwest, suggesting nearly 4 km of exposure.

#### Vertebrate preservation

Vertebrate skeletal material is abundant within subintervals β_0_, β_2_, and β_3_. Marine mammal bones, bone fragments, and bone pebbles are the most common vertebrate elements. Bones and teeth frequently exhibit abrasion Stage 1–2, commonly occurring as bone pebbles. Many bones are fragmented, and bone shards are common. Most bones in subintervals β_0_ and β_3_ are completely blackened and phosphatized, occasionally with adhering phosphatic matrix (Stage 2A–C); bones in subinterval β_2_ are occasionally phosphatized (Stage 1–2A) and bone surfaces near the upper erosional surface show a phosphatized interval (Stage 1A). Abundant articulated and associated skeletons (Articulation stages 0–3) occur within subinterval β_2_. Vertebrate skeletal elements in subintervals β_0_ and β_3_ are typically less than 10 cm in greatest dimension (either as nearly complete elements or fragments thereof). They range in size from small (<5 mm) shark, pinniped, and cetacean teeth and *Cetorhinus* gill rakers to individual bones (mysticete skulls, mandibles) up to 2.5 meters long.

## Comparative Taphonomy

### Comparisons within Lithofacies

#### Abrasion

**Figure 15:**
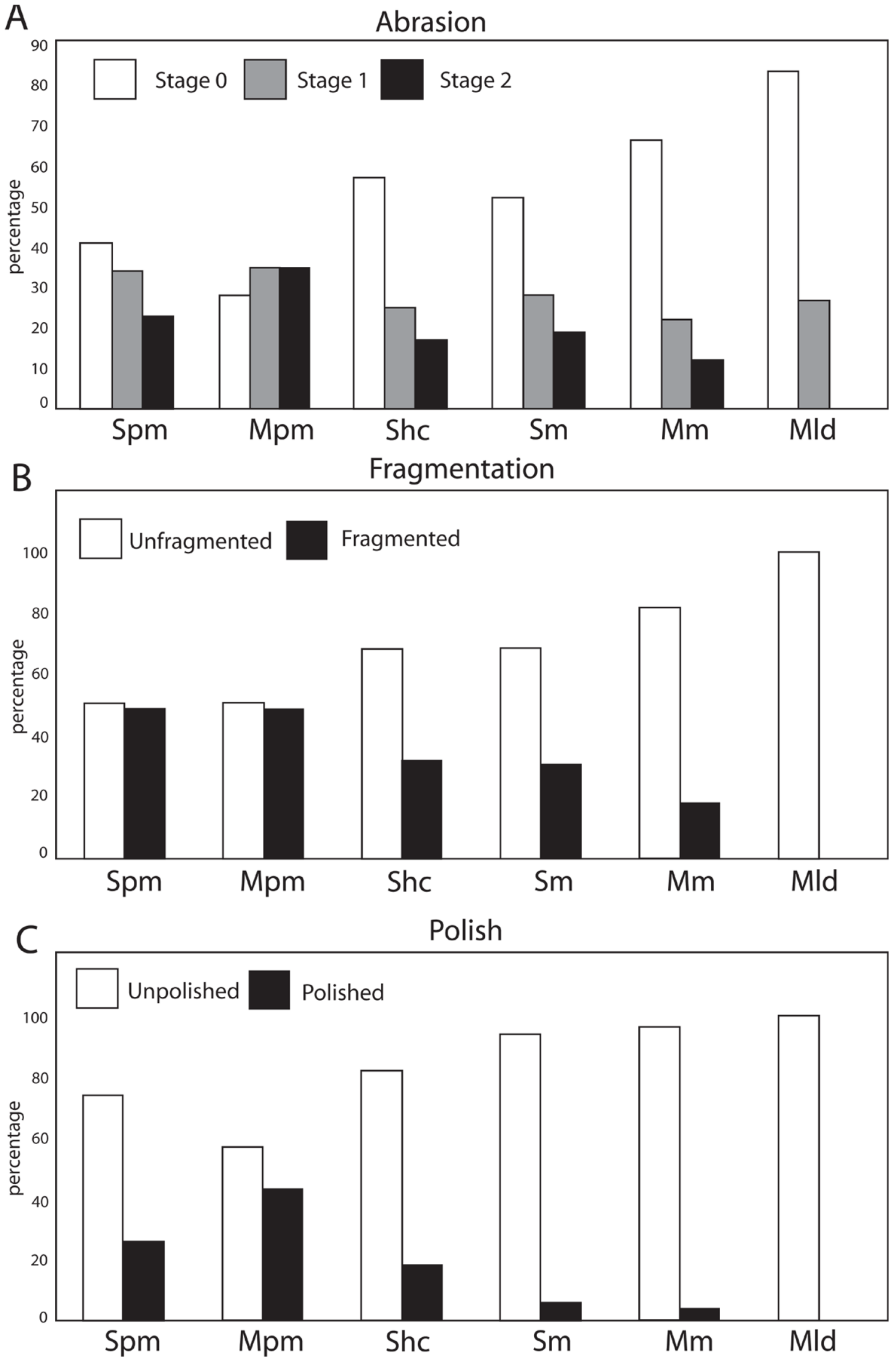
Histograms showing frequency of bone modifications (displayed as percentage of sample) in different lithofacies. Bone modifications include (A) abrasion, (B) fragmentation, and (C) polish.

Abrasion is most extensively developed within the massive pebbly sandstone (Spm; 58.2%, Stage 1–2) and mudrock (Mpm; 71.4%, Stage 1–2) lithofacies; the massive mudrock (Mm; 34.0%, Stage 1–2) and laminated diatomite (Mld; 16.6%, Stage 1) exhibit the least abraded skeletal elements, while the hummocky cross-stratified sandstone (Shc; 42.8%, Stage 1–2) and massive sandstone (Sm; 47.6%, Stage 1–2) samples are intermediate between the two extremes (Fig. 15A). This correlates well with bonebed-related lithofacies forming under conditions of extensive erosion during depositional hiatus, and documents an offshore decrease in abrasion amongst the non-bonebed lithofacies.

#### Articulation

**Figure 16:**
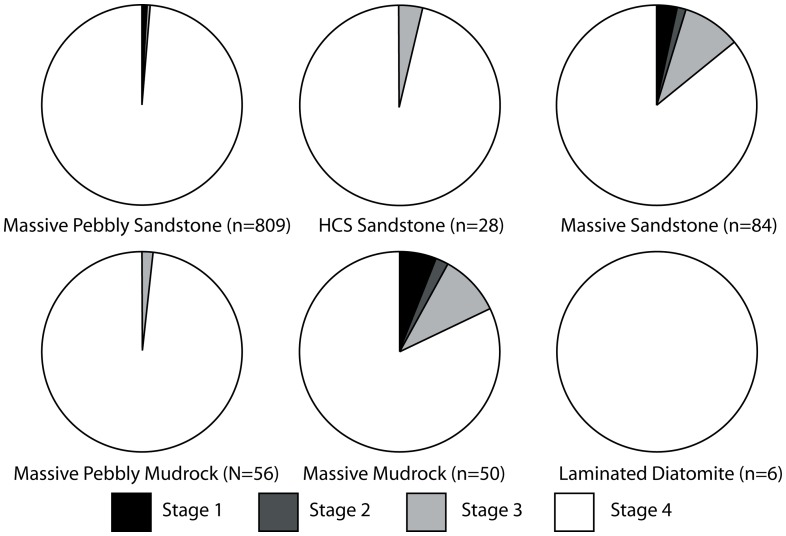
Pie charts showing articulation stage representation in each lithofacies.

Articulated and associated remains are generally rare in all lithofacies (Fig. 16). Articulation is virtually absent in the massive pebbly sandstone (Spm; 98.6% Stage 4), massive pebbly mudrock (Mpm; 98.2% Stage 4), hummocky cross-stratified sandstone (Shc; 96.4% Stage 4), and laminated diatomite (Mdl; 100% Stage 4). However, in the massive sandstone (Sm) and massive mudrock (Mm) lithofacies, articulated and associated elements (articulation stage 1–3) are more common (14.3 and 18.0%, Sm and Mm respectively; stages 1–3). Lack of articulation in laminated diatomite may reflect a sampling artifact, given the low sample size from this lithofacies (n = 6; Table 2); this lithofacies is broadly similar to laminated mudrocks that have produced Mesozoic marine vertebrate lagerstätten (e.g., [97]), and would be predicted to exhibit the highest frequency of articulation. Future fossil discoveries are necessary to test this prediction.

#### Fragmentation

Fragmentation follows a pattern similar to abrasion (Fig. 15B). The most abundantly fragmented vertebrate samples occur in the massive pebbly sandstone (Spm) and mudrock (Mpm) lithofacies (48.2% and 48.2%, respectively). Vertebrates are not fragmented within the laminated diatomite (Mdl), while the massive mudrock (Mm) exhibits a low percentage of fragmented remains (18%); slightly higher percentages of fragmented elements characterize the hummocky cross-stratified sandstone (Shc) and massive sandstone (Sm; 32.1% and 30.9%, respectively). Generally, the degree of fragmentation decreases offshore (Fig. 15B).

#### Polish

Polish is most abundant within the massive pebbly mudrock (Mpm; 42.8%), absent within the laminated diatomite (Mdl; 0%), and low within the massive sandstone (Sm; 5.9%) and mudrock (Mm; 4%) lithofacies (Fig. 15C). The hummocky cross-stratified sandstone (Shc) and massive pebbly sandstone (Spm) exhibit intermediate percentages (17.8% and 25.7%, respectively) of polished elements. Polish parallels abrasion and fragmentation in terms of abundance by lithofacies (Fig. 15C).

#### Phosphatization

**Figure 17:**
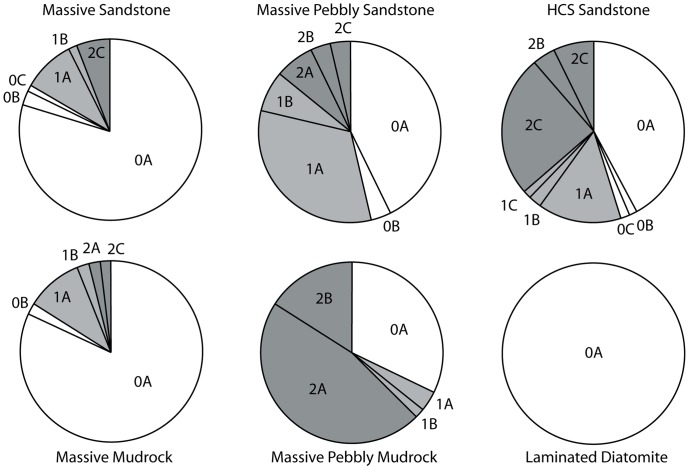
Pie charts showing phosphatization stage representation in each lithofacies.

Phosphatization shows a slightly different trend than other taphonomic modifications and is rare in fossils from the massive sandstone (Sm) and massive mudrock (Mm) lithofacies, and absent in the laminated diatomite (Mld) lithofacies. Phosphatized elements are common in the massive pebbly sandstone (Spm), massive pebbly mudrock (Mpm), and hummocky cross-stratified sandstone (Shc; Fig. 17). Furthermore, stage 2 phosphatization is less common in the massive pebbly sandstone (Spm) than in the hummocky cross-stratified sandstone (Shc) or massive pebbly mudrock (Mpm). In contrast, phosphatized vertebrate fossils of the massive pebbly mudrock lithofacies (Mpm) are nearly all stage 2A and 2B. The proportion of stage 1 and 2 phosphatization is similar in the massive sandstone (Sm) and mudrock (Mm) lithofacies. Large phosphatic (Stage XC) nodules are absent from the massive pebbly mudrock (Mpm), although present in both the hummocky cross-stratified sandstone (Shc) and massive pebbly sandstone (Spm) lithofacies (Fig. 17). Phosphatization thus appears to be correlated with lithofacies where erosion is implicit in its mode of formation.

### Taxonomic Comparisons

#### Abrasion

**Figure 18:**
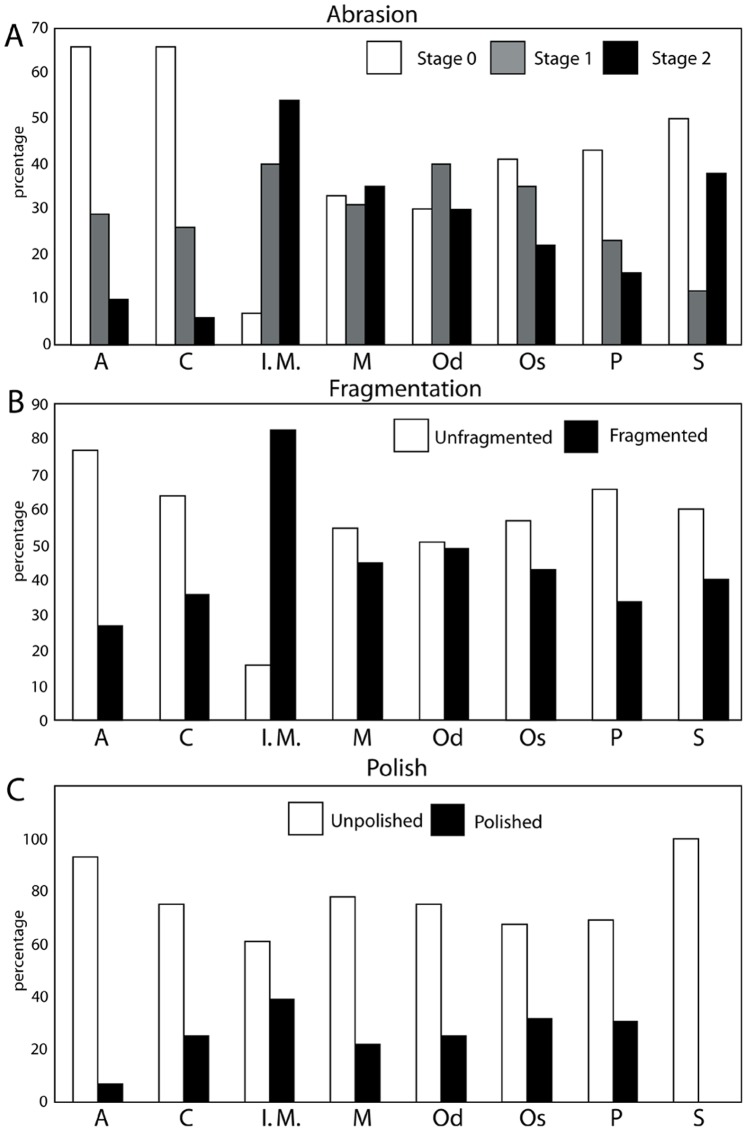
Histograms showing frequency of bone modifications (displayed as percentage of sample) in different marine vertebrate taxa. (A) abrasion, (B) fragmentation, and (C) polish. Abbreviations: A, Aves; C, Chondrichthyes; I.M., indeterminate mammal; M, Mysticeti; Od, Odontoceti; Os, Osteichthyes; P, Pinnipedia; S, Sirenia.

With the exception of indeterminate mammal bones, cetacean skeletal remains possess the highest degrees of abrasion (68.5% Stage 1–2, Mysticeti and Odontoceti combined). Abraded mammal bone pebbles are abundant (93.4% Stage 1–2), and the majority of these, although too incomplete to identify, are probably cetacean in origin based upon size and histology. Elasmobranch and bird elements are the least affected by abrasion (33.2 and 33.8% respectively, Stage 1–2), whereas pinniped and bony fish elements exhibit an intermediate (53.6% and 57.2% respectively, Stage 1–2) frequency of abrasion (Fig. 18A). This difference is probably due to the relatively robust nature of shark teeth, while cetacean bones are osteoporotic. In the case of diagnostic cetacean cranial elements, these may be abraded into bone pebbles past the point of identification, and smaller odontocetes are predicted to be more susceptible to taphonomic destruction than large bodied odontocetes with sturdier bones. Although possibly due to bioerosion, the roots of shark teeth in Bonebed 1 are almost always missing (or incomplete) and superficially appear abraded. The enameloid crowns of these teeth are usually pristine and intact (although occasionally fragmented). Sometimes the majority of the osteodentine ‘core’ (except for a residue of osteodentine remnants) is missing, suggesting that this process only affected the osteodentine and not the enameloid; it is possible that the roots are bioeroded by microborings (see [98]). This has not been observed in any other taxa or localities, and represents a strong bias against the preservation of shark teeth within a single stratum.

#### Articulation

**Figure 19:**
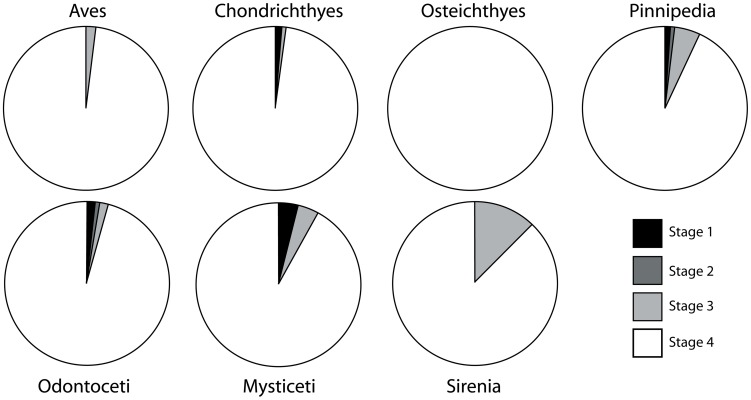
Pie charts showing articulation stage representation in each marine vertebrate taxon.

**Figure 20:**
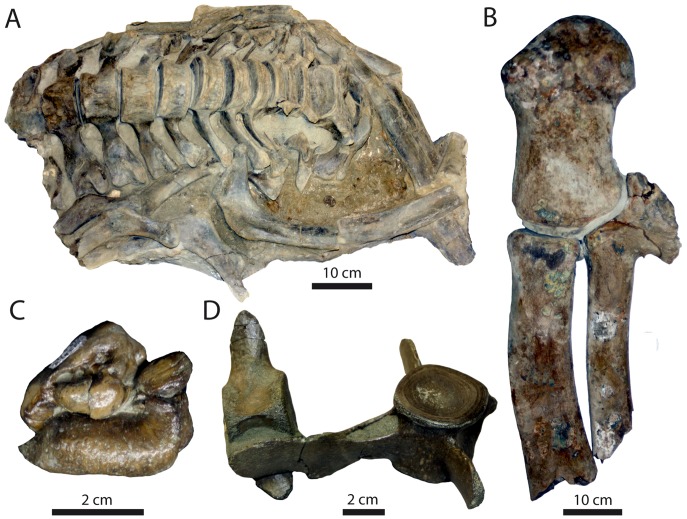
Examples of articulated vertebrate specimens. (A) Articulated thoracic region of medium-sized mysticete (stage 1; UCMP uncatalogued). (B) Articulated mysticete forelimb (stage 1; UCMP uncatalogued, field number FP 107). (C) Articulated tympanic bulla and petrosal of odontocete, Albireonidae indet. (stage 1; UCMP 219511). (D) Associated lumbar vertebrae of odontocete (stage 3; UCMP 219476).

Skeletal articulation was rare in all marine vertebrate taxa examined (Fig. 19). Articulation stage 1–3 was completely absent among bony fish, and rare among marine birds (1.8%), sharks (1.2%), pinnipeds (6.5%), baleen whales (7.3%), and odontocetes (3.9%), while a single associated sirenian skeleton was encountered (stage 3). Examples of articulated and associated specimens are shown in Fig. 20.

#### Fragmentation

Fragmentation follows a slightly different pattern amongst taxa (Fig. 18B). Fragmentation is most frequent in indeterminate mammal bones (83%), sirenians (62%), bony fish (63%), and mysticetes (45%). Skeletal elements of odontocetes, pinnipeds, and sharks all exhibit lower rates of fragmentation (27–33%); most intriguingly, only 22% of bird bones are fragmented. Sirenians and bony fish have extremely dense postcranial bones (pachyosteosclerotic and avascular bone, respectively), and accordingly may fragment due to their higher brittleness; likewise, the fragmented nature of indeterminate mammal bones is the reason why they are unidentifiable. A lower incidence of fragmentation for bird bones may be related to their lower mass and density relative to other vertebrate skeletal elements. Perhaps a bone with lower mass is less likely to suffer an impact with sufficient force to incur fracturing. Similarly, bird bones are only rarely abraded or polished, and in general show little physical taphonomic modification. It is unclear whether this reflects a predominance of “fresh” elements and relatively quick taphonomic degredation, or a genuine resistance to damage due to the lower mass and density of bird bones. Actualistic tumbling experiments using bird bones may address this problem.

#### Polish

Most taxonomic groups show little evidence of polish (Fig. 18C). However, odontocetes, mysticetes, and pinnipeds occasionally show element polishing (25%, 21% and 30%, respectively), and sharks and bony fish exhibit similar frequencies of polish (24 and 31%, respectively). Birds and sea cow bones are rarely polished (6 and 0%, respectively).

#### Phosphatization

**Figure 21:**
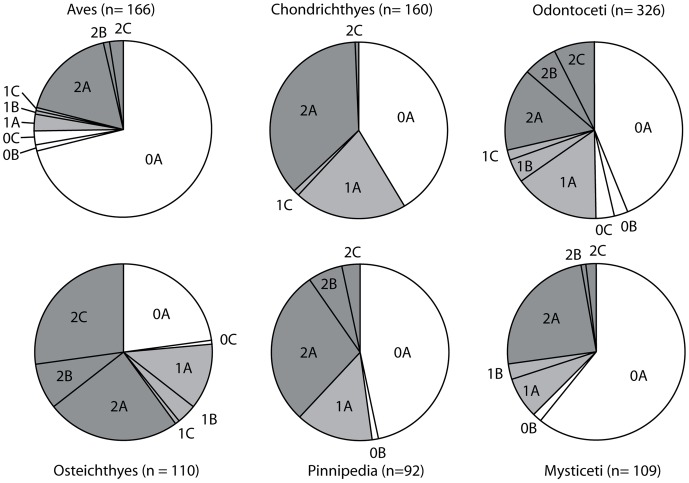
Pie charts showing phosphatization stage representation in each marine vertebrate taxon.

Phosphatization affects the skeletal elements of certain taxa differently (Fig. 21). Bony fish elements are most frequently phosphatized (78%, non stage 0A), while sharks, pinnipeds and odontocetes also share relatively high frequencies of phosphatization (54–59%); bird, mysticete, and sirenian elements have lower frequencies (25–41%). This indicates bias towards the phosphatization of fish, sharks, and small marine mammals. The phosphatized sample of most groups (with the exception of mysticetes) contains a large proportion of blackened, mineralized elements lacking nodules (Stage 2A). Trends regarding the occurrence of nodules are also apparent: birds, sharks, sirenians, indeterminate mammal bones, and mysticetes rarely exhibit adhering phosphatic nodules (Stage XB or XC), while bony fish and odontocetes include a large number of Stage 2C specimens with large overgrowths. Phosphatization only operates within a thin zone below the sediment-water interface, and thus is biased towards small skeletal elements [99], potentially explaining the limited effect on mysticete bones. Pinnipeds exhibit an intermediate amount of nodule-bearing specimens.

Differential phosphatization among these different taxa has numerous implications. While phosphatized elements may be more susceptible to fragmentation due to their increased brittleness, they are probably less sensitive to abrasion than ‘fresh’ elements. Furthermore, phosphatization often increases the density of the element, decreasing the likelihood (or slowing) of transport as bedload, and possibly exaggerating hydrodynamic sorting. Phosphatization may be a mechanism for increasing the preservation potential of an element (prefossilization; [100], [101]) as phosphatized bones appear more resistant to abrasion, with the addition of phosphatic nodules on skeletal elements further inhibiting their taphonomic destruction. Preferential phosphatization of certain taxa (odontocetes and bony fish) may result in a taxonomically skewed assemblage biased towards these taxa by exaggerating hydraulic sorting and increasing the durability of their phosphatized remains.

### Comparisons among Skeletal Element Types

**Figure 22:**
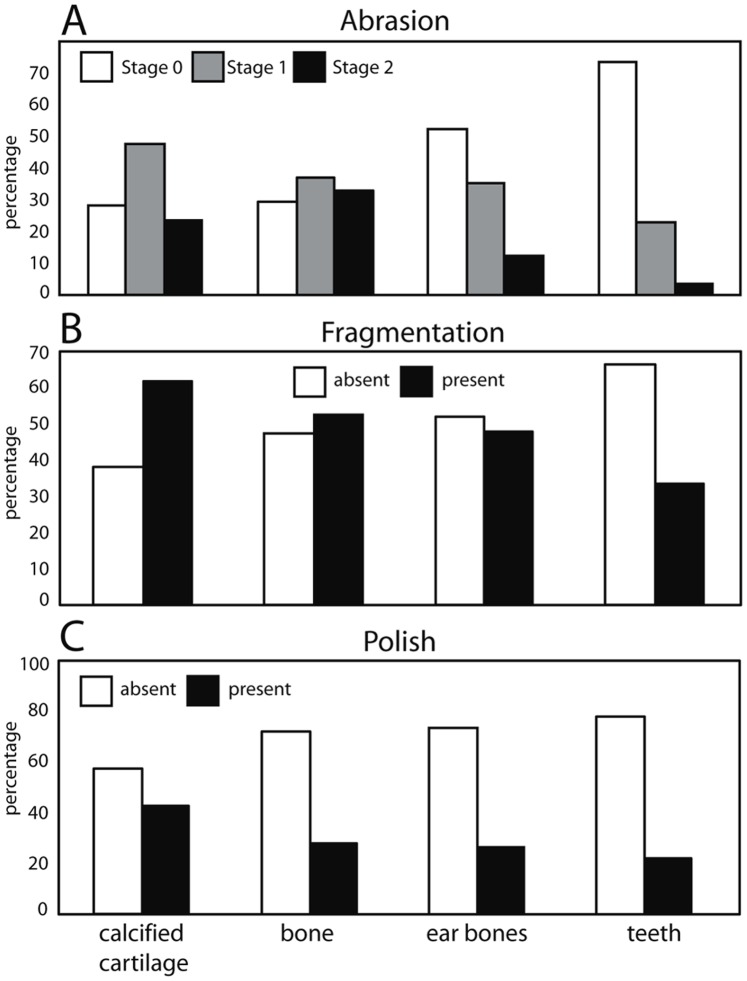
Histograms showing frequency of bone modifications (displayed as percentage of sample) in different groups of skeletal elements. (A) abrasion, (B) fragmentation, and (C) polish.

To address possible bias between different types of biomineralized tissues, skeletal elements from this study were grouped into four broad groups: bones, earbones (restricted to cetaceans), teeth (absent in mysticetes and birds), and calcified cartilage (restricted to chondrichthyes), and compared in terms of abrasion, fragmentation, phosphatization, and polish (Fig. 22). Because these elements vary widely in density and hardness, it is reasonable to hypothesize that they may be subject to preservational bias. Frequency of abrasion in these groups is strongly correlated with the hardness of biomineralized tissue. Calcified cartilage and bones are most frequently abraded (71.5% and 70.7%, respectively; Stage 1–2), and teeth were the least commonly abraded (26.6%), with earbones displaying intermediate abrasion (47.9%). Little difference in fragmentation was evident between bones (52.6% fragmented) and earbones (47.9% fragmented), while cartilage was the most fragmented (61.9%), and teeth were the least affected (33.4%).

**Figure 23:**
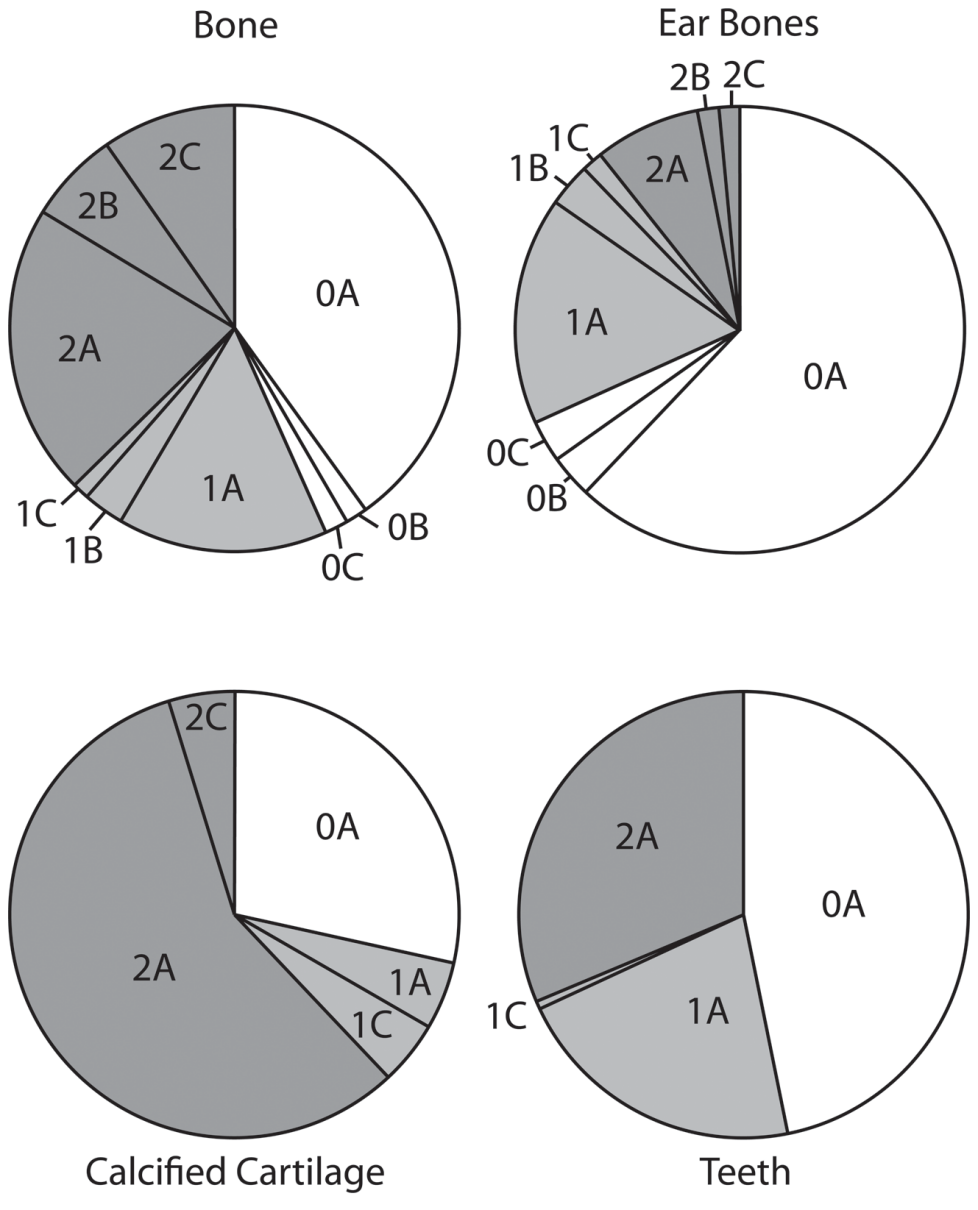
Pie charts showing phosphatization stage representation in each skeletal element group.

Earbones were least affected by phosphatization (57.7% Stage 0A), while calcified cartilage was most frequently phosphatized (28.5% Stage 0A; Fig. 23). The greatest variation in phosphatization stage occurs in bones and earbones, while phosphatized cartilaginous elements are typically Stage 2A (57.1%), and phosphatized teeth are typically Stage 1A (20.9%) or 2A (31.4%). Teeth rarely have adhering phosphatic matrix (Stage XB–C), while bones often do; calcified cartilage and earbones exhibit adhering nodules less often. Intermediate mineralization (Stage 1) is rare in calcified cartilage, suggesting that this tissue type becomes phosphatized rapidly.

**Figure 24:**
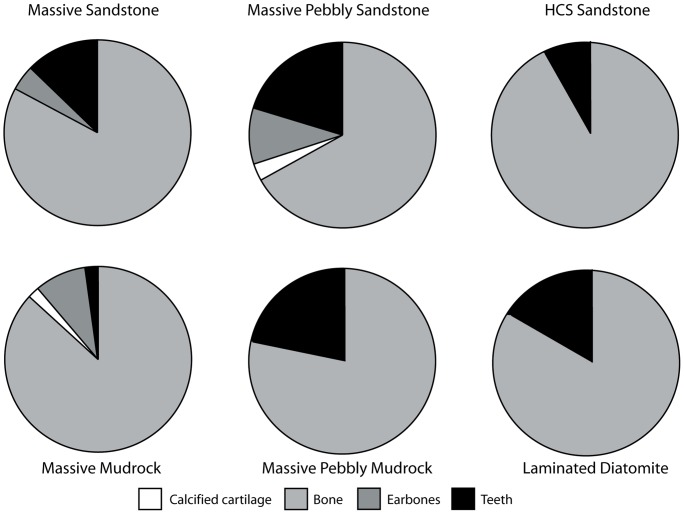
Relative abundance of skeletal element groups within different lithofacies.

When the relative abundance of each tissue type is plotted by lithofacies, several trends are apparent (Fig. 24). Bones are the most abundant type of element in all lithofacies. Teeth are most abundant in bonebeds and in proximal settings. Calcified cartilage occurs only in the massive pebbly sandstone (Spm) and massive mudrock (Mm) lithofacies, while earbones are most common in the massive pebbly sandstone (Spm) and less abundant in the massive sandstone (Sm) and massive mudrock (Mm) lithofacies. This suggests that while bones are the most commonly fossilized elements, teeth, earbones, and calcified cartilage are best represented within bonebed lithofacies and massively bedded offshore sediments. Teeth in particular are only well-represented in proximal settings linked with frequent reworking and disturbance (Shc), slow sedimentation and intermittent reworking (Sm), and periods of widespread reworking of shelf sediments during bonebed formation (massive pebbly sandstone and mudrock; Spm and Mpm, respectively).

## Taphofacies Analysis

To elucidate taphonomic gradients within vertebrate assemblages four vertebrate taphofacies [9], [10] were delineated (Table 5). A taphofacies is a body of sedimentary rock “which is distinguished from other vertically and laterally related bodies of rock on the basis of its particular suite of taphonomic properties” ([8]:227). For the Purisima Formation, taphofacies analysis utilized variation in preservation of vertebrate skeletal elements only. No single taphonomic characteristic (e.g., abrasion) was found to define any single taphofacies (Table 5); thus, combinations of preservational features of vertebrate fossils were used. With the exception of the Bonebed Taphofacies, other taphofacies are designated Taphofacies 1, 2, and 3. The lack of discrete boundaries for any given taphonomic characteristic highlights the gradational nature of marine vertebrate preservation in shelf environments.

**Table 5:** Generalized importance of taphonomic characteristics in Purisima Formation vertebrate taphofacies.

Taphofacies Characteristics	Bonebed Taphofacies	Taphofacies 1	Taphofacies 2	Taphofacies 3
Articulation	–	–	+	+
Abrasion	++	+	+	–
Fragmentation	++	+	+	–
Phosphatization	++	++	+	–
Polish	++	–	–	–
Bioclast abundance	++	+	+	–
Corresponding Lithofacies	Spm, Mpm	Shc	Sm, Mm	Mm, Mld
Inferred Depositional Setting	Much of shelf surface during transgression	Shoreface	Transition Zone	Offshore

Abbreviations: ++ = abundant; + = moderate; − = rare; Mld = Laminated Diatomite lithofacies; Mm = Massive Mudrock lithofacies; Mpm = Massive Pebbly Mudrock lithofacies; Shc = Hummocky Cross-Stratified Sandstone lithofacies; Sm = Massive Sandstone lithofacies; Spm = Massive Pebbly Sandstone lithofacies.

### Taphofacies 1

#### Description

Vertebrate skeletal elements in Taphofacies 1 exhibit a range of taphonomic modifications. Vertebrate material is mostly isolated, and associated specimens are rare. Specimens are occasionally fragmented and often abraded. Vertebrate skeletal elements within this taphofacies display a wider range of phosphatization than in Taphofacies 2 and 3, and slightly more skeletal elements display phosphatization. Roughly one-third of these elements are phosphatized or exhibit adhering phosphatic matrix. Phosphatization is not as common as in the Bonebed taphofacies. Specimens preserved within this taphofacies are rarely polished. Vertebrate skeletal elements occur more often within shell concentrations than in the bioclast-poor “background” sediment layers between bioclastic accumulations. This taphofacies occurs within the hummocky cross-stratified sandstone (Shc), and interfingers with Taphofacies 2.

#### Interpretation

The distribution and taphonomic condition of fossil vertebrate elements preserved indicates a higher energy environment than that of Taphofacies 2 and 3. Isolated vertebrate elements are most often concentrated in shell beds associated with erosional (or hiatal) surfaces. The abundance of vertebrate skeletal elements mantling erosional surfaces and occasional phosphatization (and adhering phosphatic matrix) indicates many of these bones have been exhumed from the underlying substrate. This is corroborated by the abundance of phosphatic nodules and invertebrates with adhering phosphatic matrix in these beds. Bones and teeth devoid of taphonomic modification may represent skeletal input during minor hiatuses or material yet to experience enough transport and burial/exhumation cycles to produce modification.

The higher degree of taphonomic modification in this taphofacies (relative to Taphofacies 2 and 3) is interpreted as being produced by higher energy conditions related to shoreface settings. The abundance of hummocky cross-stratified sandstone and thick shell beds suggests that this taphofacies represents vertebrate skeletal material preserved within shoreface deposits, above storm weather wave base and in some cases above fair weather wave base. Frequent storm reworking is probably responsible for disarticulating and dissociating skeletons [21], and exhuming some vertebrate skeletal material from underlying strata (evidenced by occasional phosphatized elements). This taphofacies reflects shoreface or inner shelf preservation.

### Taphofacies 2

#### Description

Vertebrate skeletal elements in Taphofacies 2 are rarely modified. In general, evidence of fragmentation and abrasion is sparse, affecting only a minority of specimens. Phosphatization and polish of bones is rare. Taphofacies 2 has the highest frequency of articulated and associated skeletons relative to other taphofacies, and isolated bones and teeth are abundant. In some cases, bones and teeth are concentrated within these mollusk shell pavements and thin shell beds; vertebrate skeletal material exhibiting rare evidence of abrasion or fragmentation is confined to these thin mollusk shell concentrations. Vertebrate skeletal elements occur more frequently within these shell concentrations than in ‘background’ sediment. This taphofacies corresponds to the massive mudrock (Mm) and massive sandstone (Sm) lithofacies, and interfingers with Taphofacies 1 and 3.

### Interpretation

Taphofacies 2 represents a combination of attritional accumulation of vertebrate hardparts and occasional concentration of skeletal material along storm-generated erosional surfaces. This taphofacies was deposited near and below storm weather wave base, within the shoreface-offshore transition zone. Vertebrate elements shed from drifting carcasses are likely responsible for the majority of isolated elements preserved ‘floating’ in sediment, as low energy environments below storm weather wave base lack sediment transport processes capable of transporting and dissociating bones. As a result, taphofacies 2 exhibits the largest sample of articulated and associated skeletons that remained relatively complete after arrival at the sediment-water interface. Scavengers and bioturbators may cause some of the disarticulation seen in some specimens. Minor phosphatization shows that at least some elements were exhumed by storm erosion and redeposited by hyperpycnal flow, along with ‘fresh’ skeletal elements from the sediment-water interface. During deposition of the massive sandstone (Sm) lithofacies, occasional storm currents concentrated some vertebrate skeletal material into laterally extensive shell beds and pavements. This taphofacies reflects transition zone or middle shelf preservation.

### Taphofacies 3

#### Description

Vertebrate skeletal elements are extremely rare, distributed randomly throughout the sediment, and rarely mantle surfaces within taphofacies 3. Vertebrate bones and teeth lack taphonomic modification or polish, are unabraded, complete, and unphosphatized. Bones and teeth are typically isolated. Two partially articulated skeletons, including a mysticete skeleton with preserved baleen and a chemosynthetic mollusk assemblage typical of whale falls occur within this taphofacies (F.A. Perry, unpublished data). Aside from the aforementioned molluscan assemblage, mollusks and crustaceans are absent from this taphofacies. This taphofacies corresponds to the laminated diatomite (Mdl) and massive mudrock lithofacies (Mm), and interfingers with Taphofacies 2.

#### Interpretation

This taphofacies represents attritional accumulation of vertebrate skeletal material in distal, mud-rich environments. Vertebrate skeletons and elements occur in mudrock and diatomite deposited in low-energy offshore settings by suspension settling of sediment; no evidence of higher-energy traction transport of sediment or skeletal material is present. Vertebrate skeletal material likely remained at the original site of deposition, with many isolated elements shed from floating carcasses. Shark teeth may have accumulated after being shed during feeding. This taphofacies reflects offshore or outer shelf preservation.

### Bonebed Taphofacies

#### Description

Taphonomically modified vertebrate skeletal concentrations occur in laterally extensive bonebeds that mark vertical lithofacies offsets. Vertebrate skeletal material from the Bonebed taphofacies exhibits the highest degree of taphonomic modification (abrasion, fragmentation, phosphatization). In rare cases, articulated and associated skeletons occur within bonebeds. A cluster of associated mysticete bones occurs in Bonebed 4, and dozens of articulated skeletons are known from subinterval β_2_ of Bonebed 6. Additionally, a single cluster of odontocete vertebrae was observed in subinterval β_3_ of Bonebed 6, and a cluster of sirenian bones representing a disarticulated skeleton within Bonebed 1. Most cases of polished skeletal elements occur within this taphofacies.

#### Interpretation

Bonebeds form from submarine erosion, depositional hiatus, or a combination of the two (Fig. 11). The abundance of phosphatic debris and phosphatized skeletal elements indicates periods of low net sedimentation promoting conditions conducive for phosphogenesis, corroborated by the presence of glauconite. Erosion by fair-weather and storm-generated waves resulted in exhumation and redeposition of ‘prefossilized’ vertebrate skeletal elements in the bonebed assemblage. More poorly phosphatized skeletal elements likely represent specimens that experienced lower duration of phosphogenesis. Fragmentation is more abundant within this taphofacies; perhaps weaknesses form during early diagenesis in buried bones and subsequently result in fragmentation during exhumation and transport. The Bonebed taphofacies does not interfinger with other taphofacies, but cross-cuts all other taphofacies.

## Discussion

### Bonebed Genesis in the Purisima Formation

Although varying in terms of their physical characteristics, all Purisima Formation bonebeds possess a concentration of coarse material (phosphatic clasts, vertebrate skeletal elements, invertebrate skeletal material, terrigenous clasts), extend laterally over several kilometers (where exposed), exhibit a gradational upper contact, and mark vertical offsets in lithofacies (Table 4; Figs. 12–14). In addition, these bonebeds mark the only substantial concentrations of terrigenous pebbles and cobbles within the Santa Cruz section of the Purisima Formation. Bonebeds vary in terms of other characteristics, including their composition (i.e. mollusk-rich, phosphate rich, or terrigenous clast-rich), nature of their lower contact and α-interval, and bioclast and trace fossil content of the α-interval. Previous studies have also explored subtle changes between marine bonebeds [16], [68], [76], [102], [103].

**Figure 25:**
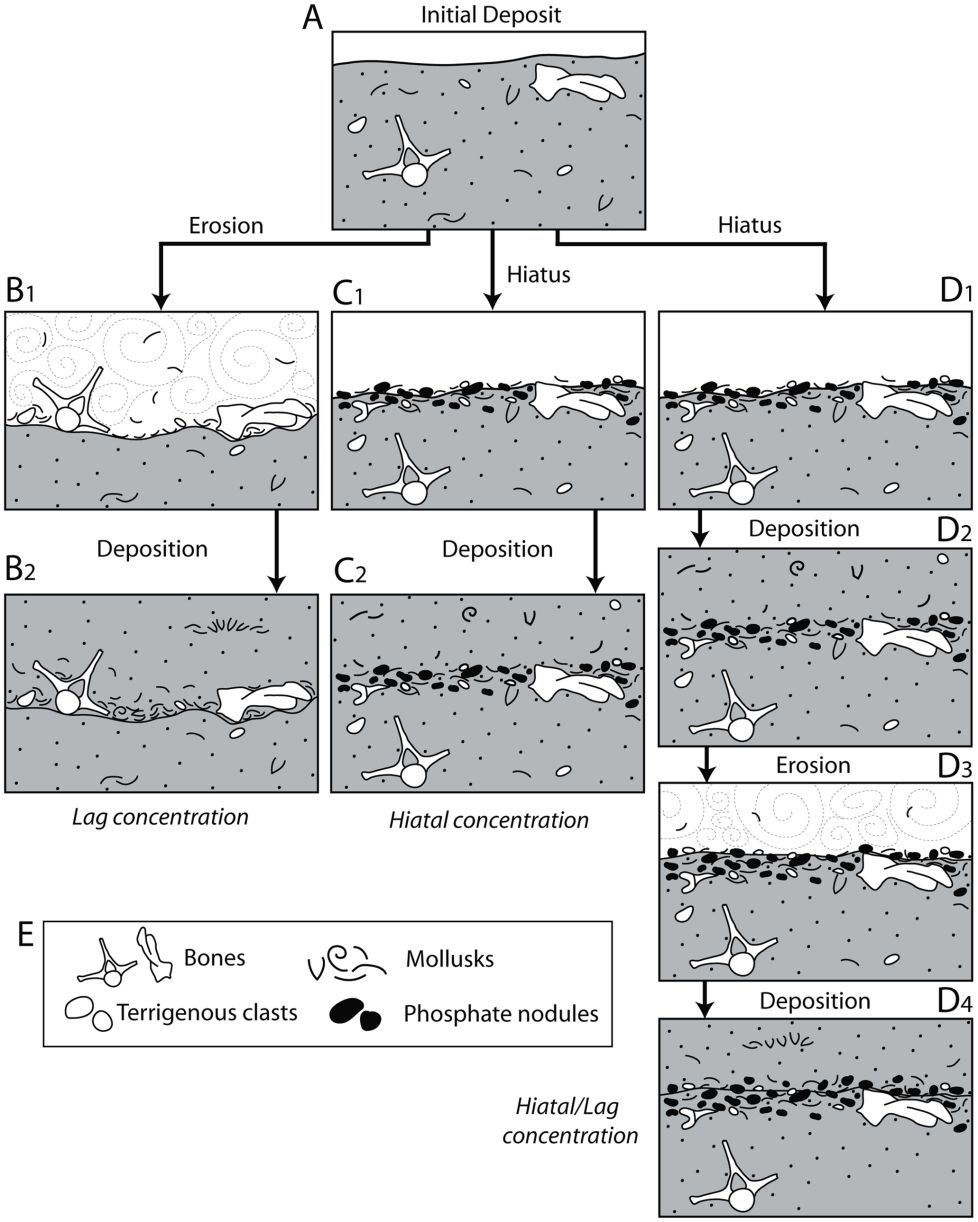
Diagram showing simplified hypotheses of bonebed formation. A) Initial deposit. B1) An erosional event (negative sedimentation) reworks bioclasts and other debris onto an erosional surface, and subsequently buried (B2), resulting in a lag bonebed or concentration. C1) A decrease in sedimentation rate results in a hiatal concentration without a clear erosional surface and a return to ‘normal’ sedimentation buries the assemblage (C2), resulting in a hiatal bonebed or concentration. D1) A hybrid lag/hiatal concentration can be formed, with alternating periods of low sedimentation (D2) and erosion (D3) resulting in a hiatal concentration associated with an erosional lag (D4).

Several bonebeds (Bonebeds 1, 5 and 6) include one or more sharp contacts below or within the bed that clearly indicate a period of erosion and negative net sedimentation (Fig. 13A, 13E, 13F). Where present, these surfaces are directly mantled with the largest and most densely packed bonebed debris. However, while the other bonebeds (Bonebeds 2, 3, 4) lack a preserved internal erosional surface (Figs. 13B–D, 14B–D), all bonebeds contain material exhumed from the underlying substrate. In the case of Bonebed 1, this includes extraformational clasts of the Santa Cruz Mudstone; Bonebeds 2–6 (Fig. 13, 14) contain phosphatic nodules and vertebrate, crustacean, and mollusk bioclasts with adhering phosphatic matrix or nodular overgrowths. The presence of a sharp erosional base (e.g., Bonebeds 1, 5) is evidence that the bonebed is a lag concentration (Fig. 25) [1]. The lack of a sharp erosional surface as in Bonebeds 2–4 could be taken to indicate that it represents a hiatal concentration (Fig. 25). A bonebed with evidence of depositional hiatus and erosion could form composite hiatal/lag concentrations such as Bonebed 6 (Fig. 25).

**Figure 26:**
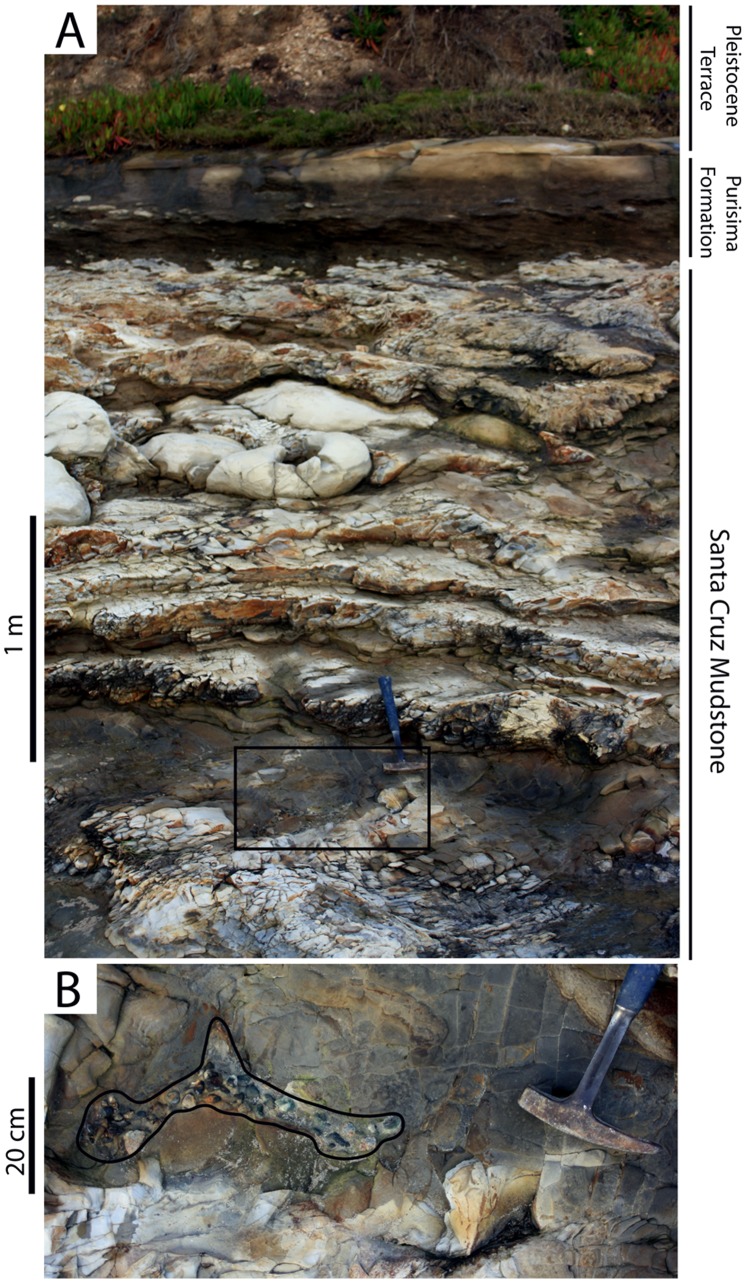
Bonebed debris deposited as fissure fill within burrows below the erosional base of the Purisima Formation (Bonebed 1) at the base of section 1. (A) Photograph of the Purisima Formation-Santa Cruz Mudstone contact; photograph taken on strike with strata at contact, and strata in lower 1/3 of photograph appear oblique due to outcrop shape and parallax. (B) Map-view photograph of pod of Purisima Formation bonebed debris and sandstone infilling *Ophiomorpha* burrows 2.5 meters below base of Purisima Formation.

With the exception of Bonebed 3, all bonebeds are associated with deep trace fossils in-filled with bonebed debris. In most cases the bioclastic fissure-fill in these burrows (or borings) extends up to one meter below the β-interval (e.g., Bonebed 1, 4, 5). However, in Bonebeds 2 and 6, some of this material was observed over 2 meters below the β-interval, and in rare cases, up to 2.5 meters below (Fig. 26); a similar case of *Thalassinoides* traces infilled with bonebed debris 2 meters below a marine bonebed was reported by Martill [104]. While many of these are within *Ophiomorpha* traces, many occur as vertical to oblique, clast-supported subcylindrical pods of bonebed debris that “float” in massive sandstone without any confining trace fossil structure. Bonebed 5 is the only bonebed lacking *Ophiomorpha* traces*–*instead, large *Gastrochaenolites* borings are infilled with bonebed debris (up to 50 cm below the β-interval). The presence of endolithic bivalve boring traces (*Gastrochaenolites*, *Trypanites*) indicates relatively high-energy conditions during some part of bonebed formation [79]. The occurrence of *Gastrochaenolites* at the erosional surfaces of Bonebeds 1, 5, and 6 indicates that at some point during depositional hiatus, the seafloor was partially lithified (a hardground in the case of Bonebeds 5 and 6, and a rockground in the case of Bonebed 1).

Vertebrate fossils in these bonebeds vary in their taphonomic characteristics, but often show high frequencies of fragmentation, abrasion, and phosphatization (Fig. 15). Bonebed 1, for instance, shows a high frequency of fragmented vertebrate skeletal elements, and very few abraded ones. Bonebed 3 is unusual in that it primarily includes unabraded, unfragmented, and unphosphatized vertebrate fossils. Vertebrate remains in Bonebed 4 are typically lightly abraded, lightly phosphatized, and occasionally fragmented. Bonebeds 2, 5, and 6 exhibit abundant bone fragments, abraded bones and bone pebbles, and a majority of the bones are phosphatized. With the exception of Bonebed 1, fragmented bones tend to occur with phosphatized and abraded bones, as well as abundant phosphatic nodules. Most cases of fractured elements are probably due to early diagenetic weakening of bone (during a period of initial burial prior to exhumation and secondary burial), allowing it to fracture longitudinally and transversely upon exhumation.

Four bonebeds (Bonebeds 1, 3, 4 and 6) include articulated and associated skeletons. Bonebed 1 exhibits articulated and associated skeletons somewhat above the bonebed in the γ-interval, while Bonebeds 3, 4, and 6 exhibit skeletons within the β-interval of the bonebed. However, in the case of Bonebed 6, skeletons only occur within subinterval β_2_; within this unit, skeletons appear above a basal lag, grossly similar to the pattern in Bonebed 1. Bonebeds 3 and 4, which preserve skeletons within the β-interval, both consist predominantly of mollusk bioclasts. The occurrence of skeletons above the β-interval is similar to that reported by Pyenson et al. [105] for the Sharktooth Hill Bonebed, who interpreted preservation of skeletons above the bonebed (e.g., within the γ-interval) as resulting from an increase in sedimentation rate after the depositional hiatus which first formed the bonebed. The articulated skeletons in Bonebeds 1 and 6 can be interpreted in this manner as well. However, in Bonebed 6, the erosional surface at the base of subinterval β_3_ has eroded into the upper surface of bones of articulated skeletons preserved within subinterval β_2_ (Fig. 14F), suggesting a return from positive sedimentation (depositing subinterval β_2_) to negative net sedimentation (eroding the B_2_/B_3_ surface). The decrease in bonebed debris subinterval β_3_ indicates an increasing sedimentation rate. The higher mollusk bioclast content of Bonebeds 3 and 4 suggests that their duration of formation was not characterized by periods of phosphogenesis that resulted in preferential dissolution of calcium carbonate bioclasts, such as Bonebeds 2, 5, and 6. Although Bonebed 4 clearly does contain abundant phosphatic debris, the abundance of mollusk bioclasts indicates that conditions sufficient for chemical lag formation were not present. The potentially shorter depositional hiatus during which these concentrations formed during may have been sufficiently brief in order to prevent dissociation of some vertebrate skeletons.

Paleomagnetic studies and diatom floras indicate the occurrence of several hiatuses within the Purisima Formation. These hiatuses coincide with Bonebeds 1, 4, and 6. The hiatus at Bonebed 1 is 0.7–0.5 Ma in duration, based on diatom floras [66], [90]. Bonebed 6 records a depositional hiatus of roughly 1 Ma, from 4.5 to 3.5 Ma [37]. Although probably not of equal duration, Bonebed 4 corresponds to a paleomagnetic reversal [35]; this hiatus was apparently long enough to record a reversal. These data indicate that some of these assemblages are strongly time averaged. Although these data are not available for other bonebeds, other evidence for time averaging abounds. Bonebeds (with the exception of Bonebed 3) include vertebrate skeletal material with a wide variety of taphonomic characteristics, suggesting some degree of mixing. For example, the co-occurrence of abraded phosphatized bone pebbles and pristine bones within Bonebeds 2, 4, and 6 indicate mixing of exhumed prefossilized material with taphonomically ‘younger’ material not yet subjected to exposure, abrasion, burial, exhumation, and fragmentation. As mentioned above, the adhering phosphatic matrix of many bioclasts, phosphatized bones, and phosphatic nodules indicate a significant component of some bonebeds has been exhumed from underlying strata. Additional evidence of long-term exposure of the seafloor during depositional hiatuses (or after an erosional event) includes hardground formation and bivalve borings (*Trypanites* ichnofacies). In the case of Bonebed 1, several features suggest that the Santa Cruz Mudstone was already lithified prior to genesis of Bonebed 1, indicating it formed over a marine rockground. For example, abundant bivalve borings corresponding to the *Trypanites* ichnofacies occur in the Santa Cruz Mudstone along the basal unconformity of the Purisima Formation, and clasts of the Santa Cruz Mudstone within Bonebed 1 frequently exhibit conchoidal fracture in addition to endolithic bivalve borings. Structural features of the Santa Cruz Mudstone at this locality also suggest it was lithified and deformed prior to deposition of the Purisima Formation [66]. Furthermore, several bonebeds (Bonebeds 1, 5, and 6) include glauconite, which forms during periods of low net sedimentation [80].

Most of the bonebeds studied within the Santa Cruz section of the Purisima Formation are associated with vertical facies offsets (Fig. 4). Offsets either occur at the bonebed surface itself (Bonebed 1, Bonebed 5, Bonebed 6) or within one meter above the bonebed (Bonebed 2, Bonebed 3). Bonebed 1 is the unconformable contact with the Purisima Formation and the underlying Santa Cruz Mudstone, and represents an abrupt shallowing change from offshore deposition to transition zone deposition, or a basinward shift in facies. Bonebeds 2–6 all represent the opposite trend: they all represent a shoreward shift in facies, or a relative deepening. For example, massive mudrock underlies and overlies Bonebed 2, and a transition to laminated diatomite occurs one meter above, recording a transition from shallow offshore to deeper offshore sedimentation. Bonebeds 3, 5, and 6 all record a change from hummocky cross stratified sandstone to massive sandstone, representing a transition from shoreface to transition zone sedimentation (Fig. 4). Bonebeds 2–6 represent examples of transgressive erosional surfaces [96]. As opposed to the specific transgressive surface of erosion that occurs within a depositional sequence, marking the boundary between the lowstand systems tract and the transgressive systems tract [96], these bonebeds coincide with parasequence boundaries (marine flooding surfaces). The transgressive surface of erosion forms at the beginning of a transgression; during this period, rivers back up, and sediment is temporarily trapped in estuaries [96]. The decrease in clastic input allows high energy erosional processes (e.g., storm erosion and reworking) to erode the seafloor, resulting in a lag concentration. Although not expressly associated with a transgressive surface of erosion *sensu stricto,* the transgressive lag model applies to Purisima Formation bonebeds at marine flooding surfaces as these still represent relative (albeit minor) transgressions; marine bonebeds coincide with marine flooding surfaces in the Triassic of England [106], [107]. Many marine bonebeds have previously been interpreted as transgressive lags [76], [103]–[106], [108], [109]. Marine bonebeds are known to have other modes of formation [12], [70], [102], [110], [111]; Bonebed 1 is suggestive of a lag forming during a regression, as outlined by Reif [111]; also see Walsh and Martill [70]. Bonebeds in the Purisima Formation were also interpreted to reflect lags or hiatal concentrations related to transgression by other studies [4], [34].

### Bioturbation and Bonebed Architecture

This study and previous research have illuminated the utility of bonebed (or shellbed) cross-sectional architecture for interpreting its mode of formation [1], [6], [105]. Purisima Formation bonebeds vary in terms of thickness, presence or absence of debris-filled trace fossils, bioclast packing, and expression of erosional surfaces. Some bonebeds lack distinct erosional surfaces (Bonebeds 2–4) and instead have gradational upper and lower contacts, while others preserve a distinct basal scour (Bonebeds 1, 5) or multiple scours (Bonebed 6; Fig. 13, 25). Despite lacking an erosive base, Bonebeds 2 and 4 exhibit loosely packed bioclasts/clasts, and *Ophiomorpha* burrows that extend up to 2 meters below the β-interval that are infilled with densely packed bonebed debris. These bonebeds also exhibit clear trace fossils within the bonebed. These data indicate that certain bonebeds have been bioturbated and biologically mixed, directly modifying their internal architecture; such biologically mixed concentrations are difficult to interpret (e.g., [73]). The presence of coarse bonebed debris infilling burrows below bonebeds indicates bioturbating invertebrates were able to transpose clasts and bioclasts up to 5 cm in length, and up to 2–3 meters below bonebeds (e.g., Fig. 26).

**Figure 27:**
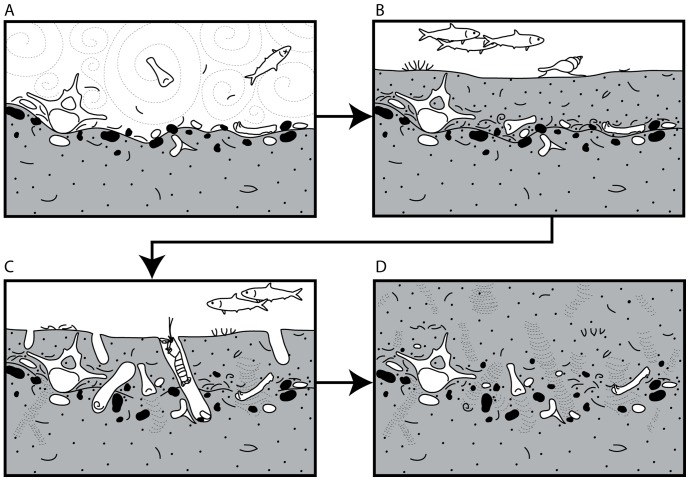
The effects of bioturbation on cross-sectional bonebed geometry. (A) Erosion winnows coarse, dense material and bioclasts onto an erosional surface. (B) A return to positive sedimentation results in burial of the lag concentration. (C) Subsequent bioturbation by burrowing organisms and other infauna erase the erosional surface and transpose bioclasts. (D) The resulting concentration may be misinterpreted as a hiatal bonebed.

This indicates that the architecture of a bioclastic accumulation (when bioturbated) may be misleading when applying the bioclastic concentration model of Kidwell [1]. Bonebeds 2 and 4 both contain a large amount of phosphatic nodules and phosphatized bioclasts, indicating that seafloor erosion was a factor in its formation, although the lack of a clearly preserved scour means its architecture would be interpreted as a hiatal concentration in Kidwell’s [1] scheme (Fig. 25). This has implications for the interpretation of other marine vertebrate bonebeds; for example, Pyenson et al. [105] interpreted the middle Miocene Sharktooth Hill Bonebed as a hiatal concentration rather than a lag concentration due to the lack of evidence of erosion. However, it is possible that an erosional scour was present at some stage, and subsequently erased by bioturbators (Fig. 27); this possibility is borne out by the abundance of fragmented and otherwise taphonomically damaged skeletal elements reported from the Sharktooth Hill Bonebed [105]. These new observations from the Purisima Formation indicate that bonebed architecture – just like primary sedimentary structures – can be biologically modified after deposition, with the potential to drastically affect interpretations of bonebed genesis. Furthermore, this style of information loss means that it may not be possible to determine a mode of formation from bonebed architecture (such as in [1], and [105]) in the case of bioturbated bonebeds.

The α-interval of most bonebeds includes abundant burrows (*Ophiomorpha*, *Gastrochaenolites* ) extending 1–3 meters below the β-interval that contain bonebed clasts and bioclasts (e.g., Fig. 26). Material infilling such traces only occur immediately below bonebeds, and is best interpreted as bonebed debris infilling open burrows at some point during bonebed formation. This demonstrates a surprising potential for bioturbators to rework small (<10 cm) bioclasts down section into older strata; similar down section reworking by bioturbators has been reported by Martill [104]. This has implications for surprisingly old taxa in condensed strata; for example, Koretsky and Sanders [112] reported on surprisingly old true seal (Phocidae) femora from the late Oligocene Chandler Bridge Formation of South Carolina. The formation is less than one meter thick and unconformably overlain by fossiliferous Pleistocene strata; in theory, it is possible that Plio-Pleistocene fossils could be reworked by contemporary bioturbators into older strata. This applies equally to other cases of unexpected fossils appearing in stratigraphically condensed sections.

### Relationship between Phosphatization and other Taphonomic Characteristics

**Figure 28:**
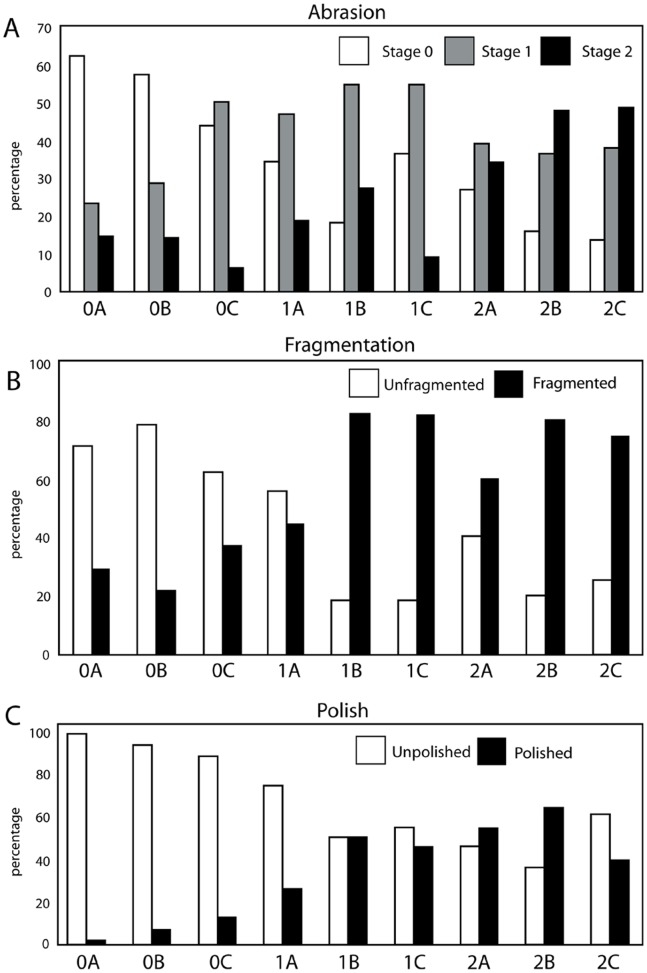
Histograms showing relationship between phosphatization stage and other bone modifications. Bone modifications include (A) abrasion, (B) fragmentation, and (C) polish; values displayed as percentage of elements showing each phosphatization stage and bone modification.

The majority of polished elements (73%) also exhibit Stage 2 phosphatization, with the frequency of polish and phosphatization stage appearing to be positively correlated (Fig. 28). This suggests that polish primarily occurs after prefossilization, as suggested by Rogers and Kidwell [101], and that phosphatization is a common mode of prefossilization in the shallow marine fossil record. Abrasion and phosphatization show a similar trend. Specimens with adhering nodules and stage 1–2 permineralization are more frequently abraded. Stage 2 specimens are the most abraded. A similar relationship also exists between fragmentation and phosphatization (Fig. 28B); specimens with adhering nodules and stage 1 and 2 phosphatization are more frequently fragmented than stage 0 specimens and those without nodules. Specimens with stage 1A and 2A phosphatization are roughly similar, while 1B, 1C, 2B, and 2C specimens are more fragmented (but roughly similar to each other), suggesting that style rather than the degree of phosphatization has more of an effect on fragmentation.

The higher degree of abrasion, fragmentation, and polish among phosphatized elements (Fig. 28) is potentially confounding, given the hypothesis that prefossilized elements would be more durable [101]. It is possible that increased abrasion and fragmentation are related to processes of erosion implicit in the concentration of phosphatized material, rather than being evidence of decreased preservation potential. Experimental abrasion experiments indicate that prefossilized vertebrate teeth are more resistant, or can withstand long periods of tumbling with little apparent abrasion [113], [114].

### Biogenic Bone Modifications

**Figure 29:**
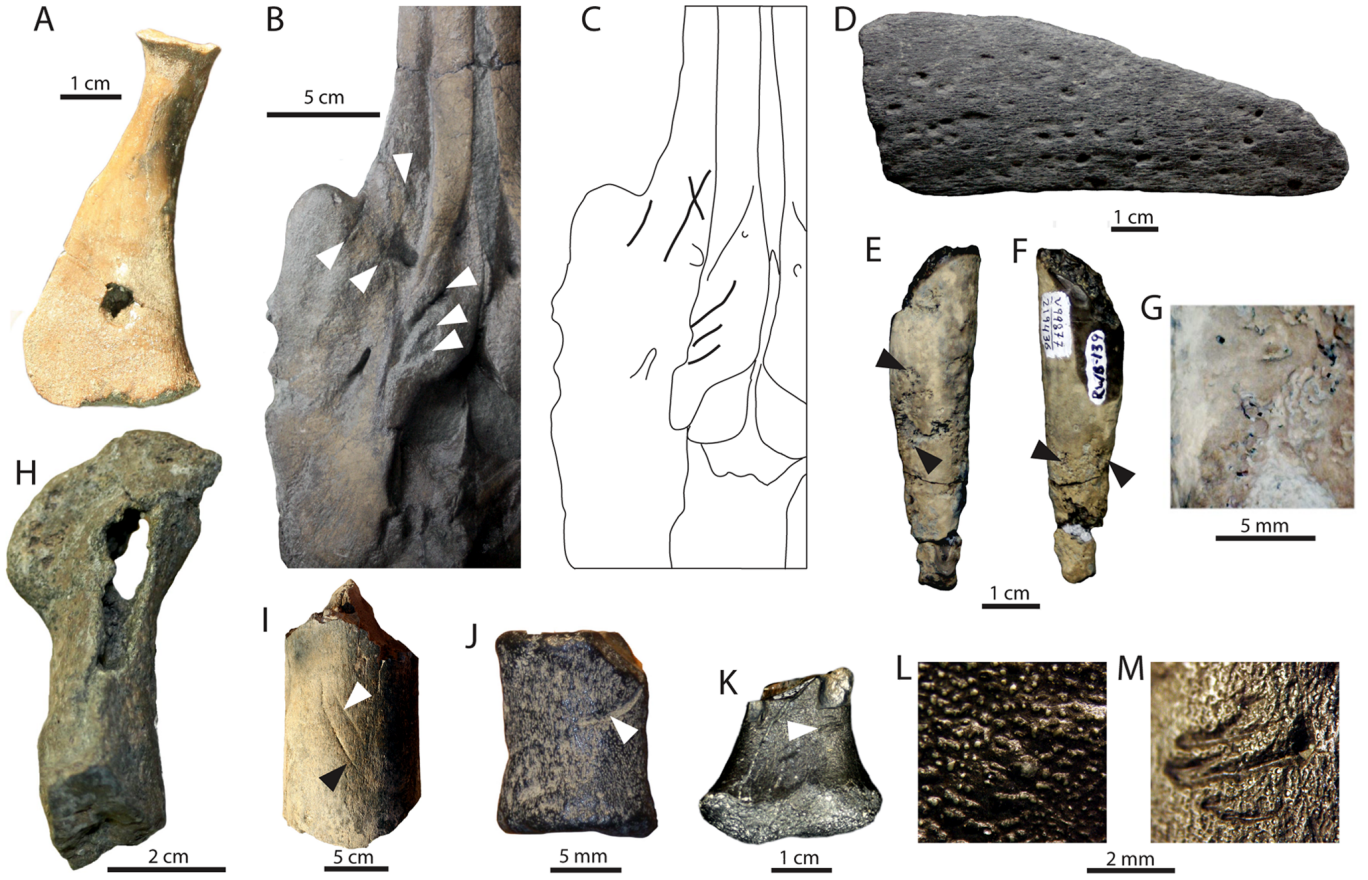
Examples of biogenic bone modifications encountered during this study. (A) fur seal radius (Pinnipedia: Otariidae, UCMP 219009) with mammalian bite marks (see Boessenecker and Perry, 2011). (B) Porpoise cranium (Odontoceti: Phocoenidae, undescribed genus; UCMP 219504) with two sets of linear bite marks (C). (D) Bone fragment (Mysticeti indeterminate; UCMP uncatalogued) with numerous circular pits. (E–F) Walrus tooth (Pinnipedia: Odobenidae: Dusignathinae; UCMP 219436) with irregular bioerosion tunnels; (G) magnified view of bioeroded surface. (H) Dolphin humerus (Odontoceti indeterminate; UCMP 219361) with bioeroded gallery. (I) Baleen whale mandible fragment (Mysticeti indeterminate; UCMP 219089) with parallel linear tooth marks. (J) Dolphin phalanx (Odontoceti indeterminate; UCMP 219627) with linear tooth mark. (K) Fur seal distal femur (Pinnipedia: Otariidae: *Thalassoleon* sp., cf. *T. macnallyae*; UCMP 219658) with gastric acid pitting (L) and linear scrape marks (M).

Taphonomic modifications of biogenic origin (Fig. 29) are generally rare in the available sample (1.6% of specimens), and include linear scrapes (n = 7), circular depressions (n = 2), excavated galleries (n = 3), circular bioerosion pits (n = 2), bioerosion tunnels (n = 1), and surface pitting (n = 2). Other vertebrate fossil assemblages sampled in a similar manner have also yielded low numbers of biogenic modifications (<1%; [76]); although such modifications are commonly reported in the literature ([52], table 1; [115], table 1), the Purisima Formation assemblage suggests they are relatively rare in the fossil record. Examples of vertebrate skeletal elements encrusted by invertebrates are entirely lacking from the Purisima Formation; this seems to be the rule rather than the exception, as few marine vertebrate assemblages have yielded more than a few encrusted specimens (see review in [116]), with the exception of the Pliocene of Italy [117]).

**Figure 30:**
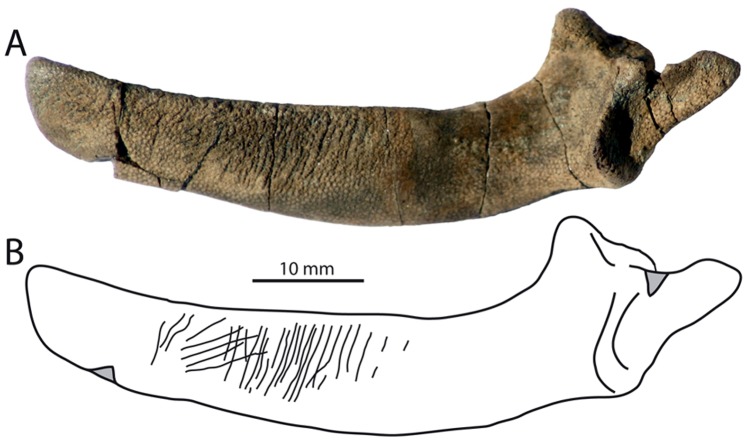
Calcified mandibular cartilage (A) of skate (*Raja* sp., cf. *R. binoculata*; UCMP 219035) with two sets of linear bite marks (B).

Linear scrapes occur on a number of pinniped, odontocete, and mysticete bones (Fig. 29B, C, I–K, M). Such scrapes are frequently interpreted as shark tooth bite marks [118], [119]. Another specimen (UCMP 219035), from a separate Purisima Formation locality reported by Boessenecker ([43]; and thus, not part of the Santa Cruz sample) represents a calcified mandibular cartilage of a skate (*Raja* sp. cf. *R. binoculata*) with a series of subparallel (some cross cutting) linear gouges on its ventral surface (Fig. 30). This occurrence probably also represents shark feeding traces, as well as the first known record of bite marks preserved in fossilized cartilage (i.e. rather than bone); similarly, a *Squalicorax* tooth has been reported embedded in the mandibular cartilage of the shark *Cretoxyrhina* [120]. All these examples appear to represent damage from a single tooth cusp, possibly assignable to the ichnotaxon *Linichnus serratus* [121]; no examples of parallel wavy grooves such as the ichnotaxon *Knetichnus parallelum* [121] have been observed, interpreted as numerous serrations of a single tooth dragging across the bone surface [118]. A separate type of feeding trace is represented by circular punctures reported by Boessenecker and Perry [52] in two fur seal bones (UCMP 219008, 219009) from the Purisima Formation (Fig. 29A). These traces were interpreted as (possibly marine) mammal bite marks, made by canines or widely spaced conical odontocete teeth; such circular bite marks are also known from juvenile archaeocete whale crania from the Eocene of Egypt [122], [123] and from the skull of a modern human diver killed by a leopard seal in Antarctica [124]. These traces have recently been named *Nihilichnus nihilicus* [121], [125].

One partial juvenile fur seal femur (UCMP 219658) from Bonebed 2 not only displays several dozen minute linear scrape marks, but also a distinctive pattern of surface pitting (Fig. 29K–M). Microscopic observation of the bone surface indicates that the outermost layer of cortical bone is degraded and pores in the underlying cancellous bone enlarged, forming a sponge-like surface pattern (Fig. 29L); these enlarged pores are approximately 0.1–0.5 mm in diameter. This pattern of surface pitting has previously been documented in shark-bitten dinosaur and plesiosaur bones [126], [127], plesiosaur remains preserved as gut contents within a mosasaur [128] and dinosaur bones identified as gut contents of a tyrannosaurid [129]. This surface pitting is indistinguishable from that identified by Varricchio [129] and interpreted as partial acid digestion of bones. In the case of the Purisima Formation specimen, the co-occurrence of possible gastric acid surface pitting and linear scrapes suggests that this element was consumed by a shark, leaving a physical record of digestive acid-etching.

Several types of traces are less easily interpreted. These include bioeroded galleries and tunnels, and circular bioerosion pits (Fig. 29 D–H). The galleries are large, and appear to represent preferential damage to cancellous (rather than cortical) bone (Fig. 29H). Such traces are known on human bones trawled from the seafloor and interpreted as the result of gastropods rasping away bone during algae feeding [130], although it is possible that such a trace can be formed on gut residues by gastric acids [128]. Small, 5–10 mm wide pits in certain specimens (Fig. 29D) are similar to and best interpreted as the “pockmark stage” of bioerosion traces by the bone-eating worm *Osedax* [131]. Similar pockmarks are evident on an abraded cetacean rib fragment (Fig. 29D), but given the surficial abrasion it is not possible to identify the taphonomic agent. Lastly, a single dusignathine walrus tooth (upper third incisor; UCMP 219436) exhibits an extensive series of meandering tunnels which in places have destroyed nearly 50% of the tissue (Fig. 29E–G). Tunnels in bones and teeth identified as microbial or fungal in origin [98] are much smaller than in this specimen; it is unclear if these tunnels represent *Osedax* as CT analysis has not been conducted, although Kiel et al. [132] recently reported *Osedax* borings in Oligocene mysticete teeth.

### Preservation of Calcified Cartilage

The Purisima Formation vertebrate fossil assemblage includes a number of calcified cartilaginous elements from the skate *Raja* sp., cf. *R. binoculata* (e.g., Fig. 30). Boessenecker [43] described and figured a number of these specimens from the San Gregorio section of the Purisima Formation. These include calcified palatoquadrate and mandibular cartilages preserved as isolated elements in shell- and bonebeds. Calcified cartilage is rarely preserved in the fossil record, and when preserved, is typically found in articulated shark skeletons in quiescent offshore sediments and konservat lagerstätten [133]. However, in rare cases, fragments of calcified prismatic cartilage occur as isolated ‘competent’ clasts in time-averaged assemblages [134]. Abundant well-preserved complete cartilaginous chondrichthyan skeletal elements, in the case of the Purisima Formation, are unusual and unprecedented. The fact that only mandibular and palatoquadrate cartilages of a single taxon are present suggests that this chondrichthyan had more strongly biomineralized jaw cartilages than other sharks and rays.

### Phosphatization of Articulated Remains

**Figure 31:**
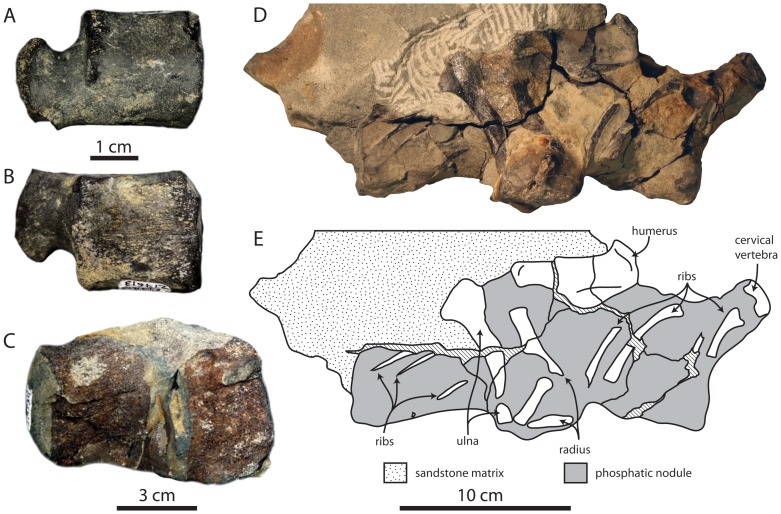
Examples of articulated remains in phosphatic nodules. (A) Odontocete caudal vertebrae in near articulation from Bonebed 6 (UCMP 219584); (B) reverse. (C) Articulated odontocete caudal vertebrae in phosphate nodule from Bonebed 6 (UCMP 219613). (D) Fur seal (*Thalassoleon* sp., cf. *T. macnallyae*) articulated partial skeleton in phosphate nodule from Bonebed 4 (UCMP 219671); diagram of skeleton in E.

Although most phosphatized elements are isolated, three cases of articulated remains occurring in phosphatic nodules were encountered in the Purisima Formation assemblage (Fig. 31). Two specimens are from Bonebed 6, both including a pair of odontocete caudal vertebrae occurring in small phosphatic nodules (Stage 2C) – one in articulation, the other pair in near articulation (UCMP 219613 and 219584, respectively). The third specimen (UCMP 219671) is a large (∼30 cm long) phosphatic nodule containing an articulated thoracic region of a juvenile fur seal *Thalassoleon macnallyae* from Bonebed 4 (Fig. 31D–E). This specimen includes the posterior cervical and thoracic vertebrae, articulated ribs, and articulated humerus, radius, and ulna of both forelimbs. The transverse processes of the cervical vertebrae, lateral surfaces of the ribs, and lateral surfaces of the forelimb elements protrude from the nodule. All three of these specimens are best interpreted as having large phosphate nodules forming after burial of an articulated skeleton. Each skeleton was probably more complete prior to reworking, but the phosphatic nodules may have only formed around isolated parts of the skeleton, or broken apart during reworking. These large skeleton-bearing nodules – and other nodules containing complete odontocete crania – indicate that only a single phase of phosphatization below the sediment-water interface is necessary to form even large phosphatic nodules, in contrast to the postulated mode of nodule formation by multiple phases of reworking and phosphatization termed “Baturin cycles” [135].

### Relative Taxonomic Abundance

**Figure 32:**
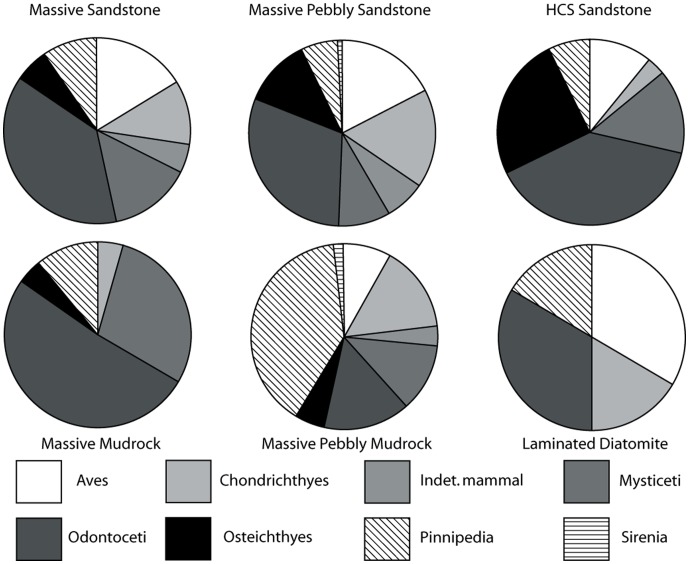
Relative abundance of marine vertebrate taxa within different lithofacies of the Purisima Formation.

Vertebrate taxa are not preserved in uniform proportions across different lithofacies in the Purisima Formation. Several trends are apparent (Fig. 32): 1) bird and bony fish remains appear to be more abundant in proximal settings and bonebeds, and less frequently present in distal settings; 2) shark remains are common in “transition zone” sediments and bonebeds; 3) cetaceans are well represented in most lithofacies; 4) pinnipeds are generally rare but abundantly represented within the massive pebbly mudrock lithofacies (Mpm); 5) indeterminate mammals occur only in bonebeds and the Massive Sandstone lithofacies. The restriction of indeterminate mammal elements can be interpreted as a taphonomic artifact, as most indeterminate mammal elements are highly taphonomically modified and relegated to some of the highest energy environments of deposition. It may be tempting to interpret these data as genuine paleoecologic signal; however, these relative abundance data are influenced by preservational bias to an unknown degree [136] and paleoecologic interpretation is not attempted.

### Comparisons of Invertebrate and Vertebrate Preservation in the Purisima Formation

This study offers an excellent opportunity to compare taphonomic observations of vertebrates with those of the invertebrate assemblages analyzed by Norris [34]. Most obviously, fossil mollusks are locally ubiquitous and numerically more abundant within the Purisima Formation, whereas vertebrate remains are relatively scarce. Bioclastic accumulations, with the exception of some bonebeds (e.g., Bonebed 1, 2, 5) tend to be dominated by mollusks and other calcareous invertebrates (barnacles). Mollusks are most abundant within the upper 1/3 of parasequences, where they occur in shell beds of variable thickness; shell bed frequency (per vertical meter) and thickness increases upsection (i.e. towards shallower depositional settings; [34]). In contrast, vertebrate remains are generally scarce throughout a parasequence (with a slight increase in abundance in the upper part, where vertebrate remains are concentrated along with mollusks), and are abundant in bonebeds. In contrast to patterns of shell bed variation, all bonebeds are relatively thin and characterized by more extreme bioturbation. This evidence suggests that processes of bonebed and shellbed formation, while sharing in common processes involving sedimentary hiatus and erosion – operate at different time scales.

Mollusks are preferentially absent from some bonebeds (Bonebed 1, 2, 5 and some parts of Bonebed 6), indicating the formation of chemical lags within the Purisima Formation. This suggests that in some cases, processes of bonebed and shellbed formation can be mutually exclusive. Similar to shellbeds, bonebeds change in character across the shelf – although bonebeds appear to be more laterally extensive, probably relating to more widespread hiatus and erosion during changes in sea level [106], [107]. Unlike shellbeds, changes in bonebeds are not related to influences of in-situ hardpart production (e.g., encrusting organisms, community beds, and ecologically condensed beds; [34]), and variation in bonebed character is tied instead to varying physical conditions and sediment transport processes. Decapod crustacean remains do tend to mirror that of vertebrates – they are frequently phosphatized or associated with nodules, and abundantly preserved within bonebeds (and generally rare outside bonebeds).

### Onshore-Offshore Trends in Preservation

Several trends in taphonomic conditions are evident among non-bonebed lithofacies. Vertebrate skeletal elements preserved in the hummocky cross stratified (Shc) lithofacies exhibit a higher degree of taphonomic modification than those preserved within the massive mudrock (Mm) and laminated diatomite (Mdl) lithofacies, with fossils from the massive sandstone (Sm) lithofacies displaying an intermediate taphonomic condition. Abrasion and fragmentation of vertebrate skeletal elements follow this pattern, probably relating to decreasing energy and traction transport in successively deeper environments. Aside from bonebed assemblages, abrasion is most frequent within the hummocky cross-stratified sandstone (Shc) lithofacies. Above fair weather wave base, fair weather currents continually rework and transport sandy sediment with vertebrate bioclasts. Below fair weather wave base, less frequent storm-generated combined flow disturbs, transports, and exhumes vertebrate skeletal elements; as storm weather wave base is approached (preserved in the massive sandstone lithofacies), only the most powerful storms are able to disturb seafloor sediments. In deeper water, the frequency and magnitude of storm disturbance decreases, resulting in a decrease in abrasion. Abrasion results from prolonged residence of a skeletal element along an unstable, mobile sandy substrate at the sediment-water interface, and is presumably most extreme during periods of erosion and sediment starvation; persistent oscillatory currents of the middle to upper shoreface have the most potent ability to abrade vertebrate skeletal elements as they can indefinitely abrade a clast or bioclast *in situ* with minimal net transport. Abundant fragmentation occurring by fracturing of brittle prefossilized skeletal elements during exhumation and reworking characterizes vertebrate skeletal elements in the more proximal shoreface massive sandstone (Sm) and hummocky cross-stratified (Shc) lithofacies, but is less common in the offshore massive mudrock (Mm) and laminated diatomite Mdl) lithofacies (Fig. 15, 32).

**Figure 33:**
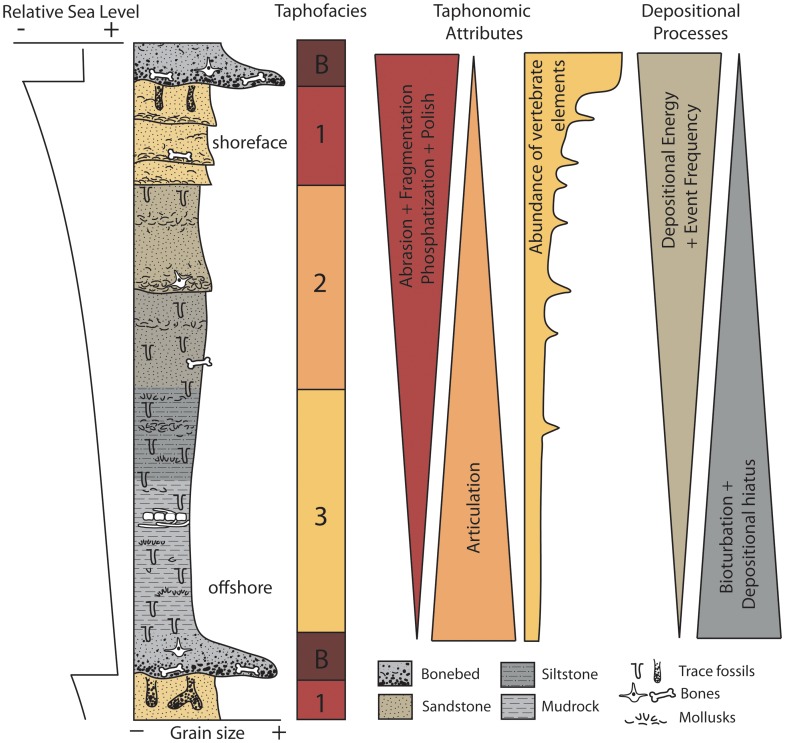
Generalized taphonomic trends within a hypothetical parasequence of the Purisima Formation. This is based chiefly on Section 3, and shows changes in relative sea level, depositional setting, assigned taphofacies, and vertical changes in magnitude of taphonomic attributes and importance of depositional processes.

**Figure 34:**
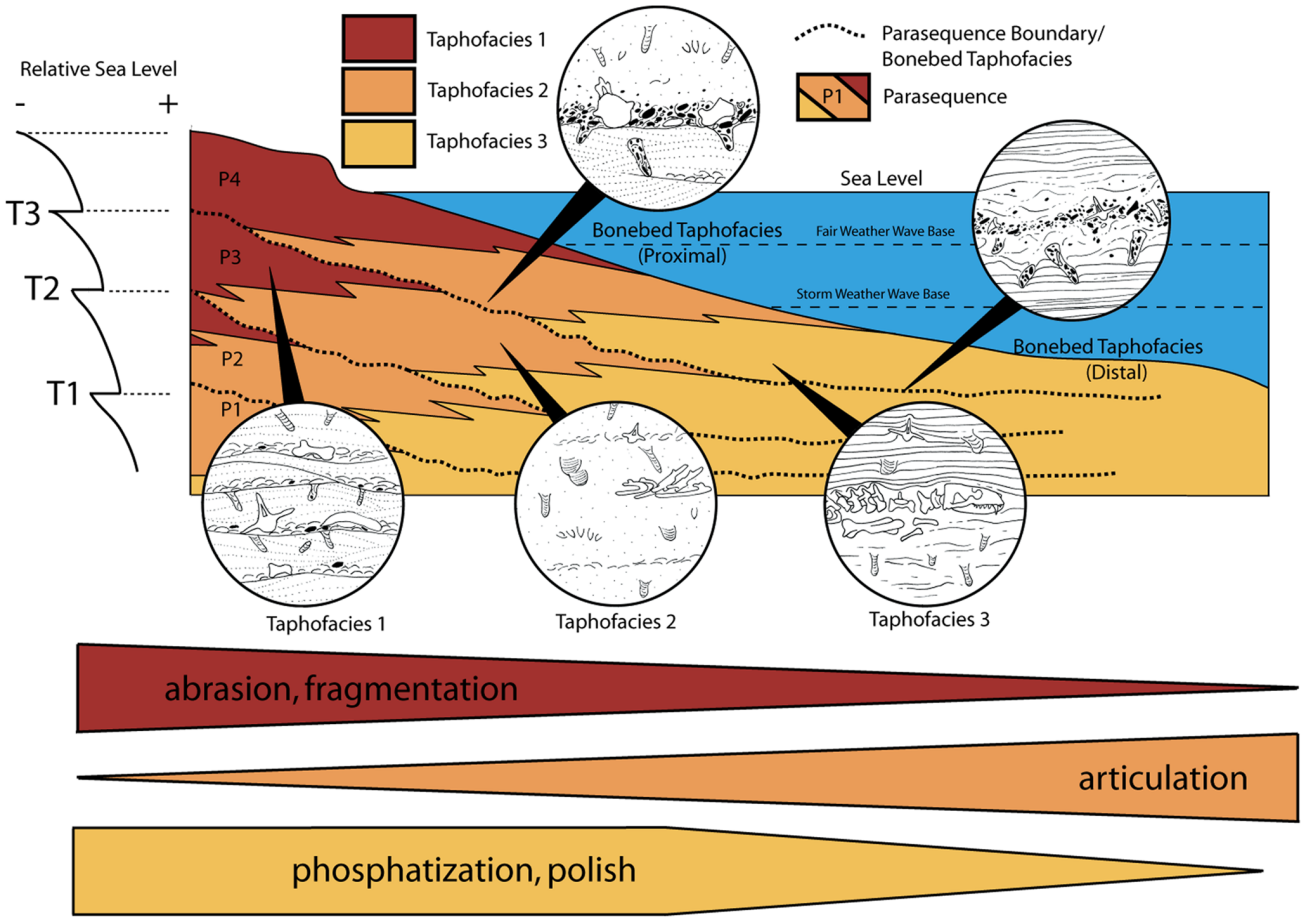
Cross-sectional taphofacies model of the Purisima Formation. Cross section of shelf is divided into taphofacies, and dashed lines representing bonebeds/parasequence boundaries; sea level shown on the left. Insets show diagrammatic representations of each taphofacies, including proximal and distal examples of the bonebed taphofacies. Relative impact of taphonomic attributes are shown at bottom.

Articulation and association of vertebrate skeletons is most common in the massive sandstone (Sm) and massive mudrock (Mm) lithofacies (Fig. 16), a trend opposite to that of abrasion and fragmentation (Fig. 15A–B). Articulated and associated remains are by no means common, and are generally rare in all lithofacies (Fig. 16). However, an increase in the relative abundance of preserved skeletons is observed in further distal, finer-grained lithofacies and likely records carcasses sinking to their final burial. Preservation of articulated skeletons above fair weather wave base in the upper shoreface is unlikely due to the frequency of fair-weather and storm currents that erode and transport sediment at the seafloor [14], [29], leading to frequent reworking, disarticulation, and dissociation of skeletons [21]. Abundance of skeletons (articulated or disarticulated) increases in the lower shoreface and transition zone massive sandstone (Sm) and massive mudrock (Mm) lithofacies due to decreasing frequency of storm disturbance and erosion. Although the data are too few to support correlation of degree of skeletal articulation with paleobathymetry [29], the higher abundance of associated remains (whether articulated or disarticulated) in distal settings fits this trend (Fig. 33, 34), similar to that reported by Soares [14] and Danise et al. [117].

Phosphatized vertebrate material does not simply increase or decrease in an offshore direction, but rather is most common in massive pebbly sandstone (Spm), massive pebbly mudrock (Mpm), and hummocky cross-stratified sandstone (Shc) lithofacies (Fig. 16) indicative of erosion and truncation of preexisting strata (Fig. 33, 34). Because phosphogenesis typically occurs below the sediment-water interface [74], [75], [99], inclusion of phosphatized skeletal material indicates some exhumation from underlying strata, suggesting a control by the erosional capability of a depositional environment.

While some patterns of abundance can be explained by collecting bias towards bonebeds (see above), the decreasing abundance of vertebrate skeletal elements from proximal to distal shelf environments appears to be real (Fig. 33). Extensive winnowing of sediments in the upper shoreface results in exhumation and concentration of vertebrate skeletal elements and mollusk shells into shell beds. As winnowing becomes a less frequent process in progressively deeper water settings, vertebrate skeletal material becomes less abundant. Shelly concentrations are thinner and rare within offshore sediments of the Purisima Formation [34], attesting to a more complete stratigraphic record and more continuous sedimentation. Vertebrate skeletal material is numerically rare in offshore deposits (e.g. massive mudrock (Mm), laminated diatomite (Mld) lithofacies) because their environments of deposition lack depositional processes capable of winnowing coarse material and eroding the seafloor, whereas vertebrate skeletal material is more abundant within thicker shell beds in proximal and mid-shelf settings (e.g. hummocky cross-stratified sandstone (Shc), massive sandstone (Sm) lithofacies) owing to more intense winnowing of sediments above storm weather wave base.

Purisima Formation bonebeds differ in the relative degree of various taphonomic conditions, abundance of invertebrates, abundance of phosphatic debris, and cross-sectional architecture; these differences are likely due to changes in the duration of hiatus, depth of erosion, physical characteristics and fossil content of the eroded sediment, pore water chemistry below the sediment-water interface, and water depth at the time of bonebed formation. While it is difficult to distinguish between these different possibilities, some bonebeds can be discussed in the context of onshore-offshore gradients based on their bracketing lithofacies.

Lithofacies that occur above and below Purisima Formation bonebeds constrain the environment of bonebed formation. For example, Bonebeds 5 and 6 are bracketed by hummocky cross-stratified sandstone (Shc) below and massive sandstone (Sm) above, which limits their deposition to the lower shoreface to transition zone. Likewise, Bonebed 2 is bracketed above and below by massive diatomite, constraining its deposition to the offshore. Bonebed 2 differs from Bonebeds 5 and 6 in its lack of invertebrate remains, phosphatic steinkerns of invertebrates, and an associated erosional surface. Bonebed 4 also lacks an erosional surface but contains abundant calcareous invertebrate skeletal elements and steinkerns, and is bracketed above and below by massive siltstone, suggesting an intermediate zone of formation within the offshore environment but in closer proximity to storm weather wave base. Using Bonebeds 5 and 6 as representative of proximal, Bonebed 2 of distal, and Bonebed 4 of intermediate bonebed deposition, it is possible to make several generalizations about depth-related trends in bonebed taphonomy. The abundance of calcareous invertebrate skeletal elements and phosphatic steinkerns decreases in distal bonebeds, probably reflecting an offshore decrease in the abundance of invertebrate skeletal material in offshore sediments in the Purisima as noted by Norris [34]. Invertebrate remains (calcareous or phosphatized) are only abundant in bonebeds that truncate shoreface deposits, where invertebrate skeletal material is more abundant and concentrated [34].

Surfaces are preserved only in proximal bonebeds (e.g., Bonebeds 5–6), and the application of the skeletal concentration model of Kidwell [1] suggests that distal bonebeds with gradational lower contacts (Bonebeds 2 and 4) represent hiatal concentrations. Although the surrounding sediment is intensely bioturbated above, below, and within these distal bonebeds, it is likely that any erosional surface that may have formed was destroyed by bioturbation (Fig. 27). Additionally, the abundance of phosphatic nodules and other phosphatized material (e.g., steinkerns, phosphatized bones) indicates that erosion has exhumed and concentrated phosphatic debris, although it is possible that this material may have been transported downshelf from a more proximal portion of the bonebed where submarine erosion was persistent. Bonebed 4 represents formation in the transition zone, where storm effects are rare and sediment starvation prolonged. These rare storms, however, are evidently sufficient to exhume large phosphatic nodules that often form around clusters and parts of shell beds, and in rare cases articulated vertebrate remains and entire skulls. In proximal areas of bonebed formation (e.g., Bonebeds 5–6), more frequent disturbance of the substrate results in shorter duration hiatuses unable to produce large nodules. In offshore bonebeds (e.g., Bonebed 2), a similar decrease in phosphate nodule size is observed. Here, slow deposition affords conditions conducive to phosphogenesis, with storms generally unable to disturb the offshore seafloor (except during times of changing relative sea level). Thus, phosphogenesis appears to characterize the ‘middle shelf’ and somewhat decreases in importance proximally and distally (Fig. 33, 34).

Because discontinuities within strike-slip basins are widespread and often cut across facies across an entire basin [137], it is possible that a single bonebed may span nearly the entire shelf (Fig. 12, 33). A single bonebed may change in character along an onshore-offshore transect, depending upon the depositional setting, nature of preexisting sediment, and associated fossil assemblage. This may cause problems for correlation as bonebeds are often used as datum planes (several bonebeds in the Purisima Formation have been used as marker beds for paleomagnetic studies; [37]). For example, the cross-sectional geometry of Bonebed 4 changes along strike.

These trends within non-bonebed lithofacies and bonebeds all appear to be strongly correlated to changes in physical processes that characterize each environment, including frequency and depth of fair-weather and storm-weather disturbance, sedimentation rate, and hiatal duration. Altogether, the absolute abundance of vertebrate skeletal elements is directly related to the duration of depositional hiatuses in the rock record: bonebeds represent the highest concentration of vertebrate bioclasts and the longest period of nondeposition, whereas offshore deposits (e.g., massive mudrock (Mm) and laminated diatomite (Mld) lithofacies) represent the lowest concentration of vertebrate bioclasts and the most complete sedimentary record. Shell beds reflect time averaging on a shorter term than bonebeds, and exhibit a higher abundance of vertebrate skeletal elements than “background” sediments, leading to an intermediate abundance of bones and teeth within storm-related deposits of the shoreface and transition zone (e.g., hummocky-cross stratified sandstone (Shc) and massive sandstone (Sm) lithofacies).

### Preservational Bias, Isotaphonomy, and Implications for Marine Vertebrate Paleoecology

Patterns of taphonomic modification among taxa outlined above have implications for the study of paleoecology of marine vertebrates. The high frequency of abraded and fragmented vertebrate skeletal elements indicates a strong influence of physical processes that result in mechanical damage. The vertebrate sample from this study indicates that these processes result in a preservational bias against small, fragile elements such as odontocete and pinniped cranial and postcranial bones, and bony fish bones. Preliminary data from this analysis also suggest differential rates of disarticulation among different marine vertebrate groups; for example, small bodied marine birds and bony fish may disarticulate at a faster rate than marine mammals, possibly explaining this discrepancy; experimental studies bear this possibility out [15], [20]. Additionally, certain taxa are affected by phosphatization differently (Fig. 21), and as phosphatization may improve preservation potential, differential phosphatization may exaggerate the abundance of certain taxa within an assemblage. Any paleoecologic study concerning the relative abundance of marine vertebrates in shallow marine deposits should assess preservational bias, which has affected the Purisima Formation vertebrate sample to an unknown degree [136]. Given the propensity of studies indicating widespread and unpredictable taphonomic bias in the vertebrate fossil record, we advocate that taphonomic effects be treated as null hypotheses which must be excluded prior to paleoecologic interpretation of relative abundance data (“guilty of bias until proven otherwise”; [100] and references therein).

Previous studies aiming to abet problems of preservational bias for comparing relative abundance of different fossil assemblages have attempted to demonstrate that two assemblages may be “isotaphonomic” [136], [138], or distinguished by similar taphonomic characteristics, therefore having similar preservational biases. Demonstrating isotaphonomy has been elusive, and because preservational characteristics may vary widely even among the same type of skeletal concentration, comparing various taphonomic features of assemblages (e.g., abrasion, element size) is further necessary to establish (or discount) isotaphonomy [136]. It is apparent that preservational mode is variable between lithofacies of the Purisima Formation (Figs. 15–17), and thus it appears that even within the Purisima Formation, assemblages from different lithofacies are not isotaphonomic. As different biases occur in different depositional environments (e.g., higher rates of phosphatization, abrasion, and fragmentation within bonebeds and inner shelf settings, higher rates of articulation in middle and outer shelf settings; Fig. 32, 33), comparison of taxonomic relative abundance even between assemblages within a single formation are problematic.

A possible solution may be to restrict faunal comparisons within certain groups characterized by similar skeletal anatomy (e.g., Chondrichthyes, Pinnipedia), thus ameliorating the problem of preservational bias (even when comparing nonisotaphonomic assemblages), as advocated by Wilson [139]. Comparisons between groups are more problematic, given the effect of differential preservation. Furthermore, the element a certain taxon is identified from in an assemblage should be considered; even within broad taxonomic groups (e.g., Aves, Osteichthyes), the anatomical location of diagnostic features is variable across family, genus, and species-level taxa. For example, some pinnipeds are identifiable based on isolated teeth, while others are identifiable based on postcrania; different groups of birds are identified based on different bones of the skeleton (e.g., humerus, tarsometatarsus). If one element has a higher preservation potential than another, the abundance of a given taxon will be exaggerated.

Several trends in relative taxonomic abundance are evident from the various lithofacies of the Purisima Formation (Fig. 32). Birds are most abundant within sandstone lithofacies, and less common within the massive pebbly (Mpm) and massive mudrock (Mm) lithofacies (Fig. 32); this might suggest a higher abundance of birds in proximal settings, or alternatively a higher preservation potential in sandstones rather than mudrocks (note that the sample size for the Laminated Diatomite lithofacies is n = 6). Bony fish are most abundant in the hummocky cross-stratified sandstone (Shc) and massive pebbly sandstone (Spm) lithofacies (Fig. 32), and less abundant in the massive sandstone (Sm) and all three mudrock lithofacies (Mpm, Mm, Mld). Again, it is unclear whether this reflects genuine preference or greater numerical abundance within proximal settings, or if bony fish remains tend to be concentrated by shoreface sedimentologic processes and proximal bonebed formation. Odontocete remains are most abundant within the massive sandstone (Sm), hummocky cross-stratified sandstone (Shc), massive pebbly sandstone (Spm), and massive mudrock (Mm) lithofacies, and are less abundant in the massive pebbly mudrock (Mpm) lithofacies, and are slightly less well represented in the two bonebed lithofacies (Fig. 32). Baleen whale bones are most abundant within the Massive mudrock (Mm) lithofacies, and somewhat less common within the bonebed lithofacies (Mpm, Spm) and other sandstone lithofacies (Shc, Sm; Fig. 32). The significance of these patterns for cetaceans is unclear. Pinnipeds are unusually prevalent within the Massive Pebbly mudrock (Mpm) lithofacies at UCMP locality V99877 (Fig. 32); although this could be interpreted as proximity to terrestrial pinniped rookeries on a rocky shore or sea stacks, this lithofacies is deposited by bonebeds in the offshore (Fig. 12; see Lithofacies Analysis) and thus further from shore than facies of the Shoreface lithofacies association. Alternatively, this could reflect collecting bias at UCMP locality V99877, and it is unclear what significance this higher prevalence of pinnipeds represents. Indeterminate mammals occur only within the two bonebed lithofacies (Spm, Mpm) and massive sandstone (Mm) lithofacies (Fig. 32), perhaps being associated with lithofacies formed by processes of depositional hiatus; further sampling is expected to produce indeterminate mammal bone fragments from the hummocky cross-stratified sandstone (Shc) lithofacies.

### Sequence Stratigraphic Considerations

Several taphonomic trends are apparent when considered in the context of the sequence architecture of the Purisima Formation. The irregular lower contact of the Purisima Formation, identified as a sequence boundary, is mantled by clasts of the Santa Cruz Mudstone and abundant vertebrate skeletal remains (Bonebed 1); these skeletal elements were likely deposited during the sedimentary hiatus or eroded from the underlying formation. Bonebed 2 may represent a marine flooding surface (MFS) or a transgressive surface of erosion, and all the other bonebeds (Bonebeds 3–6, and possibly unstudied bonebeds) in the Purisima Formation appear at MFS discontinuities. The Maximum Marine Flooding Surface (MFFS) does not appear to be represented by a skeletal concentration (Fig. 4). Thus it appears that within the Purisima Formation, discontinuity surfaces with the largest offset in facies and highest magnitude record of nondeposition are systematically associated with laterally extensive marine vertebrate skeletal concentrations. This is in marked contrast to the relationship between discontinuity surfaces and skeletal concentrations in the late Cretaceous shallow marine record of Montana [101], where skeletal concentrations are patchy and developed only where underlying strata are fossiliferous. This led Rogers and Kidwell [101] to suggest that erosion and exhumation of bioclasts is the primary control on the genesis of skeletal concentrations in the marine realm. In the case of the Purisima Formation, all MFS and the sequence boundary (Bonebed 1) correspond to bonebeds. Two possibilities may explain this difference between the Purisima Formation and late Cretaceous marine strata in Montana. First, although many Purisima Formation bonebeds exhibit direct (preserved surfaces) or indirect (exhumed phosphatic intraclasts) evidence of erosion, perhaps a large component of the vertebrate concentrations is hiatal rather than erosional in origin. Second, the Purisima Formation was deposited in a basin with a moderate subsidence rate [2], [4], while most foreland basins (including the Western Interior foreland basin) are characterized by high subsidence rates [140].

Previous workers have identified a systematic relationship between invertebrate skeletal concentrations and sequence architecture in various Phanerozoic strata [2]–[4]. The Purisima Formation and other strata deposited under moderate subsidence settings exhibit “classic” sequence architecture and a variety of different invertebrate shelly concentrations (event, composite, hiatal, and lag) [2], [4]. Basins with lower subsidence rates (such as those along passive continental margins), represented by Cenozoic strata along the Atlantic coastal plain, exhibit “telescoped” transgressive and highstand systems tracts, relatively more widespread discontinuities that are also more time-rich, with more abundant hiatal and lag concentrations [2], [4]. and in strike-slip basins, discontinuities tend to truncate underlying facies across (or nearly across) the entire basin [137]. Basins deposited under high subsidence rates (e.g., late Neogene Imperial Group, California) are characterized by stretched sequences with poorly defined boundaries (due to discontinuous surfaces), and rare, locally restricted shellbeds predominantly comprising event and composite concentrations, with rare hiatal and lag concentrations confined to basin margins and paleohighs [2]–[4]. Unfortunately, few other broad scale studies of marine vertebrate taphonomy examining relationships between preservation and sequence anatomy exist, and comparisons with vertebrate preservation in low and high subsidence settings are currently not possible. However, this study does document a strong relationship between sequence architecture and vertebrate preservation in the Purisima Formation, an observation which parallels that reported for early marine vertebrates in the Ordovician of Ohio and sharks, bony fish, and marine mammals of the Eocene of Egypt [109], [141]. These relationships indicate that further investigation of marine vertebrate taphonomy within a sequence stratigraphic context is certainly warranted, and comparisons between different basin settings are likely to be informative.

Consideration of the age of some of these bonebeds permits evaluating them in the context of eustatic sea level change, as in sequence stratigraphy, stratigraphic diastems (e.g. transgressive lags) are linked to sea level rise [96], [142]. Three bonebeds within the Purisima Formation are well-constrained in terms of geochronologic age: Bonebed 1 (7.6–6.9 Ma; [35], [66]), Bonebed 4 (5.3 Ma; [35]), and Bonebed 6 (4.5–3.5 Ma; [35], [37]). Bonebed 6 appears to coincide with the latter part of a large magnitude, temporally long transgression from 5.6 Ma to 3.8 Ma [142]; however, the beginning of the transgression appears to predate the formation of Bonebed 6 by about 1.1 Ma. Bonebed 4, on the other hand, corresponds well with the beginning of this transgression. On the other hand, Bonebed 1, herein interpreted as a “forced regression”, coincides not with a regression but with a transgression, in contrast to stratigraphic evidence reported herein. Although stratigraphic diastems are widely interpreted as reflecting changes in sea level based on the sequence stratigraphic model, those within the Purisima Formation correspond poorly to eustatic trends. Local tectonics may outpace eustasy and may be implicated in the formation of unconformities [143], producing unconformities that temporally mismatch genuine eustatic-related unconformities. The Purisima Formation was deposited within a strike-slip basin [40], [137], which are characterized by discontinuities that extend across much of or nearly the entire basin [137]. In other strike-slip basins, local tectonics exerts a stronger control on stratigraphic architecture than eustasy [144]. Finally, other marine bonebeds have been convincingly linked with brief periods of rapid seafloor uplift [70]. Available evidence suggests that bonebeds within the Purisima Formation reflect local tectonic adjustments rather than changes in eustasy.

Additional trends are evident between the aforementioned discontinuities. Within parasequences of the HST, the overall abundance of vertebrate skeletal elements, thickness of shell beds, frequency of abrasion, and frequency of fragmentation increases up section (Fig. 33). This is related to the shallowing upward nature of HST parasequences. The best example is reflected in the parasequence preserved between Bonebed 5 and 6; the massive sandstone and mudrock in the lower part exhibit rare, isolated (and occasional associated or articulated) bones and skulls that generally lack abrasion or fragmentation, while the upper hummocky cross-stratified sandstone towards the top yields isolated fragmented, abraded bones that occur within shell beds. The two parasequences within the TST exhibit the opposite trend (abrasion, fragmentation decreasing upsection and articulation increasing upsection), and this is related to a deepening upward trend within the TST.

Altogether, a taphonomic cross section of the Purisima Formation results in a picture that is similar to the sequence stratigraphic model [96]; laterally extensive bonebeds (Bonebed taphofacies) mark most of the major discontinuities within the Purisima Formation (Fig. 33, 34). The discontinuity-bounded packages of rock include Taphofacies 1–3, which laterally interfinger with one another from proximal to distal (respectively; Fig. 33, 34). Furthermore, discontinuities within the HST typically reflect a change from proximal to distal taphofacies (most often Taphofacies 1 below and Taphofacies 2 above; Fig. 33, 34). This is reversed within the TST.

## Conclusions

1. Lithofacies analysis of the Santa Cruz section of the Purisima Formation indicates the presence of six lithofacies, which interfinger and represent shoreface, transition zone, and offshore deposition. Sandstone lithofacies record high-energy deposition and non-deposition (hiatus) in shoreface to offshore environments; mudrock lithofacies record offshore deposition through suspension settling in low energy settings. Application of sequence stratigraphic methods identifies the lower contact of the Purisima Formation as a type 2 sequence boundary, overlain by a thin transgressive systems tract, while the rest of the overlying portion of the Santa Cruz section represents stacked prograding parasequences of the highstand systems tract.
2. A number of laterally extensive bonebeds in the Purisima Formation correspond to major discontinuity surfaces. The lower two bonebeds represent a type 2 sequence boundary and transgressive surface of erosion (respectively), while the rest represent marine flooding surfaces within a highstand systems tract. The maximum marine flooding surface exhibits no vertebrate skeletal concentration. Many bonebeds that lack an erosional surface exhibit indirect evidence of erosion, indicating that pervasive bioturbation has erased the erosional surface; this has implications for discerning between hiatal and lag concentrations in the rock record. Vertebrate skeletal elements from bonebed lithofacies consistently exhibit the most taphonomic modification among the lithofacies.
3. Vertebrate skeletal material from non-bonebed lithofacies decreases in abundance distally and displays a clear pattern of onshore-offshore gradients in preservation. Abrasion of elements is highest in shoreface depositional settings, gradually decreasing towards the offshore. Fragmentation follows a similar trend. Articulated or associated skeleton abundance (relative to isolated elements) is higher in offshore environments. Phosphatized vertebrate material is most abundant within the lower shoreface and transition zone, decreasing in abundance both proximally (upper shoreface) and distally (offshore), suggesting the transition zone harbors the optimal set of conditions for preservation: sufficient sediment starvation with infrequent large scale storms that erode the seafloor. The majority of polished elements are phosphatized, demonstrating a clear link between polish and ‘prefossilization’.
4. Four taphofacies were recognized within the Purisima Formation. Taphofacies 1, 2, and 3 are defined by varying proportions of abrasion, fragmentation, articulation, and phosphatization, and document a continuum of preservational trends. These trends are related to sediment transport processes and sedimentary budget. The Bonebed taphofacies truncates underlying taphofacies, and within the highstand systems tract, generally divides zones of poor fossil preservation in underlying strata (typical of upshelf, relatively higher energy settings) from better fossil preservation (typical of downshelf relatively lower energy settings) in overlying strata. This can be viewed as a taphonomic expression of transgression. Within the transgressive systems tract, the opposite pattern occurs. This taphofacies model allows marine vertebrate fossils to be firmly placed within a holistic taphonomic framework for the first time.

## Supporting Information

Table S1 Table of raw taphonomic data analyzed in this study.
(XLS)
